# Evolutionary dynamics of bacteria in the gut microbiome within and across hosts

**DOI:** 10.1371/journal.pbio.3000102

**Published:** 2019-01-23

**Authors:** Nandita R. Garud, Benjamin H. Good, Oskar Hallatschek, Katherine S. Pollard

**Affiliations:** 1 Gladstone Institutes, San Francisco, California, United States of America; 2 Department of Physics, University of California, Berkeley, Berkeley, California, United States of America; 3 Department of Bioengineering, University of California, Berkeley, Berkeley, California, United States of America; 4 Kavli Institute for Theoretical Physics, University of California, Santa Barbara, Santa Barbara, California, United States of America; 5 Department of Integrative Biology, University of California, Berkeley, Berkeley, California, United States of America; 6 Department of Epidemiology and Biostatistics, Institute for Human Genetics, Quantitative Biology Institute, and Institute for Computational Health Sciences, University of California, San Francisco, San Francisco, California, United States of America; 7 Chan-Zuckerberg Biohub, San Francisco, California, United States of America; Instituto Gulbenkian de Ciencia, PORTUGAL

## Abstract

Gut microbiota are shaped by a combination of ecological and evolutionary forces. While the ecological dynamics have been extensively studied, much less is known about how species of gut bacteria evolve over time. Here, we introduce a model-based framework for quantifying evolutionary dynamics within and across hosts using a panel of metagenomic samples. We use this approach to study evolution in approximately 40 prevalent species in the human gut. Although the patterns of between-host diversity are consistent with quasi-sexual evolution and purifying selection on long timescales, we identify new genealogical signatures that challenge standard population genetic models of these processes. Within hosts, we find that genetic differences that accumulate over 6-month timescales are only rarely attributable to replacement by distantly related strains. Instead, the resident strains more commonly acquire a smaller number of putative evolutionary changes, in which nucleotide variants or gene gains or losses rapidly sweep to high frequency. By comparing these mutations with the typical between-host differences, we find evidence that some sweeps may be seeded by recombination, in addition to new mutations. However, comparisons of adult twins suggest that replacement eventually overwhelms evolution over multi-decade timescales, hinting at fundamental limits to the extent of local adaptation. Together, our results suggest that gut bacteria can evolve on human-relevant timescales, and they highlight the connections between these short-term evolutionary dynamics and longer-term evolution across hosts.

## Abstract 2

A population genetic analysis of human gut metagenomes reveals short-term evolution within individual species of gut bacteria, both within individual hosts, and across the larger population.

## Author summary

The human gut harbors a diverse microbial community whose composition is shaped by a variety of ecological forces. Given the high rates of turnover, the residents of this community might also have the opportunity to evolve over time by acquiring heritable changes to their genomes. Yet, despite the potential importance of these effects, we currently know very little about the evolutionary dynamics that occur within species in this complex community. Here, we introduce a new approach for extracting evolutionary signals from a large panel of human gut metagenomes and for interpreting these signals using simple null models from population genetics. We use this approach to quantify the evolutionary dynamics of approximately 40 prevalent species of gut bacteria, both within individual hosts and across the larger population. We find that resident populations of gut bacteria can evolve within their hosts on short timescales, but after many years, the resident populations are typically replaced by distantly related strains. The patterns of variation across hosts indicate widespread recombination within species, but the quantitative signals suggest interesting departures from traditional population genetic models. Together, these results show that short-term evolution in the gut microbiome may be more complex and widespread than is often assumed.

## Introduction

The gut microbiome is a complex ecosystem comprised of a diverse array of microbial organisms. The abundances of different species and strains can vary dramatically based on diet [1], host species [2], and the identities of other co-colonizing taxa [3]. These rapid shifts in community composition suggest that individual gut microbes may be adapted to specific environmental conditions, with strong selection pressures between competing species or strains. Yet, while these ecological responses have been extensively studied, much less is known about the evolutionary forces that operate within populations of gut bacteria, both within individual hosts and across the larger host-associated population. This makes it difficult to predict how rapidly strains of gut microbes will evolve new ecological preferences when faced with environmental challenges, such as drugs or diet, and how the genetic composition of the community will change as a result.

The answers to these questions depend on two different types of information. At a mechanistic level, one must understand the functional traits that are under selection in the gut and how they may be modified genetically. Recent work has started to address this question, leveraging techniques from comparative genomics [4–6], evolution in model organisms [7–9], and high-throughput genetic screens [10, 11]. Yet, in addition to the targets of selection, evolution also depends on population genetic processes that describe how mutations spread through a population of gut bacteria, both within individual hosts and across the larger population. These dynamical processes can strongly influence which mutations are likely to fix within a population, and the levels of genetic diversity that such populations can maintain. Understanding these processes is the goal of our present work.

Previous studies of pathogens [12], laboratory evolution experiments [13], and some environmental communities [14–17] have shown that microbial evolutionary dynamics are often dominated by rapid adaptation, with new variants accumulating within months or years [7, 14, 18–25]. However, it is not clear how this existing picture of microbial evolution extends to a more complex and established ecosystem like the healthy gut microbiome. On the one hand, hominid gut bacteria have had many generations to adapt to their host environment [26], and they may not be subjected to the same immune pressures as pathogens. The large number of potential competitors in the gut ecosystem may also provide fewer opportunities for a strain to adapt to new conditions before an existing strain expands to fill the niche [27, 28] or a new strain invades from outside the host. On the other hand, it is also possible that small-scale environmental fluctuations, either driven directly by the host or through interactions with other resident strains, might increase the opportunities for local adaptation [29]. If immigration is restricted, the large census population size of gut bacteria could allow residents to produce and fix adaptive variants rapidly before a new strain is able to invade. In this case, one could observe rapid adaptation on short timescales, which is eventually arrested on longer timescales as strains are exposed to the full range of host environments. Additional opportunities for adaptation can occur if the range of host environments also shifts over time (e.g., due to urbanization, antibiotic usage, etc.). Determining which of these scenarios apply to gut communities is critical for efforts to study and manipulate the microbiome.

While traditional amplicon sequencing provides limited resolution to detect within-species evolution [30], whole-genome shotgun metagenomic sequencing is starting to provide the raw polymorphism data necessary to address these questions [31]. In particular, several reference-based approaches have been developed to detect genetic variants within individual species in larger metagenomic samples [31–36]. While these approaches enable strain-level comparisons between samples, they have also documented substantial within-species variation in individual metagenomes [31, 35, 37]. This makes it difficult to assign an evolutionary interpretation to the genetic differences between samples, because they arise from unobserved mixtures of different bacterial lineages.

Several approaches have been developed to further resolve these mixed populations into individual haplotypes or "strains." These range from simple consensus approximations [35, 37, 38], to sophisticated clustering algorithms [39, 40] and the incorporation of physical linkage information [41]. However, while these methods are useful for tracking well-defined strains across samples, it is not known how their assumptions and failure modes might bias inferences of evolutionary dynamics, particularly among closely related strains. As a result, the evolutionary processes that operate within species of gut bacteria remain poorly characterized.

In this study, we take a different approach to the strain detection problem that is specifically designed for inferring evolutionary dynamics in a large panel of metagenomes. Building on earlier work by [4, 35], we show that many prevalent species have a subset of hosts for which a portion of the dominant lineage is much easier to identify. By focusing only on this subset of samples, we develop methods for resolving small differences between the dominant lineages with a high degree of confidence.

We use this approach to analyze a large panel of publicly available human stool samples [42–46], which allows us to quantify evolutionary dynamics within and across hosts in approximately 40 prevalent bacterial species. We find that the long-term evolutionary dynamics across hosts are broadly consistent with models of quasi-sexual evolution and purifying selection, with relatively weak geographic structure in many prevalent species. However, our quantitative approach also reveals interesting departures from standard population genetic models of these processes, suggesting that new models are required to fully understand the evolutionary dynamics that take place across the larger population.

We also use our approach to detect examples of within-host adaptation, in which nucleotide variants or gene gains or losses rapidly sweep to high frequency on 6-month timescales. We find evidence that some within-host sweeps may be seeded by recombination, in addition to de novo mutations, as might be expected for a complex ecosystem with frequent horizontal exchange. However, by analyzing differences between adult twins, we find that short-term evolution can eventually be overwhelmed by the invasion of distantly related strains on multi-decade timescales. This suggests that resident strains are rarely able to become so well adapted to a particular host that they prevent future replacements. Together, these results show that the gut microbiome is a promising system for studying the dynamics of microbial evolution in a complex community setting. The framework we introduce may also be useful for characterizing evolution of microbial communities in other environments.

## Materials and methods

### Resolving within-host lineage structure in a panel of metagenomic samples

To investigate evolutionary dynamics within species in the gut microbiome, we analyzed shotgun metagenomic data from a panel of stool samples from 693 healthy individuals sequenced in previous work (S1 Table). This panel includes 250 North American subjects sequenced by the Human Microbiome Project (HMP) [42, 44], a subset of which were sampled at 2 or 3 time points roughly 6–12 months apart. To probe within-host dynamics on longer timescales, we also included data from a cohort of 125 pairs of adult twins from the TwinsUK registry [45], and 4 pairs of younger twins from [46]. As we describe below, the differences between these cohorts provide a proxy for the temporal changes that accumulate in adult twins over longer timescales. Finally, to further control for geographic structure, we also included samples from 185 Chinese subjects sequenced at a single time point [43].

We used a standard reference-based approach to measure single nucleotide variant (SNV) frequencies and gene copy number across a panel of prevalent species for each metagenomic sample (see S1A Text for details on the bioinformatic pipeline, including mapping parameters and other filters). Descriptive summaries of this genetic variation have been reported elsewhere [31, 33–35, 37, 44]. Here, we revisit these patterns to investigate how they emerge from the lineage structure set by the host colonization process. Using these results, we then show how certain aspects of this lineage structure can be inferred from the statistics of within-host polymorphism, which enable measurements of evolutionary dynamics across samples.

**Fig 1:**
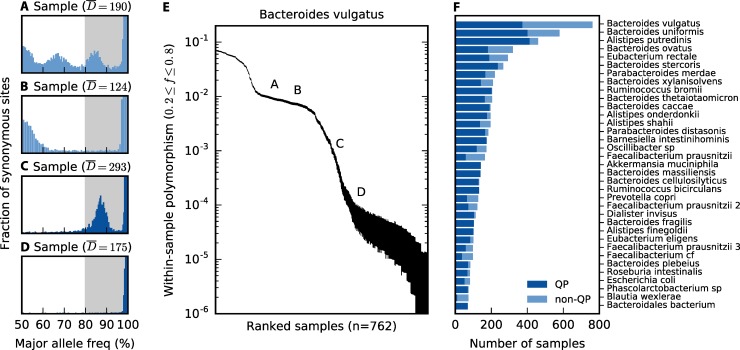
Genetic diversity within hosts. *Bacteroides vulgatus* is shown as an example in panels A–E; examples for 24 other species are shown in S1 Fig, S2 Fig, and S3 Fig. (A–D) The distribution of major allele frequencies at synonymous sites in the core genome for four different samples, with the median read depth $\bar{D}$ listed above each panel. Major allele frequencies are estimated by max{*f*,1−*f*}, where *f* is the frequency of the base on the reference genome (S1A Text, part iii). To emphasize the distributional patterns, the vertical axis is scaled by an arbitrary normalization constant in each panel, and it is truncated for visibility. The white region denotes the intermediate frequency range used for the polymorphism calculations below. (E) The average fraction of synonymous sites in the core genome with major allele frequencies ≤80% (white region in A–D), for all samples with $\bar{D}\geq 20$. Vertical lines denote 95% posterior confidence intervals based on the observed number of counts (S1B Text). The letters indicate the corresponding values for the samples in panels (A–D) for comparison. (F) The distribution of quasi-phaseable (QP) samples among the 35 most prevalent species, arranged by descending prevalence; the distribution across hosts is shown in S7 Fig. For comparison, panels (C) and (D) are classified as QP, while panels (A) and (B) are not.

As an illustrative example, we first focus on the patterns of polymorphism in *Bacteroides vulgatus*, which is among the most abundant and prevalent species in the human gut. These properties ensure that the *B*. *vulgatus* genome has high coverage in many samples, which enables more precise estimates of the allele frequencies in each sample (Fig 1A–1D). The overall levels of within-host diversity for this species are summarized in Fig 1E, based on the fraction of synonymous sites in core genes with intermediate allele frequencies (white region in Fig 1A–1D). This measure of within-host genetic variation varies widely across the samples: some metagenomes have only a few variants along the *B*. *vulgatus* genome, while others have mutations at more than 1% of all synonymous sites (comparable to the differences between samples, S5 Fig). Similar patterns are observed in many other prevalent species (S3 Fig).

We first asked whether these patterns are consistent with a model in which each host is colonized by a single *B*. *vulgatus* clone, so that the intermediate frequency variants represent mutations that have arisen since colonization. Using conservatively high estimates for per-site mutation rates (*μ*~10^−9^ [47]), generation times (approximately 10 per day [48]), and time since colonization (<100 years), this model predicts that the neutral polymorphism rate at synonymous sites should be no greater than 0.1% (S1B Text, part ii). This is at odds with the higher levels of diversity observed in many samples (Fig 1E and S3 Fig). Instead, we conclude that the samples with higher synonymous diversity have been colonized by multiple divergent bacterial lineages that accumulated mutations for many generations before coming together in the same gut community.

As a plausible alternative, we next asked whether the data are consistent with a large number of colonizing lineages (*n*_*c*_≫1) drawn at random from the broader population. However, this process is expected to produce fairly consistent polymorphism rates and allele frequency distributions in different samples, which is at odds with the variability we observe even among the high-diversity samples (e.g., Fig 1A, 1B, S1 Fig and S2 Fig). Instead, we hypothesize that many of the high-diversity hosts have been colonized by just a few diverged lineages [i.e., $\left(n_{c}-1)∼O(1\right)$]. Consistent with this hypothesis, the distribution of allele frequencies in each host is often strongly peaked around a few characteristic frequencies, suggesting a mixture of several distinct lineages (Fig 1A–1C, S1 Fig and S2 Fig). Similar findings have recently been reported in a number of other host-associated microbes, including several species of gut bacteria [4, 35, 49, 50]. Fig 1A–1C shows that hosts can vary both in the apparent number of colonizing lineages and the frequencies at which they are mixed together. As a result, we cannot exclude the possibility that even the low-diversity samples (e.g., Fig 1D) are colonized by multiple lineages that happen to fall below the detection threshold set by the depth of sequencing.

### Quasi-phaseable samples

Compared with the extreme cases of single-colonization (*n*_*c*_ = 1) or colonization by many strains (*n*_*c*_≫1), it is more difficult to identify evolutionary changes between lineages when there are only few strains at intermediate frequency. In this scenario, within-host populations are not clonal, but the corresponding allele frequencies derive from idiosyncratic colonization processes rather than a large random sample from the population (as, e.g., in [16]). To disentangle genetic changes between lineages from these host-specific factors, we must estimate phased haplotypes (or "strains") from the distribution of allele frequencies within individual hosts. This is a complicated inverse problem, and we will not attempt to solve the general case here. Instead, we adopt an approach similar to [35] and others, and leverage the fact that the lineage structure in certain hosts is sufficiently simple that we can assign alleles to the dominant lineage with a high degree of confidence.

Our approach is based on the simple observation that two high-frequency variants must co-occur in an appreciable fraction of cells (S1C Text, part i). This "pigeonhole principle" suggests that we can estimate the genotype of one of the lineages in a mixed sample by taking the major alleles present above some threshold frequency, f*≫50%, and treating the remaining sites as missing data. Although the potential errors increase with the length of the inferred haplotype, we will not actually require genome-length haplotypes for our analysis here. Instead, we leverage the fact that significant evolutionary information is already encoded in the marginal distributions of one- and two-site haplotypes, so that these "quasi-phased" lineages will be sufficient for our present purposes.

The major challenge with this approach is that we do not observe the true allele frequency directly but must instead estimate it from a noisy sample of sequencing reads. This can lead to phasing errors when the true major allele is sampled at low frequency by chance and is assigned to the opposite lineage (S4 Fig). We will refer to these as "polarization errors," because they stem from an incorrect inference of the major allele. The probability of a polarization error will vary dramatically depending on the sequencing coverage and the true frequency of the major allele (S1C Text, part ii). Previous approaches based on consensus alleles [35, 37] can therefore induce an unknown number of errors that make it difficult to confidently detect a small number of evolutionary changes between samples.

In S1C Text, we show that by explicitly modeling the sampling error process, the expected probability of a polarization error in our cohort can be bounded to be sufficiently low if we take f* = 80%, and if we restrict our attention to samples with sufficiently high coverage and sufficiently low rates of intermediate-frequency polymorphism. We will refer to these as quasi-phaseable (QP) samples. In the *B*. *vulgatus* example above, Fig 1C and 1D are classified as QP, while Fig 1A and 1B are not. Note that quasi-phaseability is separately defined for each species in a metagenomic sample, rather than for the sample as a whole. For simplicity, we will still refer to these species-sample combinations as QP samples, with the implicit understanding that they refer to a particular focal species.

In Fig 1F, we plot the distribution of QP samples across the most prevalent gut bacterial species in our panel. The fraction of QP samples varies between species, ranging from about 50% in the case of *Prevotella copri* to nearly 100% for *B*. *fragilis* [4], and it accounts for much of the variation in the average polymorphism rate between species (S6 Fig). Most individuals carry a mixture of QP and non-QP species (S7 Fig), suggesting that quasi-phaseability arises independently for each species in a sample, rather than for the sample as a whole. Thus, although many species-sample combinations are not QP, our approximately 500-sample cohort still contains tens to hundreds of QP samples in many prevalent species, yielding about 3,000 quasi-phased haplotypes in total. Consistent with previous studies of the stability of personal microbiomes [31, 35, 51], a majority of the longitudinally sampled species maintain their QP classification at both time points, although this pattern is not universal (S8 Fig). We will revisit the peculiar properties of this within-host lineage distribution in “Discussion.” For the remainder of the analysis, we will take the distribution in Fig 1F as given and focus on leveraging the QP samples to quantify the evolutionary changes that accumulate between lineages in different samples.

We investigate two types of evolutionary changes between lineages in different QP samples. The first class consists of single nucleotide differences, which are defined as SNVs that segregate at frequencies ≤1−f* in one sample and ≥f* in another, with f*≈80% as above (S4 Fig). These thresholds are chosen to ensure a low genome-wide false positive rate given the typical coverage and allele frequency distributions among the QP samples in our panel (S1C Text, part iv). The second class consists of differences in gene presence or absence, in which the relative copy number of a gene, *c*, is below the threshold of detection (*c*<0.05) in one sample and is consistent with a single-copy gene (0.6<*c*<1.2, see S9 Fig) in the other sample. These thresholds are chosen to ensure a low genome-wide false positive rate across the QP samples, given the typical variation in sequencing coverage along the genome (S1C Text, part v), and to minimize mapping artifacts (S1A Text, part ii).

Note that these SNV and gene changes represent only a subset of the potential differences between lineages. We neglect other evolutionary changes (e.g., indels, genome rearrangements, or changes in high copy number genes) that are more difficult to quantify in a metagenomic sample, as well as more subtle changes in allele frequency and gene copy number that do not reach our stringent detection thresholds. We will revisit these and other limitations in more detail in “Discussion”.

## Results

### Long-term evolution across hosts

By focusing on the QP samples for each species, we can measure genetic differences between lineages in different hosts, as well as within hosts over short time periods. Descriptive summaries of this variation have been reported elsewhere [31, 33–35, 37, 44]. Here, we aim to leverage these patterns (and the increased resolution of the QP samples) to quantify the evolutionary dynamics that operate within species of gut bacteria, both within and across hosts.

**Fig 2:**
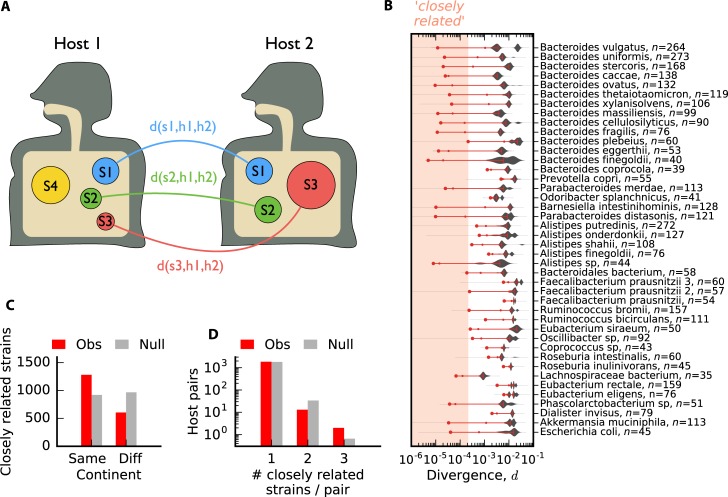
Between-host divergence across prevalent species of gut bacteria. (A) Schematic illustration. For a given pair of hosts (h1, h2), core-genome nucleotide divergence (*d*) is computed for each species (s1, s2, etc.) that is quasi-phaseable (QP) in both hosts. (B) Distribution of *d* across all pairs of unrelated hosts for a panel of prevalent species. Species are sorted according to their phylogenetic distances [33], with the number of QP hosts indicated in parentheses; species were only included if they had at least 33 QP hosts (>500 QP pairs). Symbols denote the median (dash), 1 percentile (small circle), and 0.1 percentile (large circle) of each distribution and are connected by a red line for visualization; for distributions with <10^3^ data points, the 0.1 percentile is estimated by the second-lowest value. The shaded region denotes our ad hoc definition of "closely related" divergence, *d*≤2×10^−4^. (C) The distribution of the number of species with closely related strains in distinct hosts present in the same or different continents. The null distribution is obtained by randomly permuting hosts within each species. Although the observed values are significantly different than the null (*P*<10^−4^), the large contribution from different continents shows that closely related strains are not solely a product of geographic separation. (D) The distribution of the number of species with closely related strains for each pair of hosts. The null distribution is obtained by randomly permuting hosts independently within each species (*n* = 10^3^ permutations, *P*≈0.9). This shows that there is no tendency for the same pairs of hosts to have more closely related strains than expected under the null distribution above.

To interpret within-host changes in an evolutionary context, it will be useful to first understand the structure of genetic variation between lineages in different hosts. This variation reflects the long-term population genetic forces that operate within each species, presumably integrating over many rounds of colonization, growth, and dispersal. To investigate these forces, we first analyzed the average nucleotide divergence between strains of a given species in different pairs of QP hosts (Fig 2A). In the case of twins, we included only a single host from each pair, to better approximate a random sample from the population.

Fig 2B shows the distribution of pairwise divergence, averaged across the core genome, for about 40 of the most prevalent bacterial species in our cohort. In a panmictic, neutrally evolving population, we would expect these distances to be clustered around their average value, *d*≈2*μT*_*c*_, where *T*_*c*_ is the coalescent timescale for the across-host population [52]. By contrast, Fig 2 shows striking differences in the degree of relatedness for strains in different hosts. Even at this coarse, core-genome-wide level, the genetic distances vary over several orders of magnitude.

Some species show multiple peaks of divergence for high values of *d*, consistent with the presence of subspecies [36], ecotypes [53, 54], or other strong forms of population structure. These coarse groupings have been observed previously and are not our primary focus here. Rather, we seek to understand the population genetic forces that operate at finer levels of taxonomic resolution.

From this perspective, the more surprising parts of Fig 2 are the thousands of pairs of lineages with extremely low between-host divergence (e.g., *d*≲0.01%), more than an order of magnitude below the median values in most species. Similar observations have recently been reported by [35] and are often interpreted as strain sharing across hosts. However, the evolutionary interpretation of these closely related strains remains unclear.

### Closely related strains reflect population genetic processes, rather than cryptic host relatedness

The simplest explanation for a long tail of closely related strains is cryptic relatedness [55], arising from a breakdown of random sampling. For microbes, this can occur when two cells are sampled from the same clonal expansion, e.g., when strains are transferred between mothers and infants [33, 56], between cohabitating individuals [46], or within a hospital outbreak [57]. While these transmission events have been observed in other studies, they are unlikely to account for the patterns here. All of the lineages in Fig 2 are sampled from individuals in different households, and more than a third of the closely related pairs derive from individuals on different continents (Fig 2B).

Of course, there could still be some other geographic variable, beyond household or continent of origin, that could explain an elevated probability of transmission between two individuals. Fortunately, our metagenomic approach allows us to rule out these additional sources of cryptic host relatedness by leveraging multiple species comparisons for the same pair of hosts. If there were a hidden geographic variable, then we would expect that individuals with closely related strains in one species would be much more likely to share closely related strains in other species as well. However, we observe only a small fraction of hosts that share multiple closely related strains (Fig 2C), consistent with a null model in which these strains are randomly and independently distributed across hosts. This suggests that host-wide sampling biases are not the primary driver of the closely related strains in Fig 2.

Although the rates of nucleotide divergence are low, the vast majority of these strains are still genetically distinguishable from each other. The absolute number of SNV differences typically exceeds our estimated false positive rate (S10A Fig, S1C Text, part iv), and these SNV differences are often accompanied by ≳10 differences in gene content (S10B Fig). Furthermore, we found that closely related strains frequently differed in their collections of private marker SNVs (S11 Fig), which are often used to track strain transmission events [33, 46]. Together, these lines of evidence suggest that closely related strains are often genetically distinct and do not arise from a simple clonal expansion. Instead, the data suggest that there are additional population genetic timescales beyond *T*_*c*_ that are relevant for microbial evolution.

This hypothesis is bolstered by the large number of species, particularly in the *Bacteroides* genus, with anomalously low divergence rates between some pairs of hosts. However, we note that this pattern is not universal: some genera, like *Alistipes* or *Eubacterium*, show more uniform rates of divergence between hosts. Apart from these phylogenetic correlations, we cannot yet explain why some species have low-divergence host pairs and others do not. Natural candidates such as sample size, abundance, vertical transmissibility [33], or sporulation score [58] struggle to explain the differences between *Bacteroides* and *Alistipes* species.

### Closely related strains have distinct signatures of natural selection

We next examined how natural selection influences the genetic diversity observed between hosts. Previous work has suggested that genetic diversity in many species of gut bacteria is strongly constrained by purifying selection, which purges deleterious mutations that accumulate between hosts [31]. However, the temporal dynamics of this process remain poorly understood. We do not know whether purifying selection acts quickly enough to prevent deleterious mutations from spreading to other hosts, or if deleterious mutations typically spread across multiple hosts before they are purged. In addition, it is plausible that the dominant mode of natural selection could be different for the closely related strains above (e.g., if they reflect recent ecological diversification [15]).

To address these questions, we analyzed the relative contribution of synonymous and nonsynonymous mutations that comprise the overall divergence rates in Fig 2A. We focused on the ratio between the per-site divergence at nonsynonymous sites (*d*_*N*_) and the corresponding value at synonymous sites (*d*_*S*_). Under the assumption that synonymous mutations are effectively neutral, the ratio *d*_*N*_/*d*_*S*_ measures the average action of natural selection on mutations at nonsynonymous sites.

**Fig 3:**
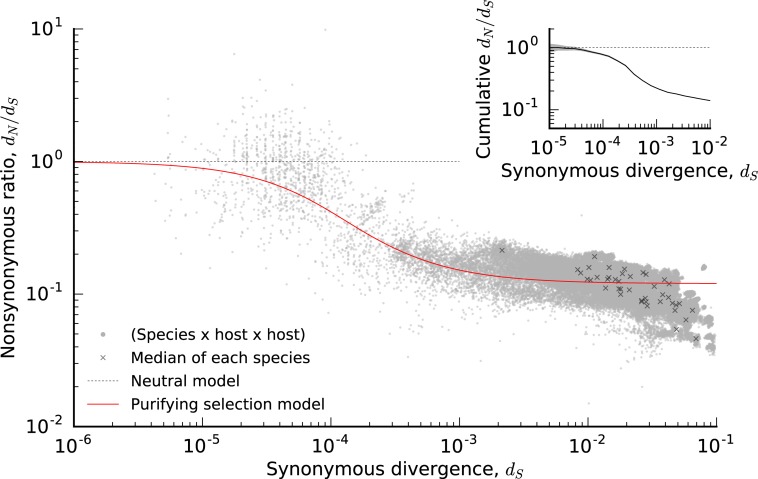
Signatures of selective constraint within species as a function of core-genome divergence. Ratio of divergence at nondegenerate nonsynonymous sites (*d*_*N*_) and 4-fold degenerate synonymous sites (*d*_*S*_) as a function of *d*_*S*_ (S1D Text) for all species × host1 × host2 combinations in Fig 2 (gray circles). Crosses (*x*) denote species-wide estimates obtained from the ratio of the median *d*_*N*_ and *d*_*S*_ within each species. The red line denotes the theoretical prediction from the purifying selection null model in S1D Text. Inset shows the ratio between the cumulative private *d*_*N*_ and *d*_*S*_ values for all quasi-phaseable host pairs with core-genome-wide synonymous divergence less than *d*_*S*_. The narrow shaded region denotes 95% confidence intervals estimated by Poisson resampling (S1D Text), which shows that *d*_*N*_/*d*_*S*_≲1, even for low *d*_*S*_.

In Fig 3, we plot these *d*_*N*_/*d*_*S*_ estimates across every pair of QP hosts for each of the prevalent species in Fig 2A. The values of *d*_*N*_/*d*_*S*_ are plotted as a function of *d*_*S*_, which serves as a proxy for the average divergence time across the genome. We observe a consistent negative relationship between these two quantities across the prevalent species in Fig 2.

For large divergence times (*d*_*S*_~1%), we observe only a small fraction of nonsynonymous mutations (*d*_*N*_/*d*_*S*_~0.1), indicating widespread purifying selection on amino acid replacements [31]. Yet, among more closely related strains, we observe a much higher fraction of nonsynonymous changes, with *d*_*N*_/*d*_*S*_ approaching unity when *d*_*S*_~0.01% (we observe a similar trend if we restrict our attention to singleton SNVs, S12 Fig). Moreover, this negative relationship between *d*_*N*_/*d*_*S*_ and *d*_*S*_ is much more pronounced than the between-species variation in the typical values of *d*_*N*_/*d*_*S*_ (black crosses in Fig 3). While between-species variation may be driven by mutational biases, the strong within-species signal indicates that there are consistent differences in the action of natural selection as a function of time.

In principle, the *d*_*N*_/*d*_*S*_ increases in the recent past could be driven by interesting biological processes, such as enhanced adaptation or ecological diversification on short timescales, or a recent global shift in selection pressures caused by host-specific factors (e.g., the introduction of agriculture). However, the data in Fig 3 appear to be well explained by an even simpler null model of purifying selection, in which deleterious mutations are purged over a timescale inversely proportional to their cost (S1D Text). This dynamical model can explain the varying signatures of natural selection without requiring that the selective pressures themselves vary over time. We find reasonable quantitative agreement for a simple distribution of fitness effects, in which 10% of nonsynonymous sites are neutral and the remaining 90% have fitness costs on the order of *s*/*μ*~10^5^. Although the true model is likely more complicated, we argue that this simple null model should be excluded before more elaborate explanations are considered.

For example, unambiguous proof of recent adaptation could be observed if *d*_*N*_/*d*_*S*_ consistently exceeded 1 among the most closely related strains (because this can only occur by chance under purifying selection). While a few of the individual comparisons in Fig 3A have *d*_*N*_/*d*_*S*_>1, the cumulative version in Fig 3B shows that *d*_*N*_/*d*_*S*_ does not significantly exceed 1, even for the lowest values of *d*_*S*_. This suggests that, if positive selection is present, it is not sufficiently widespread to overpower the signal of purifying selection in these global *d*_*N*_/*d*_*S*_ measurements. However, there is also substantial variation around the average trend in Fig 3, which could hide important biological variation among species (or among different genomic regions in the same species). Resolving the signatures of natural selection at these finer scales remains an important avenue for future work.

#### Quasi-sexual evolution on intermediate timescales

In principle, the large range of genome-wide divergence in Figs 2 and 3 could arise in a model with strong population structure, in which all but the most closely related strains are genetically isolated from each other [59]. Such isolation can be driven by geography as well as ecological diversification [15]. Here, we leverage our quasi-phasing approach to show that genetic isolation cannot account for the patterns in Figs 2 and 3. Instead, we find that the core genomes of many prevalent gut bacterial species evolve in a "quasi-sexual" manner [16], with frequent genetic exchange among individual strains.

**Fig 4:**
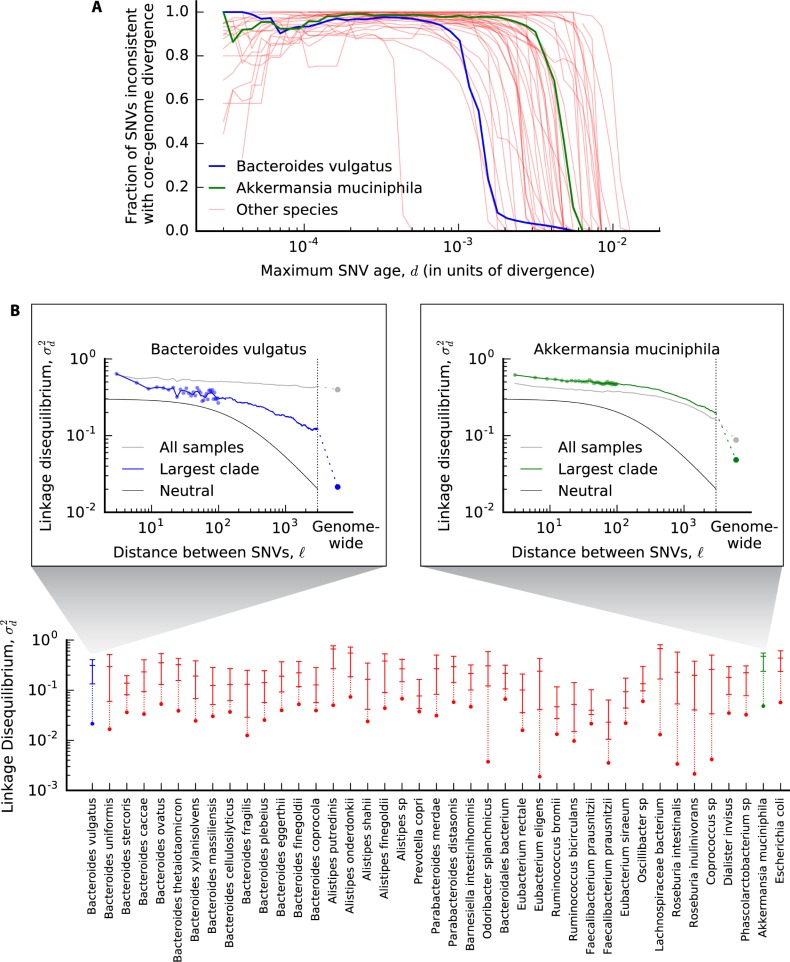
Recombination between strains across hosts. (A) Phylogenetic inconsistency between individual single nucleotide variants (SNVs) and core-genome-wide divergence for each of the species in Fig 2. The fraction of inconsistent SNVs is plotted for all 4-fold degenerate synonymous SNVs in the core genome with estimated age ≤*d* (S1E Text, part i). Singleton SNVs are excluded, because inconsistency can only be assessed for SNVs with ≥2 minor alleles. (B, inset) Linkage disequilibrium (LD) ($\sigma_{d}^{2}$) as a function of distance (l) between pairs of 4-fold degenerate synonymous sites in the same core gene (S1F Text). Individual data points are shown for distances <100 bp, while the solid line shows the average in sliding windows of 0.2 log units. The gray line indicates the values obtained without controlling for population structure, while the blue line is restricted to the largest top-level clade (S2 Table, S1E Text, part ii). The solid black line denotes the neutral prediction from S1F Text; the only free parameters in this model are vertical and horizontal scaling factors, which have been shifted to enhance visibility. For comparison, the core-genome-wide estimate for SNVs in different genes is depicted by the dashed line and circle. (B) Summary of LD in the largest top-level clade for all species with ≥10 quasi-phaseable hosts. Species are sorted phylogenetically as in Fig 2B. For each species, the three dashes denote the value of $\sigma_{d}^{2}(l)$ for intragenic distances of l=9, 99, and 2,001 bp, respectively, while the core-genome-wide values are depicted by circles. Points belonging to the same species are connected by vertical lines for visualization.

Recombination alters the genealogical relationships between strains in different portions of the genome [52]. We therefore sought evidence for recombination by searching for inconsistencies between the genealogies encoded in individual SNVs and those encoded in the genome-wide divergences in Fig 2. To do so, we developed an approach for directly quantifying phylogenetic inconsistency between individual SNVs and the pairwise divergence distribution in Fig 2B, without requiring a full genealogical reconstruction (S13 Fig, S1E Text, part i). This method also provides an estimate of the maximum age of each SNV (in divergence units), assuming purely clonal evolution. By combining these estimates, we quantified the inconsistency of SNVs in each species as a function of time (Fig 4A).

An illustrative example is again provided by *B*. *vulgatus*. At the highest divergence values, we observe little phylogenetic inconsistency for this species (Fig 4A), consistent with the strong population structure suggested by Fig 2B and previous subspecies analyses [36]. For intermediate values of divergence, in contrast, we find that a large majority of all SNVs are inconsistent with the genome-wide divergence estimates. Similarly high values of inconsistency are observed in most of the other species as well (Fig 4A).

While these signals are suggestive of recombination, phylogenetic inconsistencies can also arise from purely clonal mechanisms (e.g., recurrent mutation), or from statistical uncertainties in the genome-wide tree. We therefore sought additional evidence of recombination by examining how phylogenetic inconsistency varies for pairs of SNVs in different locations in the genome. We quantified phylogenetic inconsistency between pairs of SNVs using a standard measure of linkage disequilibrium (LD), defined by the ratio of averages $\sigma_{d}^{2}=E[(f_{AB}-f_{A}f_{B}{)}^{2}]/E[f_{A}(1-f_{A})f_{B}(1-f_{B})]$, with an unbiased estimator to control for varying sample size (S1F Text). The overall magnitude of $\sigma_{d}^{2}$ is not directly informative of recombination, because it also depends on demographic factors, the extent of recurrent mutation, etc. However, if the relative values of $\sigma_{d}^{2}$ consistently decrease for SNVs that are separated by greater genomic distances, then we can conclude that recombination, rather than recurrent mutation, is responsible for the phylogenetic inconsistency that we observe [60].

With traditional metagenomic approaches, it is difficult to measure LD between SNVs unless they co-occur on the same sequencing read. By focusing on QP samples, we can now estimate $\sigma_{d}^{2}$ between SNVs that are separated by greater distances along the reference genome. However, because the synteny of individual lineages may differ substantially from the reference genome, we only assigned coordinate distances (l) to pairs of SNVs in the same gene, which are more likely to be nearby in the genomes in other samples; all other pairs of SNVs are grouped together in a single category ("core-genome-wide"). We then estimated $\sigma_{d}^{2}$ as a function of l for each of these distance categories (S1F Text) and analyzed the shape of this function.

As an example, the inset of Fig 4B illustrates the estimated values of $\sigma^{2}(l)$ for synonymous SNVs in the core genome of *B*. *vulgatus*; similar curves are shown for several other species in S15 Fig. As anticipated by our analysis in Fig 4A, it is crucial to account for the presence of strong population structure. LD among all samples decays only slightly with l, as expected from a mixture of genetically isolated subpopulations. However, if we restrict our attention to the lineages in the largest subpopulation, we observe a pronounced decay in LD. To account for these confounding effects, we manually annotated top-level clades for each species using the genome-wide divergence distribution (S1E Text, part ii) using standard criteria for identifying ecotype clusters [36, 61, 62].

In Fig 4B, we plot summarized versions of the $\sigma^{2}(l)$ curves across a panel of about 40 prevalent species. In almost all cases, we find that core-genome-wide LD is significantly lower than for pairs of SNVs in the same core gene, suggesting that much of the phylogenetic inconsistency in Fig 2 is caused by recombination. Qualitatively similar results are obtained if we repeat our analysis using isolate genomes from some of the more well-characterized species (S16 Fig, S1G Text). In principle, signatures of recombination between genes could be driven by the exchange of intact operons or other large clusters of genes (e.g., on an extra-chromosomal plasmid). However, Fig 4 and S16 Fig also show a significant decay in LD within individual genes, suggesting a role for homologous recombination within genes as well.

The magnitude of the decay of LD within core genes is somewhat less than has been observed in other bacterial species [16] and only rarely decays to genome-wide levels by the end of a typical gene. Moreover, by visualizing the data on a logarithmic scale, we see that the shape of $\sigma_{d}^{2}(l)$ is inconsistent with the predictions of the neutral model (Fig 4A), decaying much more slowly with l than the ∼1/l dependence expected at large distances [63]. Thus, while we can obtain rough estimates of *r*/*μ* by fitting the data to a neutral model (which generally support 0.1≲*r*/*μ*≲10, see S17 Fig), these estimates should be regarded with caution because they vary depending on the length scale on which they are measured (S1F Text). This suggests that new theoretical models will be required to fully understand the patterns of recombination that we observe.

### Short-term succession within hosts

So far, we have focused on evolutionary changes that accumulate over many host colonization cycles. In principle, evolutionary changes can also accumulate within hosts over time. Longitudinal studies have shown that strains and metagenomes sampled from the same host are more similar to each other on average than to samples from different hosts [31, 33, 35, 44, 64, 65]. This suggests that resident populations of bacteria persist within hosts for at least a year (approximately 300 to 3,000 generations), which is potentially enough time for evolutionary adaptation to occur [7]. However, the limited resolution of previous polymorphism- [31] or consensus-based comparisons [35, 44] has made it difficult to quantify the individual changes that accumulate within hosts and to interpret these changes in an evolutionary context.

#### Within-host dynamics reflect a mixture of replacement and modification

To address this issue, we focused on the species in longitudinally sampled HMP subjects that were QP at consecutive time points. This yields a total of 801 resident populations (host × species × time point pairs) across 45 of the most prevalent species (S8 Fig). Our calculations show that the false positives caused by sampling noise should be sufficiently rare that we can resolve a single nucleotide difference between two of these time points in a genome-wide scan (S1C Text, part iv). In contrast to existing reference-based approaches, we have also imposed additional filters to minimize false positives from mapping artifacts (S1A Text).

**Fig 5:**
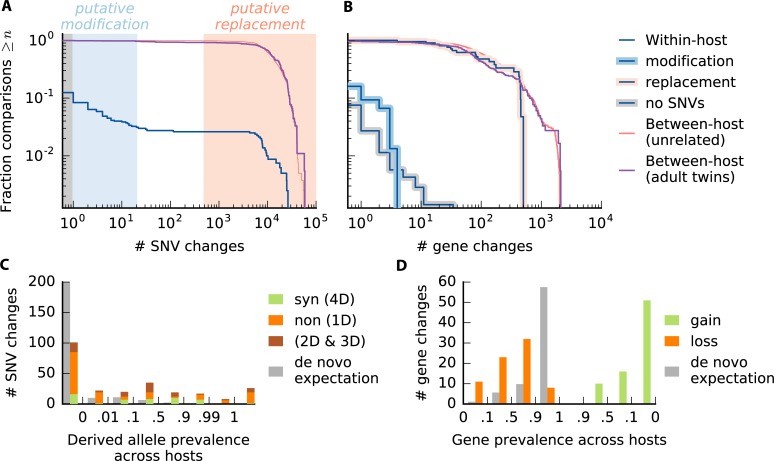
Within-host changes across prevalent species of gut bacteria. (a) Within-host nucleotide differences over 6-month timescales. The blue line shows the distribution of the number of single nucleotide variant (SNV) differences between consecutive quasi-phaseable (QP) time points for different combinations of species, host, and nonoverlapping time interval (if more than two samples are available) for the 45 prevalent species in S20 Fig. The distribution of the number of sites tested in each comparison is shown in S18 Fig. For comparison, the red line shows a matched distribution of the number of SNV differences between each initial time point and a randomly selected Human Microbiome Project host, and the purple line shows the distribution of the number of SNV differences between QP lineages in pairs of adult twins. The shaded regions indicate replacement events (light red, 3% of all within-host comparisons), modification events (light blue, 9% of within-host comparisons), and no detected changes (gray, 88% of within-host comparisons); these ad hoc thresholds were chosen to be conservative in calling modifications. (B) Within-host gene content differences (gains + losses). The blue lines show the distribution of the number of gene content differences within hosts for the samples in (A), with the putative modifications highlighted in light blue, the putative replacements highlighted in light red, and the samples with no SNV changes highlighted in gray. The distribution of the number of genes tested in each comparison is shown in S18 Fig. For comparison, the corresponding between-host and twin distributions are shown as in (A). (C) The total number of nucleotide differences at nondegenerate nonsynonymous sites (1D), 4-fold degenerate synonymous sites (4D), and other sites (2D and 3D) aggregated across the modification events in (A). Sites are stratified based on their prevalence across hosts (S1H Text). For comparison, the gray bars indicate the expected distribution for random de novo mutations (S1H text, part i). (D) The total number of gene loss and gain events among the gene content differences in (B), stratified by the prevalence of the gene across hosts. The de novo expectation for gene losses is computed as in (C); by definition, there are no de novo gene gains.

We first examined the SNV differences that accumulated within each resident population over time. We considered SNVs in both core and accessory genes on the reference genome, because the latter are plausibly enriched for host-specific targets of selection [66]. Consistent with previous work [31, 44], the average number of within-host differences is about 100-fold smaller than the average number of differences between unrelated hosts (S19 Fig). However, the within-host changes are distributed across the different resident populations in a highly skewed manner (Fig 5 and S20 Fig). Visualized on a logarithmic scale, the data reveal a striking multimodal pattern, suggesting that the within-host differences arise from two separate processes.

Most of the resident populations did not have any detectable SNV differences over the roughly 6-month sampling window (i.e., the median is zero). Yet, in a small minority of cases (3%), the resident populations accumulated several thousand mutations, comparable to the typical number of differences between hosts (Fig 5A). This is consistent with previous notions of strain replacement [35], in which the dominant resident strain is succeeded by an effectively unrelated strain from the larger metapopulation. This operational definition includes both the invasion of a new strain (e.g., from other hosts or body sites) or a sudden rise in frequency of a previously colonized strain that had been segregating at low frequency.

In addition to rare replacement events, a larger fraction of resident populations in Fig 5A (about 10% of the total) have a moderate number of SNV differences (on the order of 20 or fewer). We will refer to these as modification events, in order to distinguish them from the replacement events above. In contrast to replacements, modifications preserve most of the genetic information in a lineage when a new genetic change is added. This is true at the level of nucleotide divergence but also for gene content (Fig 5B) and the sharing of private marker SNVs (S11 Fig). We therefore hypothesize that the modification events in Fig 5 reflect heritable evolutionary changes that have risen to high frequency within the host.

The fact that these O(1) frequency changes occur within 6-month timescales already provides some information about their possible evolutionary causes. For example, if the frequency changes were caused by genetic drift, the effective population size would have to be as small as *N*_*e*_~200*λ*, where *λ* is the number of generations that take place per day (*λ*≲20). These numbers are difficult to reconcile with the large census sizes of many gut bacteria (*N*≳10^9^ [67, 68]) unless extreme population bottlenecks have occurred. On the other hand, if the frequency changes were caused by natural selection, then the corresponding fitness benefits must be at least *S*~1% per day. Even in this case, however, the observed SNVs may not be the direct targets of selection themselves: given the limitations of our reference-based approach, and our aggressive filtering scheme, the observed mutations may simply be passengers that are linked to an unseen selected locus.

To further probe the dynamics of within-host evolution, we therefore pooled the 248 SNV differences observed across the 75 modification events in our cohort, and we stratified them according to two additional criteria. We first partitioned the SNVs according to how prevalent the sweeping allele was among the other hosts in our cohort (Fig 5C and S21 Fig). By comparing this distribution against the null expectation for randomly selected sites, we find that there are significantly more intermediate- and high-prevalence mutations than expected for random de novo mutations (*P*<10^−4^, S1H Text, part i). One potential explanation for this signal could be parallel evolution [69], e.g., if the same strongly beneficial mutations independently arose and fixed in different hosts. However, we can rule out this recurrent sweep hypothesis by further partitioning the SNVs into synonymous and nonsynonymous mutations (Fig 5C). The relative fractions of the two types are distributed across the different prevalence classes in a highly nonuniform manner (*P*<10^−4^, S1H text, part ii). Among rare alleles (<1% prevalence), we observe an excess of nonsynonymous mutations [*d*_*N*_/*d*_*S*_≈1.3 (0.8,2.4)], consistent with positive selection and/or hitchhiking. By contrast, nonsynonymous mutations are depleted and synonymous mutations enriched for alleles with intermediate prevalence (0.1<*f*<0.9), precisely where the recurrent sweep hypothesis requires the strongest selection pressures. These low values (*d*_*N*_/*d*_*S*_≈0.1) are surprising even for pure passenger mutations, because purifying selection should be rendered inefficient over these short timescales [70], similar to what we observed in Fig 3.

Together, these observations suggest an alternate hypothesis, in which some of the within-host sweeps are driven by much older DNA fragments that were acquired through recombination. This could explain the intermediate prevalence of some sweeping alleles, because standing variants can arrive through recombination. It can also explain their low *d*_*N*_/*d*_*S*_ values, because there is more time for deleterious mutations to be purged (and for synonymous mutations to accumulate) before the fragment is transferred.

Consistent with this hypothesis, we also found evidence for a small number of gene content differences between the two time points in many of the non-replacement samples (Fig 5B). Gene content differences were twice as likely to occur in populations in which we observed one or more SNV differences (*P*≈0.025, Fisher exact test), although the overall rates are still modest under our current filtering criteria (about 10%). We observed a roughly equal contribution from gains and losses (Fig 5D). The gene losses could be consistent with simple clonal processes (e.g., a large deletion mutation) as well as recombination (e.g., if the incorporated homologous fragment lacks the gene in question). Gene gains, on the other hand, must either reflect a recombination event or a more complex sweep involving the sudden decline of a previously successful deletion. The genes that are gained and lost tend to be drawn from the accessory portion of the genome (Fig 5D and S21 Fig), consistent with the expectation that these genes are more likely to be gained or lost over time.

#### Replacement dominates over longer within-host timescales

The successional dynamics in the HMP cohort raise a number of questions about how these dynamics play out over longer timescales. For example, does the probability of a replacement accumulate uniformly with time, so that we would expect most strains to be replaced after 20 years? Or are replacements concentrated in a few replacement-prone individuals, with a negligible rate among the larger population? Alternatively, do resident populations eventually acquire enough evolutionary changes that they become so well adapted the host that replacements become less likely to succeed?

To fully address these questions, we would require a large longitudinal cohort with metagenomes collected over a period of decades. However, we can approximate this design in a crude way by comparing metagenomes collected from a cohort of about 200 adult twins from the TwinsUK project [45]. Comparisons of younger twins suggest that they may be colonized by nearly identical strains in childhood [46] (S22 Fig). By comparing QP samples in adult twins, we can therefore gain insight into the changes that have occurred in the 20–40 years that the hosts have spent in separate households.

The numbers of SNV and gene changes between the resident populations in each twin pair are illustrated in Fig 5A and 5B. We observe striking departures from the within-host distribution: while 3% of the resident populations experienced a replacement event on 6-month timescales in the HMP study, more than 90% of the resident populations in twins have more than 1,000 SNV differences between them. Compared with the modification events we observed in the HMP study, these highly diverged twin strains have much lower rates of private marker SNV sharing (S11 Fig), along with a higher proportion of SNVs with intermediate prevalence (S23 Fig). Together, these lines of evidence suggest that the highly diverged strains in twins are true replacement events, rather than an accumulation of many evolutionary changes. The 16 resident populations with fewer than 1,000 SNV differences were scattered across 13 twin pairs. All had at least one SNV or gene difference between the twins (median 29 SNVs and 1.5 genes), which is significantly higher than the within-host distribution from the HMP cohort. However, a larger sample size is required to determine what fraction of these SNVs accumulated since colonization.

Together, these data suggest that a vast majority of the resident populations have experienced a replacement over the 20–40 years that their hosts have spent in different households. This observation is consistent with a straightforward extrapolation of the short-term estimates from the HMP cohort, which predicts that replacement should dominate over modification in a typical population after about 20 years. In other words, replacement is not confined to a few special hosts but will eventually occur for most (Western) individuals given enough time. This suggests that the potential benefits of local adaptation do not compound indefinitely.

The high prevalence of twin replacements also provides insight into the two replacement mechanisms described in the previous section. If replacements are primarily drawn from a set of strains that colonized both twins during childhood, then the replacement probability should saturate at 1−1/*n*_*c*_, where *n*_*c*_ is the number of colonizing strains. The observed replacement probability of 90% would then imply that the number of low-frequency colonizing strains for each species must be as large as *n*_*c*_~10, or that most of the replacements are caused by the invasion of new strains that arrive after initial colonization. It will be interesting to test these alternative mechanisms with deeper sequencing and longer time courses.

## Discussion

Evolutionary processes can play an important role in many microbial communities. Yet, despite increasing amounts of sequence data, our understanding of these processes is often limited by our ability to resolve evolutionary changes in populations from complex communities. In this work, we quantify the evolutionary forces that operate within bacteria in the human gut microbiome by characterizing in detail the lineage structure of approximately 40 species in metagenomic samples from individual hosts.

Building on previous work [35] and others, we found that many resident populations from a variety of prevalent species are best described by an "oligo-colonization" model, in which a few distinct strains from the larger population are present at intermediate frequencies, with the identities and frequencies of these strains varying from person to person (Fig 1). The distribution of strain frequencies in this oligo-colonization model is itself quite interesting: in the absence of fine tuning, it is not clear what mechanisms would allow for a second or third strain to reach intermediate frequency, while preventing a large number of other lineages from entering and growing to detectable levels at the same time. A better understanding of the colonization process and how it might vary among the species in Fig 1F is an important avenue for future work.

Given the wide variation among species and hosts, we chose to focus on a subset of samples with particularly simple strain mixtures for a given species, in which we can resolve evolutionary changes in the dominant lineage with a high degree of confidence. Our quasi-phasing approach can be viewed as a refinement of the consensus approximation employed in earlier studies [4, 35, 37, 38] but with more quantitative estimates of the errors associated with detecting genetic differences between lineages in different samples.

By analyzing genetic differences between lineages in separate hosts, we found that long-term evolutionary dynamics in many gut bacteria are consistent with quasi-sexual evolution and purifying selection, with relatively weak geographic structure. Earlier work had documented extensive horizontal transfer between distantly related species in the gut [71, 72], but our ability to estimate rates of recombination within species was previously limited by the small number of sequenced isolates for many species of gut bacteria [73]. The high rates of homologous recombination we observed with our quasi-phasing approach are qualitatively consistent with previous observations in other bacterial species [16, 73–77]; although the rates of recombination are high relative to the typical divergence time, we note that they may still allow for genome-wide sweeps or divergence between nascent ecotypes given sufficiently strong selection pressures. Beyond the overall rates, our quantitative characterization of LD also revealed interesting departures from the standard neutral prediction that cannot be captured by any choice of recombination rate. Understanding the origin of this discrepancy is an interesting topic for future work. It is also interesting to ask how these long-term rates of recombination could emerge from the oligo-colonization model above, because it would seem to limit opportunities for genetic exchange among strains of the same species.

In a complex community like the gut, a key advantage of our metagenomic approach is that it can jointly measure genetic differences in multiple species for the same pair of hosts. By leveraging this feature, we found that previous observations of highly similar strains in different hosts [35, 44] are not driven by cryptic host relatedness. Instead, the presence of these closely related strains and the genetic differences that accumulate between them may be driven by more general population genetic processes in bacteria that operate on timescales much shorter than the typical coalescent time across hosts. It is difficult to produce such closely related strains in traditional population genetic models of loosely linked loci [78] (or "bags of genes" [79]), although recent hybrid models of vertical and horizontal inheritance [77, 80] or fine-scale ecotype structure [62] could potentially provide an explanation for this effect. Further characterization of these short-term evolutionary processes will be vital for current efforts to quantify strain sharing across hosts [33, 46, 56], which often require implicit assumptions about how genetic changes accumulate on short timescales. Our results suggest that these short-term dynamics of across-host evolution may not be easily extrapolated by comparing average pairs of strains.

The other main advantage of our quasi-phasing approach is its ability to resolve a small number of evolutionary changes that could accumulate within hosts over short timescales. Previous work has shown that on average, longitudinally sampled metagenomes from the same host are more similar to each other than metagenomes from different hosts [31, 33, 64, 65], and that some within-host changes can be ascribed to replacement by distantly related strains [35, 44]. However, the limited resolution of previous polymorphism- [31] or consensus-based comparisons [35, 44] had made it difficult to determine whether resident strains also evolve over time.

Our quasi-phasing approach overcomes this limitation, enabling finely resolved estimates of temporal change within individual species in individual hosts. This increased resolution revealed an additional category of within-host variation, which we have termed modification, in which resident strains acquire modest numbers of SNV and gene changes over time. This broad range of outcomes shows why it is essential to understand the distribution of temporal variation across hosts: even though modification events were about 3 times more common than replacements in our cohort, their contributions to the total genetic differences are quickly diluted as soon as a single replacement is included (S19 Fig). As a result, we expect that previous metagenome-wide [31] or species-averaged [44] estimates of longitudinal variation largely reflect the rates and genetic differences associated with replacement events, rather than evolutionary changes.

Although we have interpreted modifications as evolutionary events (i.e., mutations to an existing genome), it is possible that they could also reflect replacement by extremely closely related strains, as in Fig 2. The present data seem to argue against this scenario: modifications are not only associated with different patterns of SNV sharing (S11 Fig), but we also observe significant asymmetries in the prevalence distributions in Fig 5C and 5D that depend on the temporal ordering of the 2 samples (see Fig 5). This temporal directionality arises naturally in certain evolutionary models (e.g., the de novo mutation model in Fig 5C), but it is less likely to emerge from steady-state competition between a fixed set of strains. Unambiguous proof of evolution could also be observed in a longer time course, because subsequent evolutionary changes should eventually accumulate in the background of earlier substitutions. Further investigation of these nested substitutions remains an interesting topic for future work.

The signatures of the sweeping SNVs, along with the presence of gene gain events, suggest that some of the within-host sweeps we observed were seeded by recombination, rather than de novo mutation. In particular, many of the alleles that swept within hosts were also present in many other hosts, yet their *d*_*N*_/*d*_*S*_ values indicated strong purifying selection, consistent with an ancient polymorphism (Fig 3). Sweeps of private SNVs, by contrast, were associated with a much higher fraction of nonsynonymous mutations, consistent with adaptive de novo evolution. Interestingly, we also observe a slight excess of private nonsynonymous mutations between closely related strains in different hosts (S12 Fig). This suggests that some of the differences observed between hosts may reflect a record of recent within-host adaptation.

Recombination-seeded sweeps would stand in contrast to the de novo mutations observed in microbial evolution experiments [13] and some within-host pathogens [21, 22]. Yet in hindsight, it is easy to see why recombination could be a more efficient route to adaptation in a complex ecosystem like the gut microbiome, given the large strain diversity [42], the high rates of DNA exchange [71, 72], and the potentially larger selective advantage of importing an existing functional unit that has already been optimized by natural selection [11]. Consistent with this hypothesis, adaptive introgression events have also been observed on slightly longer timescales in bacterial biofilms from an acid mine drainage system [14], and they are an important force in the evolution of virulence and antibiotic resistance in clinical settings [81].

While the data suggest that some within-host changes may be seeded by a recombination event, it is less clear whether ongoing recombination is relevant during the sweep itself. Given the short timescales involved, we would expect many of the observed sweeps to proceed in an essentially clonal fashion, because recombination would have little time to break up a megabase-sized genome given the typical rates inferred in S17 Fig. If this were the case, it would provide many opportunities for substantially deleterious mutations (with fitness costs of order *S*_*d*_~1% per day) to hitchhike to high frequencies within hosts [70], thereby limiting the ability of bacteria to optimize to their local environment. The typical fitness costs inferred from Fig 2D lie far below this threshold and would therefore be difficult to purge within individual hosts. In this scenario, the low values of *d*_*N*_/*d*_*S*_ observed between hosts (as well as the putative introgression events) would crucially rely on the competition process across hosts [82]. Although the baseline recombination rates suggest clonal sweeps, there are also other vectors of exchange (e.g., transposons, prophage, etc.) with much higher rates of recombination. Such mechanisms could allow within-host sweeps to behave in a quasi-sexual fashion, preserving genetic diversity elsewhere in the genome. These sweeps of local genomic regions are predicted in certain theoretical models [83, 84] and have been observed in a few other bacterial systems [15, 17, 85]. If sweeps of local genomic regions were also a common mode of adaptation in the gut microbiome, they would allow bacteria to purge deleterious mutations more efficiently than in the clonal scenario above.

Although evolution was more common than replacement on 6-month timescales, our analysis of adult twins suggests that the rare replacement events eventually dominate on multi-decade timescales. This suggests that resident strains are limited in their ability to evolve to become hyper-adapted to their host, because most strains were eventually susceptible to replacement. Such behavior would be consistent with theoretical models in which strains of the same species only partially overlap in their ecological niches [27, 54]. Although our results indicate that the long-term probability of replacement is largely uniform across hosts, it remains an open question whether these events occur more or less uniformly in time or whether they occur in punctuated bursts during major ecosystem perturbations (e.g., antibiotic treatment). This would be an interesting question to address with denser and longer time series data.

Finally, while we have identified many interesting signatures of within-host adaptation, there are several important limitations to our analysis. One class concerns the events that we cannot observe with our approach (i.e., false negatives). These are particularly relevant here, because we have discarded substantial amounts of data in an attempt to overcome the traditional problems of metagenomic inference (S24 Fig). For example, our reference-based method only tracks SNVs and gene copy numbers in the genomes of previously sequenced isolates of a given species. Within this subset, we have also imposed a number of stringent bioinformatic filters, further limiting the sequence space that we consider. Thus, it is likely that we are missing many of the true targets of selection, which might be expected to be concentrated in the host-specific portion of the microbiome, multi-copy gene families, or in genes that are shared across multiple prevalent species. A further limitation is that we can only analyze the evolutionary dynamics of QP samples (although the consistency of our results for species with different QP fractions suggests that this might not be a major issue). Finally, a potentially more important false negative is that our current method can only identify complete or nearly complete sweeps within individual hosts. While we observed many within-host changes that matched this criterion, we may be missing many other examples of within-host adaptation in which variants do not completely fix. Given the large population sizes involved, such sweeps can naturally arise from phenotypically identical mutations at multiple genetic loci [69, 86], or through additional ecotype partitioning between the lineages of a given species [23, 25]. Both mechanisms have been observed in experimental populations of *Escherichia coli* adapting to a model mouse microbiome [7].

In addition to these false negatives, the other limitation of our approach concerns potential false positives inherent in any metagenomic analysis. With short-read data, it is difficult to truly know whether a paticular DNA fragment is linked to a particular species or whether it resides in the genome of another species (perhaps an uncultured one) that is fluctuating in abundance. False SNV and gene changes can therefore occur because of these read donating effects. The temporally asymmetric prevalence distributions in Fig 5C and 5D suggest that our filters were successful in eliminating many of these events (S1H Text, part iii). However, isolate or long-read sequences are required to unambiguously prove that these variants are linked to the population of interest.

Fortunately, two concurrent studies have also documented short-term evolution of gut bacteria within healthy human hosts using an isolate-based approach [87, 88]. Each study focused on a single bacterial species, *E*. *coli* in [87] and *B*. *fragilis* in [88]. Although *E*. *coli* was not sufficiently abundant in our cohort to be included in our within-host analysis, the observations in *B*. *fragilis* are largely consistent with our findings that within-host evolution can be rapid and that it can be mediated by recombination in addition to new mutations. Crucially, because these observations were obtained using an isolate-based approach, they are not subject to the same methodological limitations described above, and they therefore serve as an independent verification of our results. However, because our statistical approach provides simultaneous observations across more than 40 prevalent species, our results show that these general patterns of within-host evolution are shared across many species of gut bacteria, and they demonstrate a general approach for investigating these forces in widely available metagenomic data. Future efforts to combine metagenomic- and isolate-based approaches, e.g., by incorporating long-range linkage information [41, 89, 90], will be crucial for building a more detailed understanding of these evolutionary processes.

## Supporting information

S1 Fig Example within-host allele frequency distributions for 24 additional species (1/2). Analogous versions of Fig 1A–1D for 24 additional species from Fig 1F. For each species, 6 randomly chosen non–quasi-phaseable samples are plotted.
(PDF)

S2 Fig Example within-host allele frequency distributions for 24 additional species (2/2). This figure is a continuation of S1 Fig.
(PDF)

S3 Fig Rates of within-host polymorphism for 24 additional species. Analogous versions of Fig 1E for the 24 species in S1 Fig and S2 Fig.
(PDF)

S4 FigSchematic depiction of phasing and substitution errors.(a) An example of a haplotype phasing error, in which an allele with true within-host frequency *f* [drawn from a hypothetical genome-wide prior distribution, *p*_0_(*f*), blue] is observed with a sample frequency $\hat{f}$, with the opposite polarization. (b) An example of a falsely detected nucleotide substitution between 2 samples, in which an allele with true frequency *f*_1_ = *f*_2_ = *f* [drawn from a hypothetical genome-wide null distribution, *p*_0_(*f*), blue] is observed with a sample frequency ${\hat{f}}_{1}<20\%$ in one sample and ${\hat{f}}_{2}>80\%$ in another. Allele frequency pairs that fall in the pink region are counted as nucleotide differences between the 2 samples, while pairs in the gray shaded region are counted as evidence for no nucleotide difference; all other values are treated as missing data.(PDF)

S5 Fig Average genetic distance between *B*. *vulgatus* metagenomes. (a) The fraction of 4-fold degenerate synonymous sites in the core genome that have major allele frequencies ≥80% and differ in a randomly selected sample (see S1C Text for a formal definition). (b) The corresponding rate of intermediate-frequency polymorphism for each sample, reproduced from Fig 1B. In both panels, samples are plotted in the same order as in Fig 1B.
(PDF)

S6 Fig Correlation between within-host diversity and the fraction of non–quasi-phaseable (QP) samples per species. Circles denote the average rate of within-host polymorphism (as defined in Fig 1E) for each species as a function of the fraction of non-QP samples in that species.
(PDF)

S7 Fig Distribution of the number of quasi-phaseable (QP) species per sample. Left: the distribution of the fraction of QP species per sample (blue line). The gray line denotes the corresponding null distribution obtained by randomly permuting the QP classifications across the samples. We conclude that QP species are not strongly enriched within specific hosts. Right: the number of species classified as QP in each sample on the left as a function of the number of species with sufficient coverage in that sample. A small amount of noise is added to both axes to enhance visibility.
(PDF)

S8 Fig Distribution of quasi-phaseable (QP) samples in longitudinal samples and adult twin pairs. Bars show the number of sample pairs for each species that are QP for both samples (QP→QP), non-QP for both samples (non→non), mixed samples (QP→non or non→QP), and pairs in which the species did not have sufficient coverage in one of the two time points (dropout). The left panel shows data from longitudinally sampled individuals in the Human Microbiome Project cohort [42, 44], while the right panel compares contemporary samples from pairs of adult twins [45]. Species are ordered in decreasing order of prevalence in the HMP cohort. Species are only included if they have at least 10 QP samples and at least 3 QP time point pairs.
(PDF)

S9 Fig Distribution of estimated gene copy numbers for the 4 samples in Fig 1. The gray region denotes the copy number range required in at least one sample to detect a difference in gene content between a pair of samples (see S1C Text, part v).
(PDF)

S10 Fig Single nucleotide variant (SNV) and gene content differences between closely related strains. (a) Cumulative distribution of the total number of core genome SNV differences between closely related strains in Fig 2. (b) Cumulative distribution of the number of gene content differences for the closely related strains in panel a (red line). For comparison, the corresponding distribution for all pairs of strains in Fig 2 is shown in black, while the gray line denotes a “clocklike” null distribution for the closely related strains, which assumes that genes and SNVs each accumulate at constant rates.
(PDF)

S11 Fig Private marker single nucleotide variant (SNV) sharing within and between hosts. Given an ordered pair of quasi-phaseable strains, we define private marker SNVs to be core genome SNVs that (i) are phaseable in both strains, (ii) have the derived allele in strain 1, and (iii) do not have the derived allele in any other host outside the pair. The marker sharing fraction *p* is then defined as the fraction of private marker SNVs that also have the derived allele in strain 2. (a) Private marker SNV sharing between unrelated hosts. Solid lines show the distribution of marker sharing fraction *p* between all pairs of strains in Fig 2 (black) and between the subset of closely related strains (red). Separate sharing fractions are calculated for both orderings of a given strain pair, and we only include pairs with at least 10 marker SNVs. (b) Distribution of marker SNV sharing for replacement and modification events in longitudinally sampled Human Microbiome Project hosts (blue lines), using the replacement and modification thresholds in Fig 5A. For comparison, the distribution of marker SNV sharing between strains in pairs of adult twins is shown in purple. For twins, we use modified definitions of replacement (>10^3^ SNV differences) and modification (<10^3^ SNV differences). As above, sharing fractions are only computed for samples with at least 10 marker SNVs.
(PDF)

S12 FigSignatures of selective constraint within private single nucleotide variants (SNVs).An analogous version of Fig 3 computed for private SNVs. For each quasi-phaseable (QP) species × host combination, *d*_*N*_/*d*_*S*_ is computed for the subset of alleles that are not found in any other hosts. These private *d*_*N*_/*d*_*S*_ ratios are plotted as a function of $d_{S}^{*}$, an estimate of the minimum synonymous divergence from other QP lineages of that species. The inset shows the ratio between the cumulative *d*_*N*_ and *d*_*S*_ values for all lineages with $d_{S}^{*}$ less than the indicated value. The narrow shaded region denotes 95% confidence intervals estimated by Poisson resampling. The resampling procedure uses an analogous version of the thinning scheme employed in Fig 3 to ensure that the *x* and *y* axes are statistically independent (see S1D Text).(PDF)

S13 FigSchematic illustration of phylogenetic inconsistency between individual single nucleotide variants (SNVs) and core-genome-wide divergence.Two examples are shown, illustrating phylogenetically consistent and inconsistent SNVs, respectively, in a sample of 4 lineages. The lineages at the leaves of each tree are labeled according to whether they have the major (*M*) or minor (*m*) allele. Thunderbolts depict the most parsimonious introduction of the derived allele on the genealogy. Different colors indicate the core-genome-wide divergence between lineages with different combinations of alleles, as described in S1E Text, part i. Highlighted in purple is *d*_*B*_, which is the minimum divergence between two lineages bearing different alleles. Highlighted in red and green are $d_{W}^{M}$ and $d_{W}^{m}$, which are the maximum divergence between individuals bearing the same allele (major and minor, respectively). In practice, we do not know which allele is ancestral and which is derived, so we define $d_{W}=\min(d_{W}^{m},d_{W}^{M})$. If *d*_*W*_≫*d*_*B*_, we say that the SNV is phylogenetically inconsistent.(PDF)

S14 Fig Top-level clade structure among lineages in different quasi-phaseable hosts. Core-genome-wide *F*_*st*_ between manually assigned top-level clades in each species (S2 Table, S1E Text, part ii). Species are only included if there are at least 2 clades with more than 2 individuals in each of them.
(PDF)

S15 Fig Decay of linkage disequilibrium in three example species. Analogous versions of the insets in Fig 4B for *Bacteroides fragilis*, *Parabacteroides distasonis*, and *Alistipes shahii*.
(PDF)

S16 Fig Recapitulating patterns of between-host evolution from sequenced isolates. (a) An analogous version of Fig 2B constructed from the genomes of sequenced isolates in 6 representative species, as described in S1G Text. (b) An analogous version of Fig 3 constructed from the pairs of isolate genomes in panel a. (c-h) Analogous versions of Fig 4B inset for the 6 species in (a).
(PDF)

S17 FigRecombination rate estimates based on the decay of linkage disequilibrium.For each species, the two dashes represent effective values of *r*/*μ* estimated from the neutral prediction for the decay of $\sigma_{d}^{2}(l)$, using the half-maximum and quarter-maximum decay lengths (see S1F Text). The 2 estimates are connected by a vertical line for visualization. The overall rates of recombination are qualitatively consistent with observations in several other bacterial species [16, 73–77].(PDF)

S18 Fig Distribution of the number of sites (a) and genes (b) tested in each of the within-host comparisons in Fig 5.
(PDF)

S19 Fig Average number of single nucleotide variant (SNV) differences within and between hosts. Blue and red bars denote the average of the within- and between-host distributions in Fig 5A. Consistent with previous work [31, 44], the within-host average is about 100-fold lower than the between-host average. However, the average is a poor summary of the typical values in the between-host distribution in Fig 5A. Instead, the within-host average is well approximated by the product of the typical number of SNV differences per replacement and the overall fraction of replacement events.
(PDF)

S20 Fig Comparable rates of within-host single nucleotide variant (SNV) and gene changes across prevalent species. Summary of within-host SNV changes (top) and gene changes (bottom) across all species with at least 10 quasi-phaseable samples and at least 3 pairs of longitudinal QP samples. Each row in each bar represents a different longitudinal pair from the Human Microbiome Project cohort, and rows are colored according to the total number of SNV changes (top) and gene changes (bottom), with gray indicating no detected changes. A star indicates that the total number of non-replacement changes is ≥10 times the total estimated error rate across samples from that species (see S1C Text, part iv, and S1C Text, part v), in which replacements are defined as in Fig 5.
(PDF)

S21 Fig Prevalence distributions of within-host single nucleotide variant and gene content differences without binning. (a) The empirical survival function for the raw prevalence values in Fig 5C. For comparison, the gray line shows the time-reversal symmetric version described in S1H Text, part iii. (b) Empirical prevalence distributions for synonymous (1D) and nonsynonymous (4D) differences in Fig 5C. (c) Empirical prevalence distributions for gene gains and losses in Fig 5D.
(PDF)

S22 Fig Single nucleotide variant and gene content differences between younger twins. Light purple lines denote analogous versions of Fig 5A and 5B for 4 twin pairs from [46], which range from about 5 to about 20 years of age. The results are consistent with the original findings in [46]. For comparison, the dark purple lines reproduce the adult twin distributions from Fig 5A and 5B. These data show that strains from younger twins are significantly more similar to each other than strains from adult twins (*P*<10^−4^, permutation Kolmogorov–Smirnov test [91]).
(PDF)

S23 Fig Prevalence of single nucleotide variant (SNV) and gene content differences between adult twins. Analogous versions of Fig 5C and 5D computed using the SNV and gene content differences observed between all adult twin pairs (purple lines in Fig 5A and 5B). In contrast to the within-host changes in Fig 5C and 5D, the prevalence distributions and the relative fraction of nonsynonymous differences between twins are more consistent with replacement by a distantly related strain.
(PDF)

S24 Fig Schematic figure illustrating the data discarded at various steps in our pipeline. (PDF)

S1 Table Metagenomic samples used in study. We analyzed a total of 1,013 samples from 693 individuals. This included samples from 250 individuals from the Human Microbiome Project (HMP) [42, 44], 250 individuals from [45], 185 individuals from [43], and 8 individuals from [46]. Listed are the subject identifiers, sample identifiers, run accessions, country of the study, continent of the study, visit number, and study (HMP, Xie and colleagues, Korpela and colleagues, or Qin and colleagues, 2012).
(TXT)

S2 Table Top-level clade definitions. This table contains the manually defined top-level clades described in S1E Text, part ii. Rows list the various combinations of species and hosts plotted in Fig 2 along with their corresponding numeric clade label.
(TXT)

S1 Text Methods and supplemental information. (A) Metagenomic pipeline. (B) Quantifying within-species diversity in individual samples. (C) Quasi-phasing metagenomic samples. (D) Population genetic null model of purifying selection for pairwise divergence across hosts. (E) Phylogenetic inconsistency and clade structure across hosts. (F) Population genetic null model for the decay of linkage disequilibrium. (G) Validation of between-host patterns using isolate sequences. (H) Quantifying prevalence of within-host single nucleotide variant and gene changes.
(PDF)

## Abbreviations

HMP: Human Microbiome Project
LD: linkage disequilibrium
QP: quasi-phaseable
SNV: single nucleotide variant
