# Supplementary figures and images for: Evolutionary dynamics of bacteria in the gut microbiome within and across hosts
Source: PLoS Biol. 2019 Jan 23;17(1):e3000102. doi: 10.1371/journal.pbio.3000102 (PMC6361464; doi:10.1371/journal.pbio.3000102)

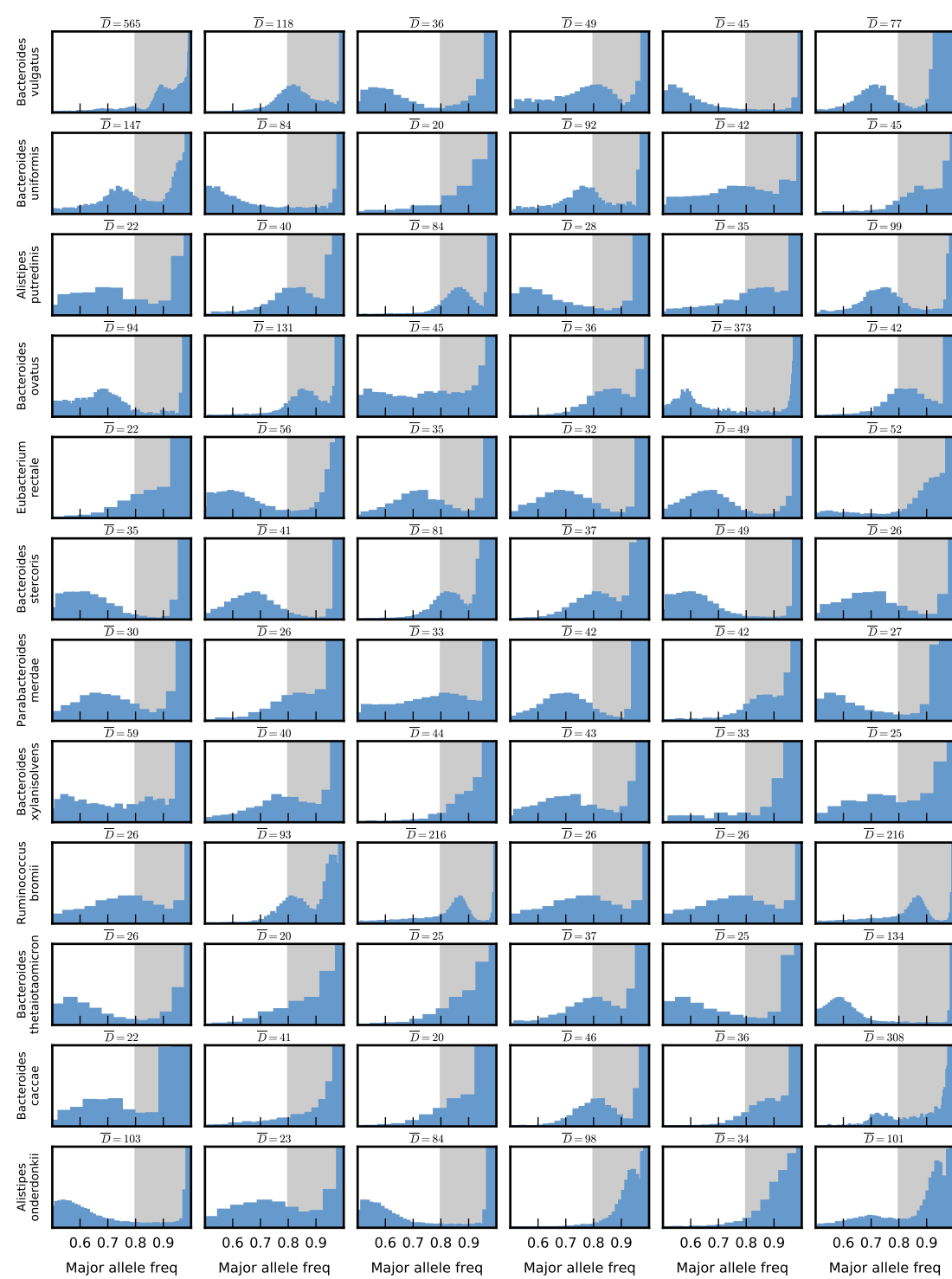

Supplement: S1 Fig — Analogous versions of Fig 1A–1D for 24 additional species from Fig 1F. For each species, 6 randomly chosen non–quasi-phaseable samples are plotted. (PDF) [file pbio.3000102.s001.pdf]

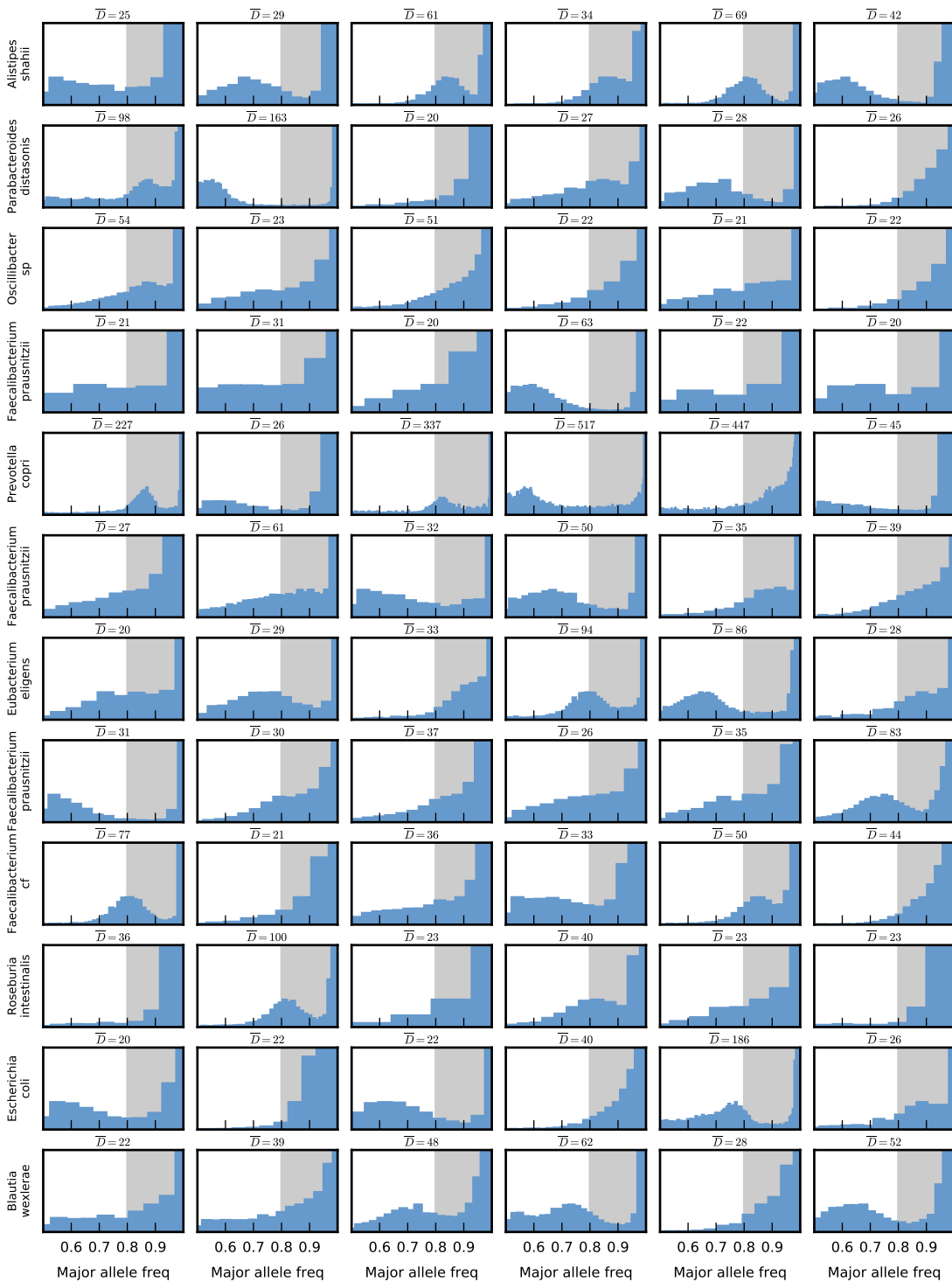

Supplement: S2 Fig — This figure is a continuation of S1 Fig. (PDF) [file pbio.3000102.s002.pdf]

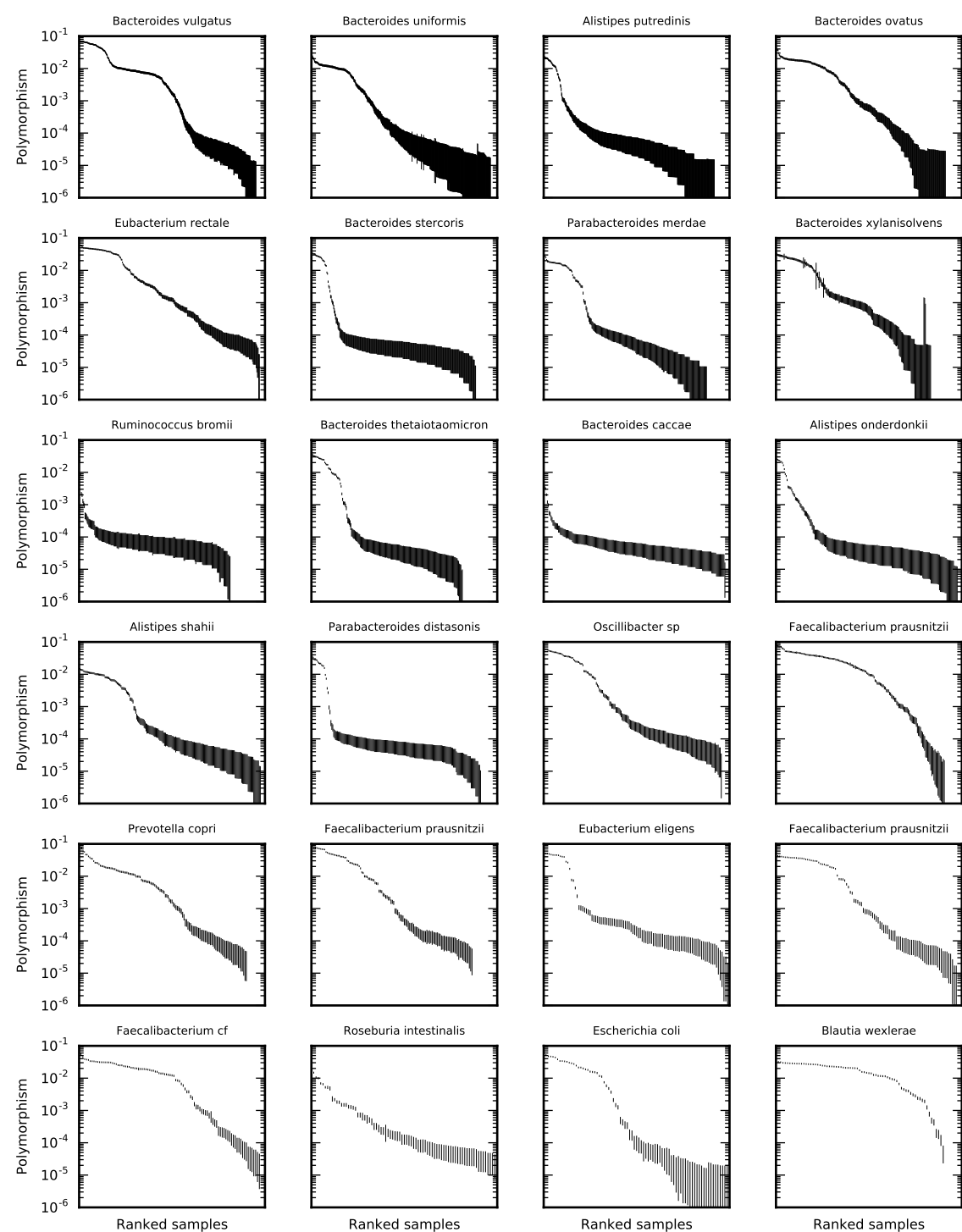

Supplement: S3 Fig — Analogous versions of Fig 1E for the 24 species in S1 Fig and S2 Fig. (PDF) [file pbio.3000102.s003.pdf]

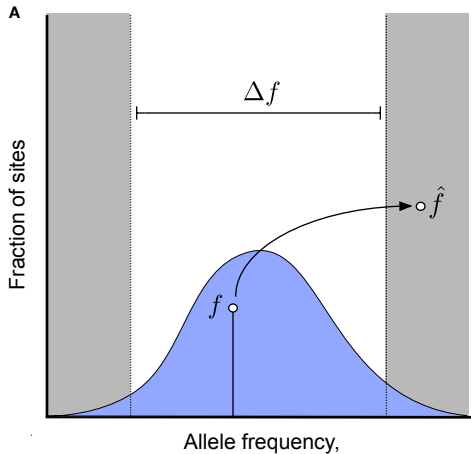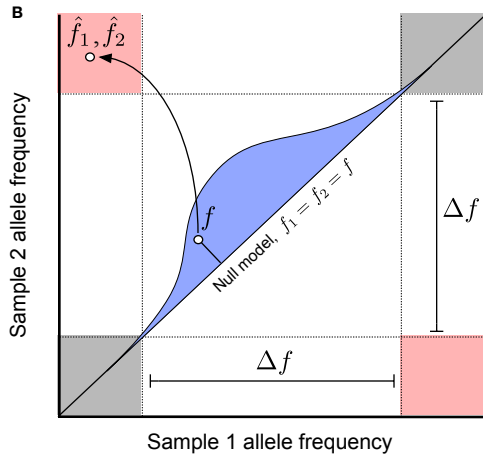

Supplement: S4 Fig — (a) An example of a haplotype phasing error, in which an allele with true within-host frequency f [drawn from a hypothetical genome-wide prior distribution, p0(f), blue] is observed with a sample frequency f^, with the opposite polarization. (b) An example of a falsely detected nucleotide substitution between 2 samples, in which an allele with true frequency f1 = f2 = f [drawn from a hypothetical genome-wide null distribution, p0(f), blue] is observed with a sample frequency f^1<20% in one sample and f^2>80% in another. Allele frequency pairs that fall in the pink region are counted as nucleotide differences between the 2 samples, while pairs in the gray shaded region are counted as evidence for no nucleotide difference; all other values are treated as missing data. (PDF) [file pbio.3000102.s004.pdf]

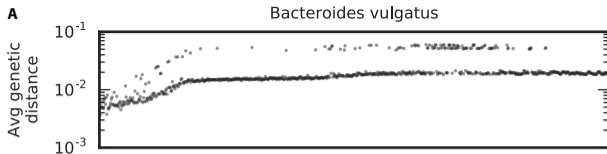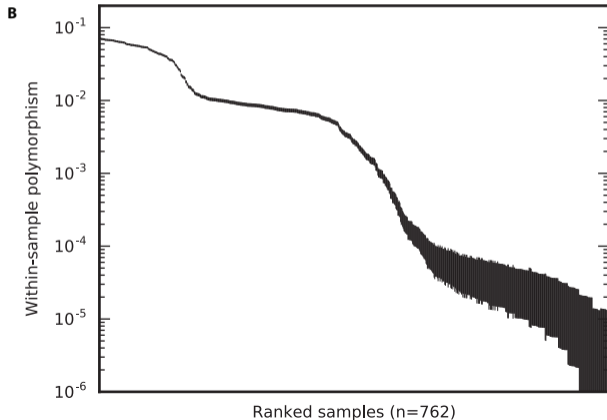

Supplement: S5 Fig — (a) The fraction of 4-fold degenerate synonymous sites in the core genome that have major allele frequencies ≥80% and differ in a randomly selected sample (see S1C Text for a formal definition). (b) The corresponding rate of intermediate-frequency polymorphism for each sample, reproduced from Fig 1B. In both panels, samples are plotted in the same order as in Fig 1B. (PDF) [file pbio.3000102.s005.pdf]

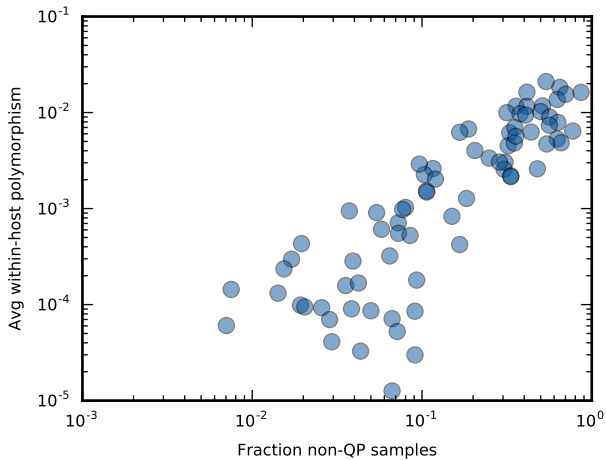

Supplement: S6 Fig — Circles denote the average rate of within-host polymorphism (as defined in Fig 1E) for each species as a function of the fraction of non-QP samples in that species. (PDF) [file pbio.3000102.s006.pdf]

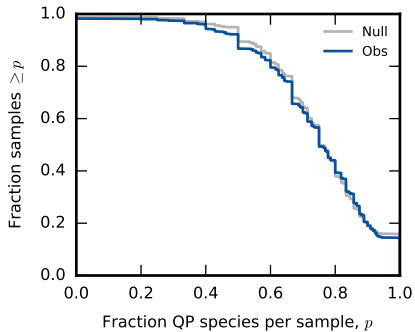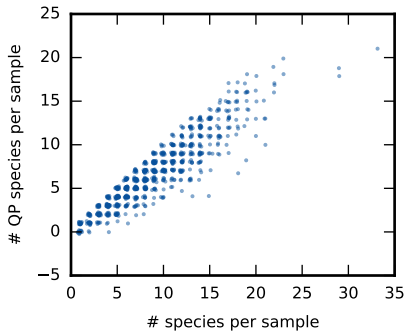

Supplement: S7 Fig — Left: the distribution of the fraction of QP species per sample (blue line). The gray line denotes the corresponding null distribution obtained by randomly permuting the QP classifications across the samples. We conclude that QP species are not strongly enriched within specific hosts. Right: the number of species classified as QP in each sample on the left as a function of the number of species with sufficient coverage in that sample. A small amount of noise is added to both axes to enhance visibility. (PDF) [file pbio.3000102.s007.pdf]

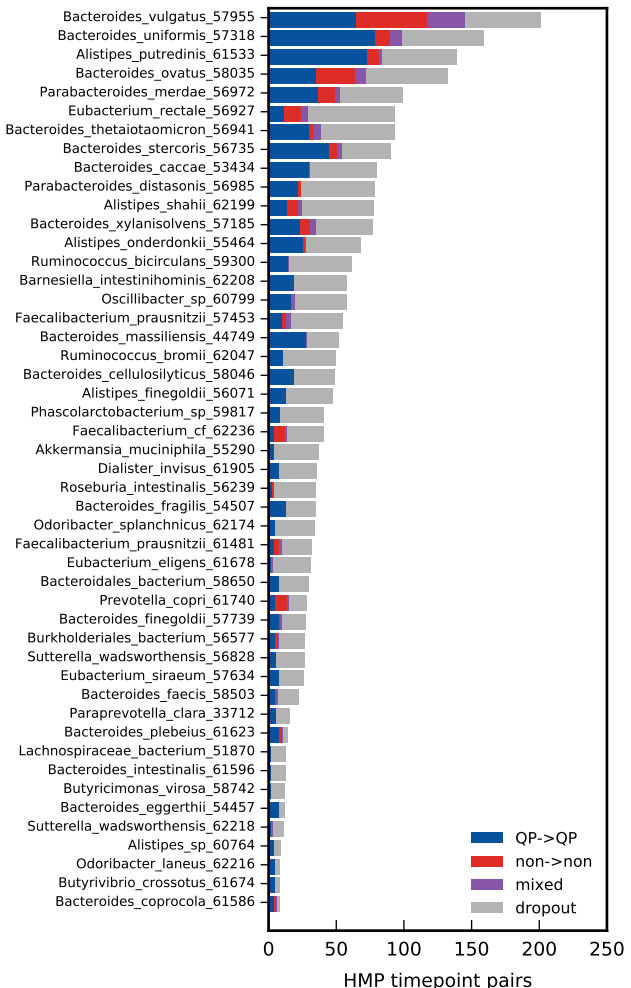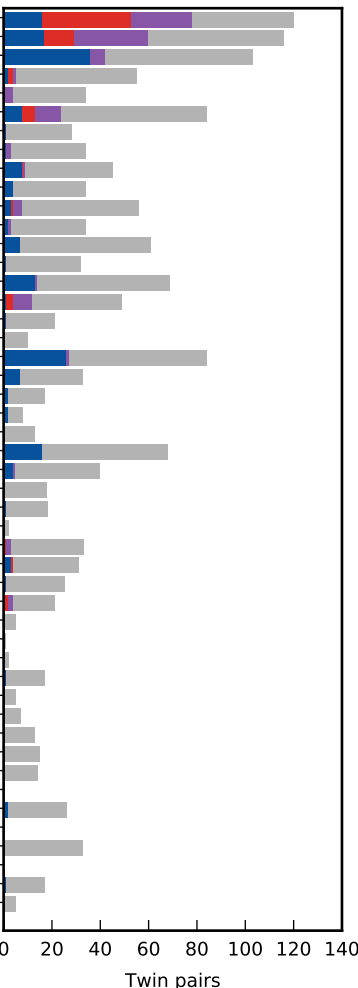

Supplement: S8 Fig — Bars show the number of sample pairs for each species that are QP for both samples (QP→QP), non-QP for both samples (non→non), mixed samples (QP→non or non→QP), and pairs in which the species did not have sufficient coverage in one of the two time points (dropout). The left panel shows data from longitudinally sampled individuals in the Human Microbiome Project cohort [42, 44], while the right panel compares contemporary samples from pairs of adult twins [45]. Species are ordered in decreasing order of prevalence in the HMP cohort. Species are only included if they have at least 10 QP samples and at least 3 QP time point pairs. (PDF) [file pbio.3000102.s008.pdf]

Sample A (D=190)

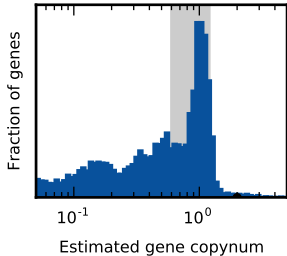

Sample B (D=124)

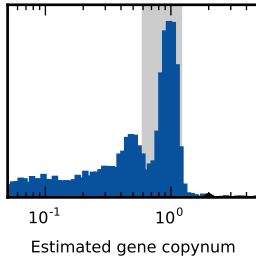

Sample C (D=293)

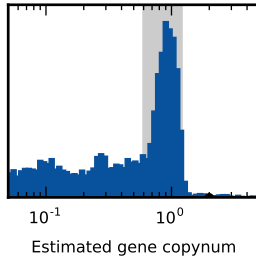

Sample D (D=175)

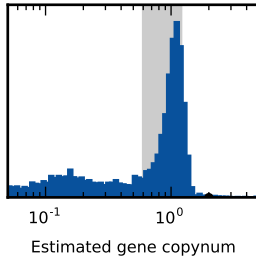

Supplement: S9 Fig — The gray region denotes the copy number range required in at least one sample to detect a difference in gene content between a pair of samples (see S1C Text, part v). (PDF) [file pbio.3000102.s009.pdf]

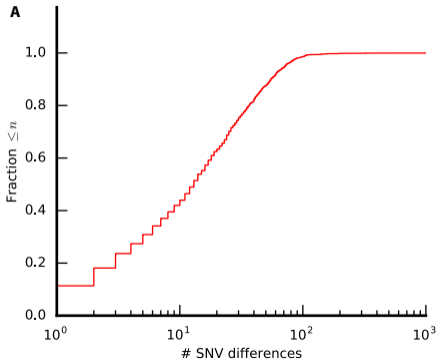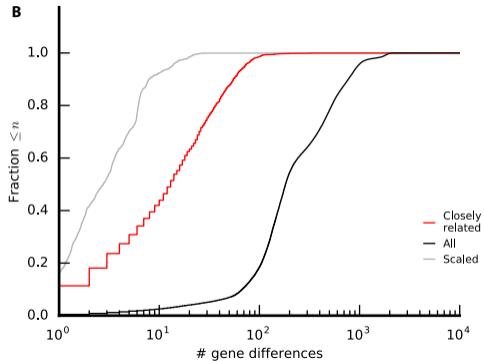

Supplement: S10 Fig — (a) Cumulative distribution of the total number of core genome SNV differences between closely related strains in Fig 2. (b) Cumulative distribution of the number of gene content differences for the closely related strains in panel a (red line). For comparison, the corresponding distribution for all pairs of strains in Fig 2 is shown in black, while the gray line denotes a “clocklike” null distribution for the closely related strains, which assumes that genes and SNVs each accumulate at constant rates. (PDF) [file pbio.3000102.s010.pdf]

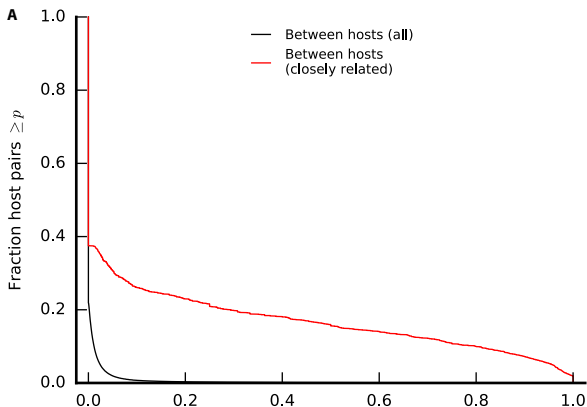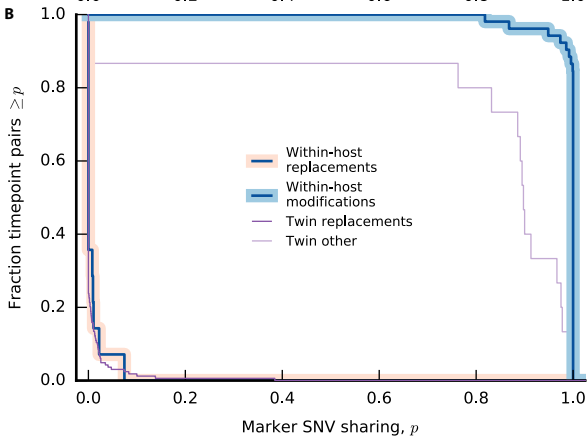

Supplement: S11 Fig — Given an ordered pair of quasi-phaseable strains, we define private marker SNVs to be core genome SNVs that (i) are phaseable in both strains, (ii) have the derived allele in strain 1, and (iii) do not have the derived allele in any other host outside the pair. The marker sharing fraction p is then defined as the fraction of private marker SNVs that also have the derived allele in strain 2. (a) Private marker SNV sharing between unrelated hosts. Solid lines show the distribution of marker sharing fraction p between all pairs of strains in Fig 2 (black) and between the subset of closely related strains (red). Separate sharing fractions are calculated for both orderings of a given strain pair, and we only include pairs with at least 10 marker SNVs. (b) Distribution of marker SNV sharing for replacement and modification events in longitudinally sampled Human Microbiome Project hosts (blue lines), using the replacement and modification thresholds in Fig 5A. For comparison, the distribution of marker SNV sharing between strains in pairs of adult twins is shown in purple. For twins, we use modified definitions of replacement (>103 SNV differences) and modification (<103 SNV differences). As above, sharing fractions are only computed for samples with at least 10 marker SNVs. (PDF) [file pbio.3000102.s011.pdf]

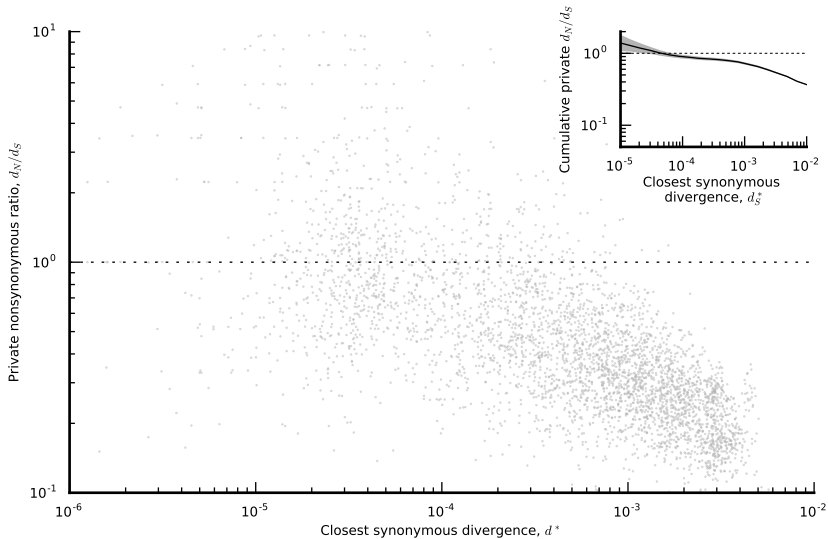

Supplement: S12 Fig — An analogous version of Fig 3 computed for private SNVs. For each quasi-phaseable (QP) species × host combination, dN/dS is computed for the subset of alleles that are not found in any other hosts. These private dN/dS ratios are plotted as a function of dS*, an estimate of the minimum synonymous divergence from other QP lineages of that species. The inset shows the ratio between the cumulative dN and dS values for all lineages with dS* less than the indicated value. The narrow shaded region denotes 95% confidence intervals estimated by Poisson resampling. The resampling procedure uses an analogous version of the thinning scheme employed in Fig 3 to ensure that the x and y axes are statistically independent (see S1D Text). (PDF) [file pbio.3000102.s012.pdf]

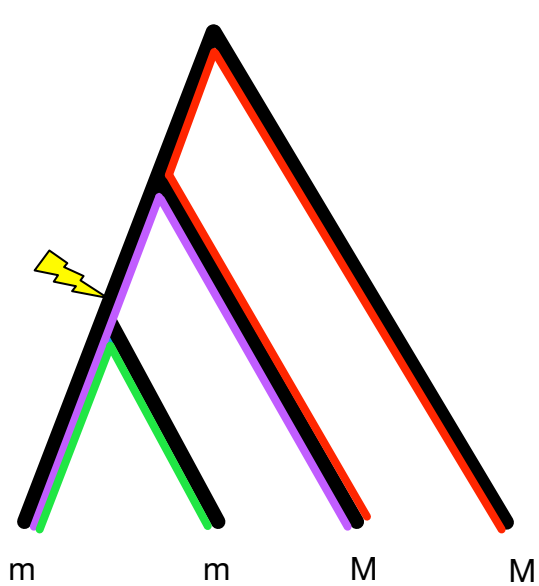

Consistent:  $d_B > d_w^m$

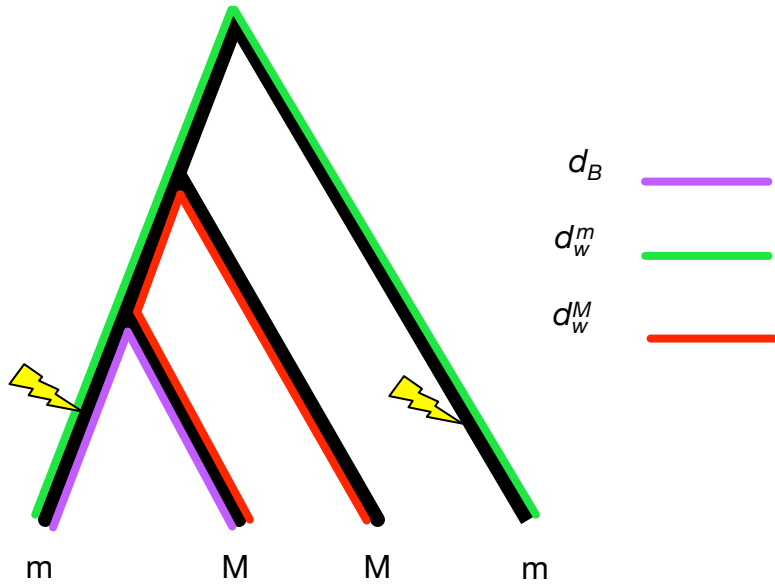

Inconsistent:  $d_B < d_w^M$

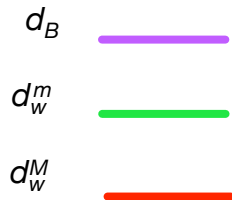

Supplement: S13 Fig — Two examples are shown, illustrating phylogenetically consistent and inconsistent SNVs, respectively, in a sample of 4 lineages. The lineages at the leaves of each tree are labeled according to whether they have the major (M) or minor (m) allele. Thunderbolts depict the most parsimonious introduction of the derived allele on the genealogy. Different colors indicate the core-genome-wide divergence between lineages with different combinations of alleles, as described in S1E Text, part i. Highlighted in purple is dB, which is the minimum divergence between two lineages bearing different alleles. Highlighted in red and green are dWM and dWm, which are the maximum divergence between individuals bearing the same allele (major and minor, respectively). In practice, we do not know which allele is ancestral and which is derived, so we define dW=min(dWm,dWM). If dW≫dB, we say that the SNV is phylogenetically inconsistent. (PDF) [file pbio.3000102.s013.pdf]

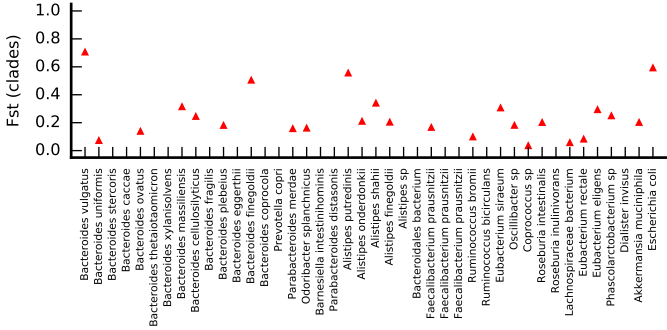

Supplement: S14 Fig — Core-genome-wide Fst between manually assigned top-level clades in each species (S2 Table, S1E Text, part ii). Species are only included if there are at least 2 clades with more than 2 individuals in each of them. (PDF) [file pbio.3000102.s014.pdf]

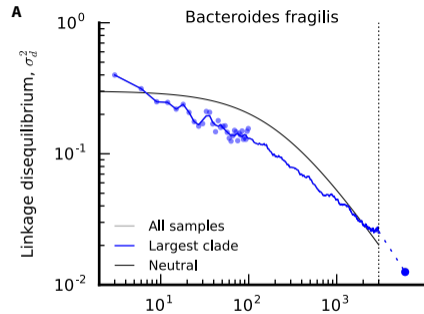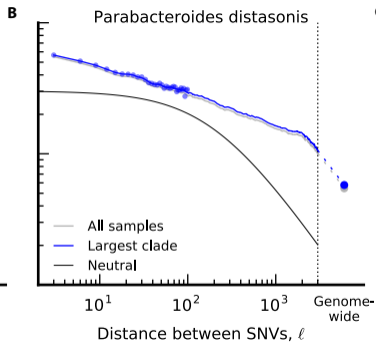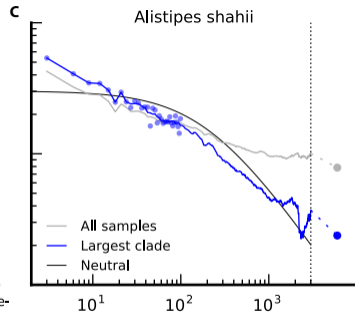

Supplement: S15 Fig — Analogous versions of the insets in Fig 4B for Bacteroides fragilis, Parabacteroides distasonis, and Alistipes shahii. (PDF) [file pbio.3000102.s015.pdf]

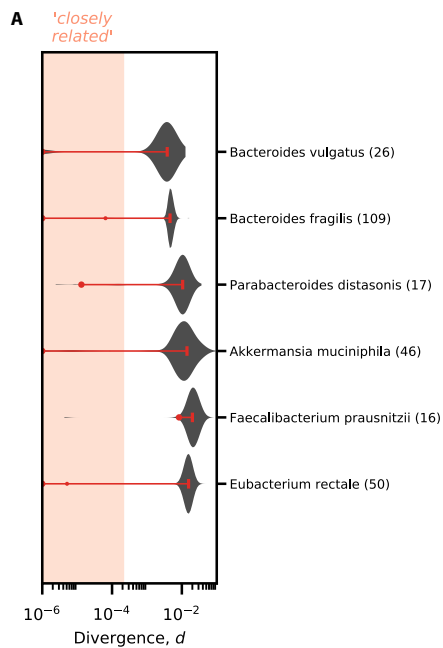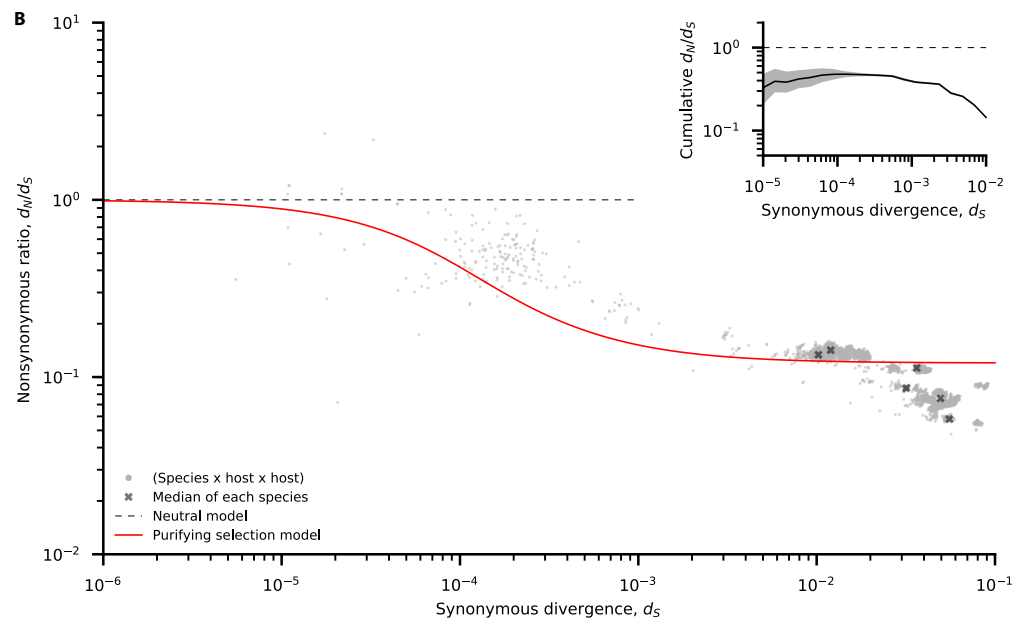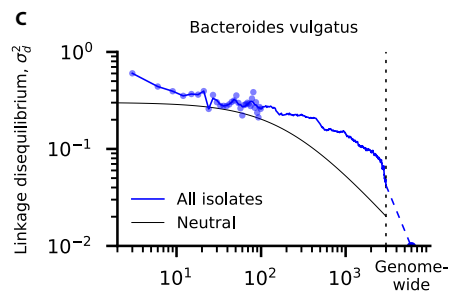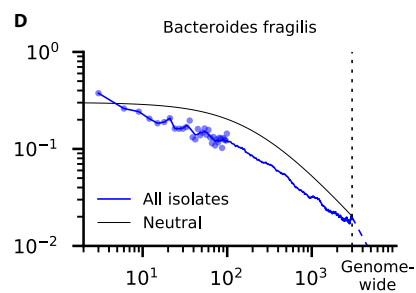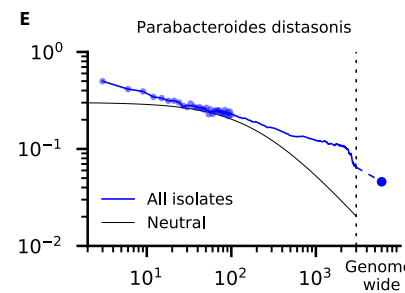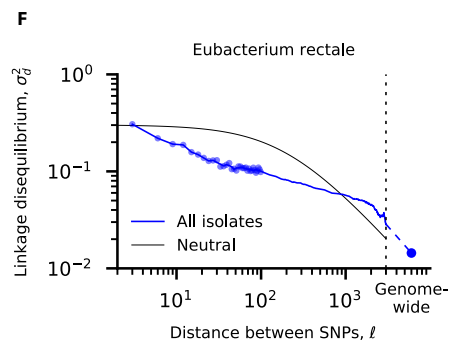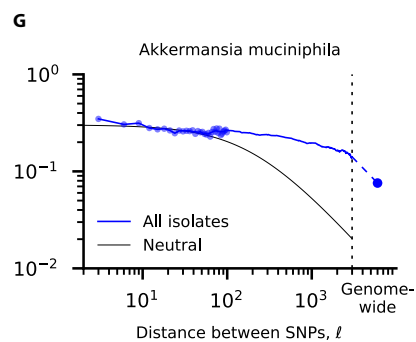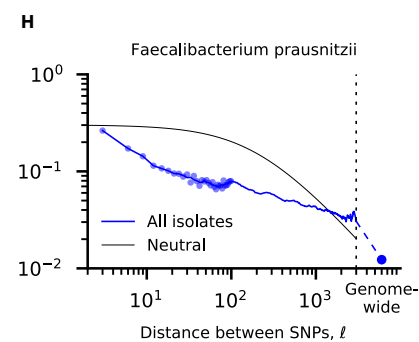

Supplement: S16 Fig — (a) An analogous version of Fig 2B constructed from the genomes of sequenced isolates in 6 representative species, as described in S1G Text. (b) An analogous version of Fig 3 constructed from the pairs of isolate genomes in panel a. (c-h) Analogous versions of Fig 4B inset for the 6 species in (a). (PDF) [file pbio.3000102.s016.pdf]

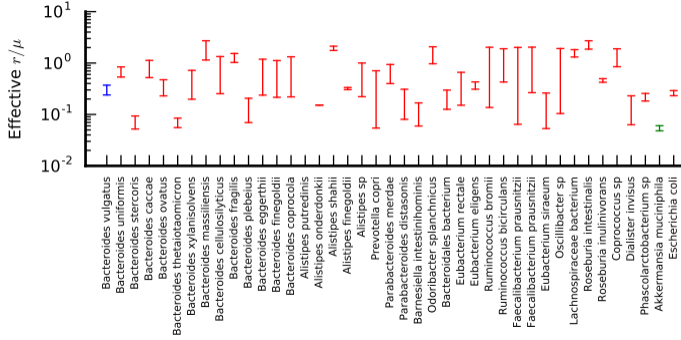

Supplement: S17 Fig — For each species, the two dashes represent effective values of r/μ estimated from the neutral prediction for the decay of σd2(l), using the half-maximum and quarter-maximum decay lengths (see S1F Text). The 2 estimates are connected by a vertical line for visualization. The overall rates of recombination are qualitatively consistent with observations in several other bacterial species [16, 73–77]. (PDF) [file pbio.3000102.s017.pdf]

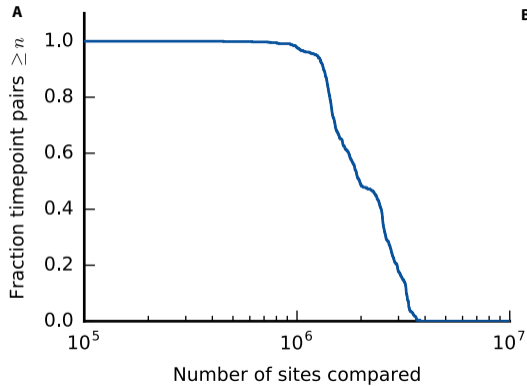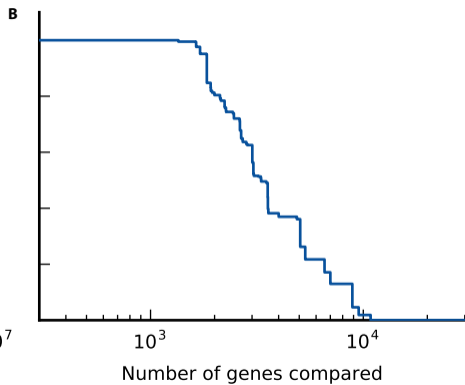

Supplement: S18 Fig — Distribution of the number of sites (a) and genes (b) tested in each of the within-host comparisons in Fig 5. (PDF) [file pbio.3000102.s018.pdf]

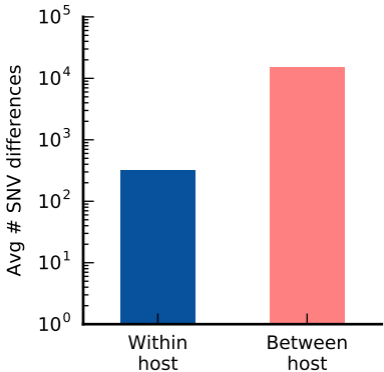

Supplement: S19 Fig — Blue and red bars denote the average of the within- and between-host distributions in Fig 5A. Consistent with previous work [31, 44], the within-host average is about 100-fold lower than the between-host average. However, the average is a poor summary of the typical values in the between-host distribution in Fig 5A. Instead, the within-host average is well approximated by the product of the typical number of SNV differences per replacement and the overall fraction of replacement events. (PDF) [file pbio.3000102.s019.pdf]

# HMP timepoint pairs

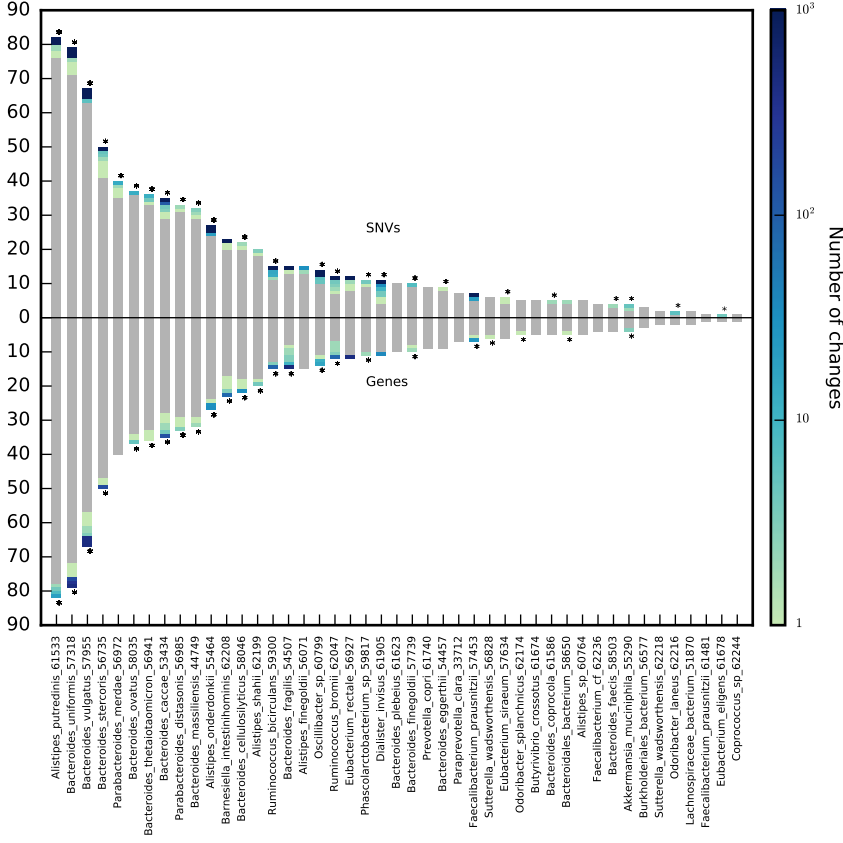

Supplement: S20 Fig — Summary of within-host SNV changes (top) and gene changes (bottom) across all species with at least 10 quasi-phaseable samples and at least 3 pairs of longitudinal QP samples. Each row in each bar represents a different longitudinal pair from the Human Microbiome Project cohort, and rows are colored according to the total number of SNV changes (top) and gene changes (bottom), with gray indicating no detected changes. A star indicates that the total number of non-replacement changes is ≥10 times the total estimated error rate across samples from that species (see S1C Text, part iv, and S1C Text, part v), in which replacements are defined as in Fig 5. (PDF) [file pbio.3000102.s020.pdf]

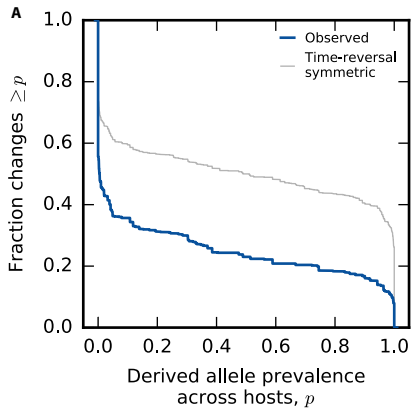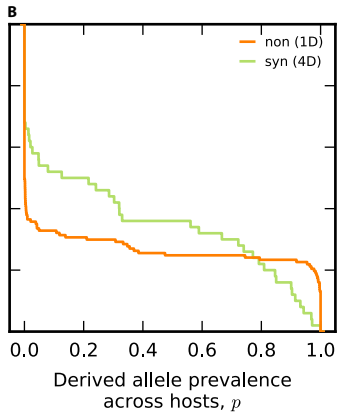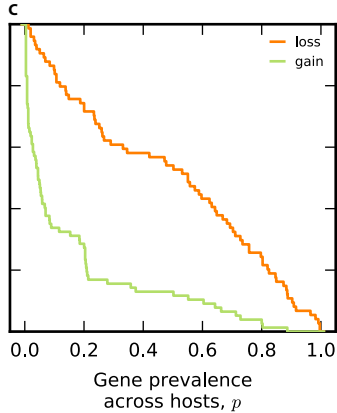

Supplement: S21 Fig — (a) The empirical survival function for the raw prevalence values in Fig 5C. For comparison, the gray line shows the time-reversal symmetric version described in S1H Text, part iii. (b) Empirical prevalence distributions for synonymous (1D) and nonsynonymous (4D) differences in Fig 5C. (c) Empirical prevalence distributions for gene gains and losses in Fig 5D. (PDF) [file pbio.3000102.s021.pdf]

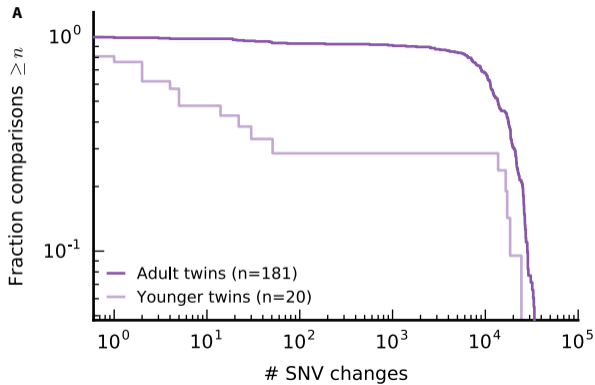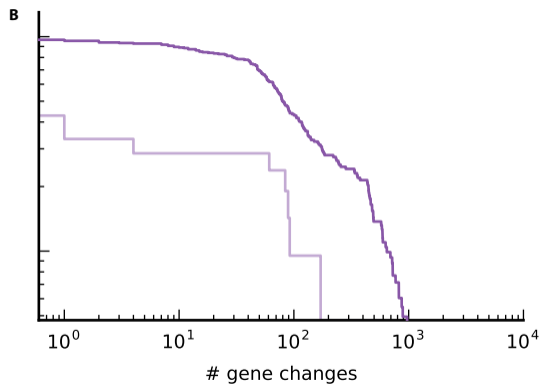

Supplement: S22 Fig — Light purple lines denote analogous versions of Fig 5A and 5B for 4 twin pairs from [46], which range from about 5 to about 20 years of age. The results are consistent with the original findings in [46]. For comparison, the dark purple lines reproduce the adult twin distributions from Fig 5A and 5B. These data show that strains from younger twins are significantly more similar to each other than strains from adult twins (P<10−4, permutation Kolmogorov–Smirnov test [91]). (PDF) [file pbio.3000102.s022.pdf]

# SNV changes

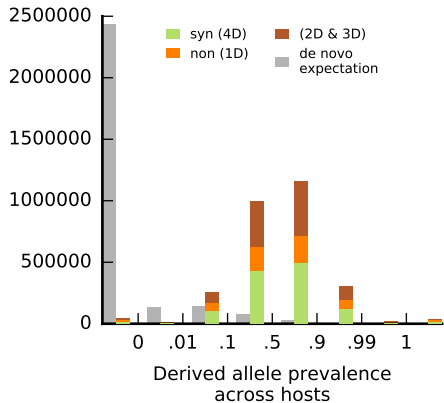

# gene changes

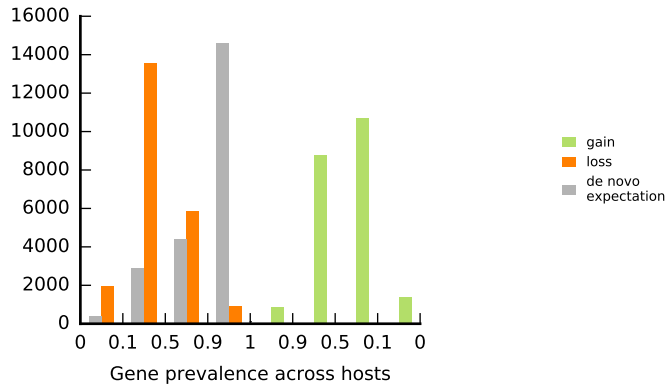

Supplement: S23 Fig — Analogous versions of Fig 5C and 5D computed using the SNV and gene content differences observed between all adult twin pairs (purple lines in Fig 5A and 5B). In contrast to the within-host changes in Fig 5C and 5D, the prevalence distributions and the relative fraction of nonsynonymous differences between twins are more consistent with replacement by a distantly related strain. (PDF) [file pbio.3000102.s023.pdf]
