# Supplementary material for: Evolutionary dynamics of bacteria in the gut microbiome within and across hosts
Source: PLoS Biol. 2019 Jan 23;17(1):e3000102. doi: 10.1371/journal.pbio.3000102 (PMC6361464; doi:10.1371/journal.pbio.3000102)
Supplement: S24 Fig — (PDF) [file pbio.3000102.s024.pdf]

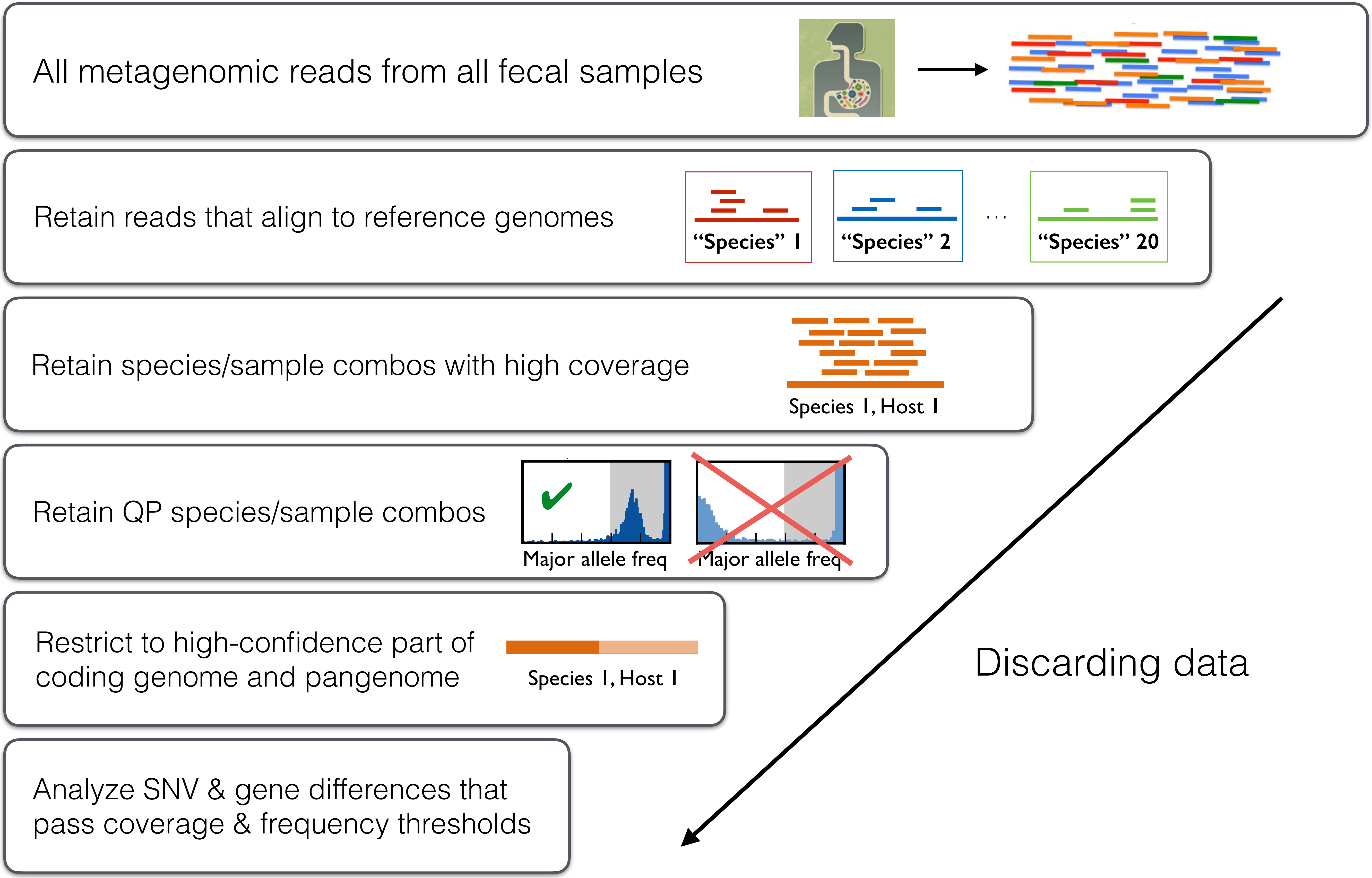
All metagenomic reads from all fecal samples
Retain reads that align to reference genomes
"Species" 1
"Species" 2
...
"Species" 20
Retain species/sample combos with high coverage
Species 1, Host 1
Retain QP species/sample combos
Major allele freq
Major allele freq
Restrict to high-confidence part of coding genome and pangenome
Species 1, Host 1
Analyze SNV & gene differences that pass coverage & frequency thresholds
Discarding data
