# Supplementary material for: Evolutionary dynamics of bacteria in the gut microbiome within and across hosts
Source: PLoS Biol. 2019 Jan 23;17(1):e3000102. doi: 10.1371/journal.pbio.3000102 (PMC6361464; doi:10.1371/journal.pbio.3000102)
Supplement: S1 Text — (A) Metagenomic pipeline. (B) Quantifying within-species diversity in individual samples. (C) Quasi-phasing metagenomic samples. (D) Population genetic null model of purifying selection for pairwise divergence across hosts. (E) Phylogenetic inconsistency and clade structure across hosts. (F) Population genetic null model for the decay of linkage disequilibrium. (G) Validation of between-host patterns using isolate sequences. (H) Quantifying prevalence of within-host single nucleotide variant and gene changes. (PDF) [file pbio.3000102.s027.pdf]

Methods and Supplemental Information for

Evolutionary dynamics of bacteria in the gut microbiome within and across hosts

Nandita R. Garud☯*, Benjamin H. Good☯*, Oskar Hallatschek, Katherine S. Pollard

☯These authors contributed equally and are ordered alphabetically
* benjamin.h.good@berkeley.edu (BHG); nandita.garud@gmail.com (NRG)

List of figures and tables

Fig 1. Genetic diversity within hosts.

Fig 2. Between-host divergence across prevalent species of gut bacteria.

Fig 3. Signatures of selective constraint within species as a function of core-genome divergence.

Fig 4. Recombination between strains across hosts.

Fig 5. Within-host changes across prevalent species of gut bacteria.

S1 Fig. Example within-host allele frequency distributions for 24 additional species (1/2).

S2 Fig. Example within-host allele frequency distributions for 24 additional species (2/2).

S3 Fig. Rates of within-host polymorphism for 24 additional species.

S4 Fig. Schematic depiction of phasing and substitution errors.

S5 Fig. Average genetic distance between *B. vulgatus* metagenomes.

S6 Fig. Correlation between within-host diversity and the fraction of non-QP samples per species.

S7 Fig. Distribution of the number of QP species per sample.

S8 Fig. Distribution of quasi-phaseable (QP) samples in longitudinal samples and adult twin pairs.

S9 Fig. Distribution of estimated gene copy numbers for the four samples in Fig 1.

S10 Fig. SNV and gene content differences between closely related strains.

S11 Fig. Private marker SNV sharing within and between hosts.

S12 Fig. Signatures of selective constraint within private SNVs.

S13 Fig. Schematic illustration of phylogenetic inconsistency between individual SNVs and core-genome-wide divergence.

S14 Fig. Top-level clade structure among lineages in different QP hosts.

S15 Fig. Decay of linkage disequilibrium in three example species.

S16 Fig. Recapitulating patterns of between-host evolution from sequenced isolates.

S17 Fig. Recombination rate estimates based on the decay of linkage disequilibrium.

S18 Fig. Distribution of the number of sites (a) and genes (b) tested in each of the within-host comparisons in Fig 5.

S19 Fig. Average number of SNV differences within and between hosts.

S20 Fig. Comparable rates of within-host SNV and gene changes across prevalent species.

S21 Fig. Prevalence distributions of within-host SNV and gene content differences without binning.

S22 Fig. SNV and gene content differences between younger twins.

S23 Fig. Prevalence of SNV and gene content differences between adult twins.

S24 Fig. Schematic figure illustrating the data discarded at various steps in our pipeline.

S1 Table. Metagenomic samples used in study.

S2 Table. Top-level clade definitions.

S1A Text

Metagenomic pipeline

Overview

We analyzed whole-genome sequence data from a panel of stool samples from 693 healthy human subjects (S1 Table). As described in the main text, this panel includes 250 North American subjects sequenced by the Human Microbiome Project [1, 2], a subset of which were sampled at 2 or 3 timepoints roughly 6-12 months apart. We also included a cohort of 125 pairs of adult twins from the TwinsUK registry [3], 4 pairs of younger twins from Ref. [4], and 185 Chinese subjects sequenced in Ref. [5].

Previous work has shown that there is little genomic variability between technical and sample replicates in HMP data [2, 6], so we merged fastq files for technical and sample replicates from the same time point to increase coverage to resolve within-host allele frequencies. We analyzed the gene and SNV content of these samples using the MIDAS software package [v1.2.2 [6]] as a foundation, with multiple additional layers of filtering implemented in custom postprocessing scripts, described below. This postprocessing pipeline was designed to be as inclusive as possible in the early steps, when hard thresholds are required, so that we could adaptively estimate thresholds from the data to use in later postprocessing steps. Later rounds of postprocessing impose a set of progressively more conservative filters, which are designed to rule out mapping artifacts and other metagenomic ambiguities, at the expense of reduced genome and species coverage. We ultimately apply this pipeline to estimate SNV and gene content changes in species with sequencing coverage of 20x or more, so our filters are designed with these numbers in mind.

All necessary metadata, as well as the source code for the sequencing pipeline, downstream analyses, and figure generation, are available at GitHub (`https://github.com/benjaminhgood/microbiome_evolution`).

A.i Estimating a panel of reference species for each host

The first step in the pipeline is to determine which species to include in the personalized reference panel for each host. The goal is to include as many truly present species as possible (to prevent their reads from being *donated* to other reference genomes) while leaving out species that are truly absent (to prevent their reference genomes from *stealing* reads from other species). To determine this set, MIDAS first quantifies the relative abundances of species in different metagenomic samples by mapping sequencing reads to a database of universal single-copy "marker" gene sequences for each of the species in the default MIDAS database (version 1.2, downloaded on November 21, 2016 [6]). We include a species in the reference panel for a given sample if it has an average marker gene coverage ≥ 3 in that sample. This definition leaves out many species that are present at lower abundances. We note, however, that their coverage would be too low for them to be included in our downstream analyses, and any donated reads would add only fractional contributions to the polymorphism frequencies for species in our target coverage range.

For longitudinally sampled individuals, we defined a single reference panel for each host by including all species with marker coverage ≥ 3 in at least one timepoint. This choice is designed to reduce potential mapping artifacts by ensuring that all longitudinal comparisons are performed with the same mapping parameters.

A.ii Quantifying gene content

We next quantified the gene content for each species present in each sample. In downstream analyses, gene content information was used to estimate the prevalence of genes in the broader population, and to quantify gene content differences between QP samples (C.v).

MIDAS estimates gene copy number for each species by mapping reads to a database of gene families (or *pangenome*) constructed from genes in sequenced isolates [6]. This approach has been adopted in a number of related methods [7, 8], along with similar methods based on co-occurence or binning [5, 9, 10]. Briefly, pre-computed pangenomes are supplied for each species in the default MIDAS database, and a host-specific database is constructed by concatenating pangenomes from each species in the personalized reference panel. Sequencing reads are aligned to this host-specific database using Bowtie2 [11] with default MIDAS settings (local alignment, MAPID ≥94.0%, READQ ≥20, and ALN_COV ≥0.75), and the average coverage is estimated by dividing the total number of mapped reads in a gene family by the total target size. We note that with these settings, reads with multiple best-hit alignments will be distributed among these targets according to their proportional representation on the pangenome reference sequence; these reads were retained to ensure consistent estimates of average coverage within a gene family, which might contain multiple highly similar genes [6].

For each species, average coverage was reported for each gene, as well as for a panel of universal, single-copy marker genes [6, 12]. The copynumber of a gene (c) is then estimated as the ratio between its coverage and the (median) marker gene coverage. We used these raw copynumber values to estimate the prevalence of genes in the broader population, defined as the fraction of samples with $c \geq 0.3$ (conditioned on the marker coverage being $\geq$ 5x). Ref. [6] have previously shown that these thresholds yield accurate gene presence estimates. We then used these prevalence estimates to define a *core genome* for each species, defined as the set of genes with prevalence ≥ 0.9.

In addition to quantifying gene prevalence, we also used MIDAS's copynumber estimates to detect changes in gene content between QP samples (C.v). The QP methodology was designed to eliminate spurious gene content differences that arise from sampling noise, e.g. when a host is colonized by multiple strains of the same species. However, another well-known limitation of the pangenome approach used by MIDAS and others is that linkage between a gene and its species is not observed directly, but is only inferred by the presence of that gene in a previously sequenced isolate. This can lead to spurious copynumber changes if a target gene is actually linked to a different species in a particular host, and the relative abundance of the species are simply changing over time. To guard against this scenario, we implemented a number of additional filters described below.

First, we only considered gene content differences that were consistent with a single copy gene transitioning to zero copynumber, or vice versa. We used a permissive definition of potential single copy genes ($0.6 \leq c \leq 1.2$, with marker coverage $\geq 20x$) in order to capture normal coverage variation along the genome in growing cells [13], see S9 Fig. Similarly, we defined a zero copynumber to be $c \leq 0.05$, so that a small fraction of cells could still retain the gene. (For simplicity, these copynumber thresholds are also used for the sampling error calculation in C.v.) We implemented this copynumber restriction because, if a gene is truly linked to a different species, it is less likely to have both a "normal" and "absent" copynumber by chance. For this to happen, it would require that the two species that share the gene in a given host (a rare event) have similar relative abundance at one timepoint and ≥ 10-fold different abundance at the other (another rare event). Although this approach omits many biologically interesting copynumber differences among multi-copy genes (e.g. transporter genes in *Bacteroides* [14]), we do not study them here because they are much harder to disentangle from mapping artifacts.

To supplement these copynumber filters, we also created a blacklist of genes that are potentially shared across species. This is helpful for some highly promiscuous genes, e.g. transposons in *Bacteroides* [15], where the probability of cross-species sharing cannot be assumed to be low. We constructed this blacklist by searching for gene families in the MIDAS database that had sequence similarity $\geq 95\%$ with a gene family in another species (A.iv). These families constitute a gold standard for gene sharing events, since they imply that highly similar genes have been observed in isolates from different species. However, this approach can also miss cases of gene sharing for species with poor phylogenetic coverage in the MIDAS isolate database.

We therefore supplemented the isolate-based blacklist with gene sharing candidates that were identified directly from the metagenomic data. In particular, we defined a putatively shared gene to be one with $c \geq 3$ in at least one sample in our cohort, since this could indicate read donating by a shared gene in a more abundant species. This does not constitute proof of gene sharing, but it is conservative for the purposes of constructing a blacklist. The metagenomic and isolate-based methods identified many common gene sharing candidates, but for many species, there were also many genes that were only identified by one of the two approaches.

All genes in the combined blacklist were excluded from downstream analyses of gene content estimation and SNV calling. As with our copynumber filters above, this likely omits many biologically interesting regions of the genome, since shared genes are arguably more likely to play a role in short-term evolutionary dynamics. We ignore them here in order to minimize false positives created by read donating.

Even with these various filters, it is important to note that the pangenome approach employed by us and others is at best an inferential method, which relies on an out-of-sample estimate of linkage to the correct species background. While we have included these gene content differences to supplement our SNV-based analysis, isolate sequences [15] or long read data [16] are required to definitely prove that any specific gene content difference is linked to the species of interest.

A.iii Quantifying SNVs

We next quantified single nucleotide variants (SNVs) for each species in each sample. In downstream analyses, these calls were used to quantify SNV prevalence across our cohort, to identify QP samples, and to quantify SNV differences between QP samples.

Similar to our pipeline for identifying gene content, MIDAS uses a standard reference based approach to identify SNVs in metagenomic data. Briefly, sequencing reads were first aligned to the host-specific panel of reference genomes using Bowtie2, with default MIDAS mapping thresholds: global alignment, MAPID ≥94.0%, READQ ≥20, ALN_COV ≥0.75, and MAPQ ≥20. Species were immediately excluded from further analysis if $\leq 40\%$ of the genome (the typical core genome fraction) recruited any reads; these excluded cases likely correspond to scenarios where the species is not truly present, but reads from some accessory genes are instead recruited from a different species. Gene annotations for each reference genome were lifted over from the PATRIC database [17], and protein coding sites were classified as 1-fold, 2-fold, 3-fold, or 4-fold degenerate based on the codon reading frame of each annotated gene.

Based on these raw alignments, MIDAS reports the total read coverage D for each site in the reference genome for each given sample [18]. We used this distribution of coverage across the genome to obtain a measure of the "typical" coverage, $\overline{D}$, defined as the median of all protein coding sites with nonzero coverage. All samples with $\overline{D} < 5$ were excluded from further analyses. Additional coverage requirements for QP samples are imposed below.

We then used the sample-specific estimates of $\overline{D}$ to refine the alignment step above, since $\overline{D}$ helps to calibrate our expectation for the coverage at a given site in the genome (D). In particular, sites with $D \ll \overline{D}$ could arise due to mapping errors or read donating from less abundant species, if the reference genome contains regions that are not present in a given sample. Similarly, sites with $D \gg \overline{D}$ could arise from multi-copy genes, or read donating from more abundant species. To exclude these cases, we masked sites in a given sample if $D < 0.3\overline{D}$ or $D > 3\overline{D}$. This constitutes a slightly more permissive version of our single-copy criterion above, due to the larger uncertainties inherent in estimating D. As above, we only considered sites in coding sequences of annotated genes, and sites that were unmasked in fewer than 4 samples were excluded from all further analyses.

For each of the retained sites, MIDAS reports reference and alternate allele counts using samtools mpileup [18]. (In a minority of cases where multiple alternative alleles were present, these are merged into a single class.) We used these raw allele counts to estimate the prevalence of SNVs in the broader population, defined as the fraction of samples where the alternate allele

comprises the majority of reads. Since the reference allele is arbitrarily defined by the choice of reference genome, we used these prevalence estimates to polarize each SNV based on the consensus across the cohort. Polarized within-sample allele frequencies were then defined as the fraction of reads supporting the allele with the lower prevalence across the cohort.

These allele frequencies are used to identify QP samples (C.iii) and to ultimately quantify SNV differences between QP samples (C.iv). As with the gene content estimates above, these SNV differences are also susceptible to false positives that occur if the two alleles are actually linked to different species that are simply fluctuating in abundance. We have implemented a number of filters to guard against these events.

First, the global alignment and the MAPQ settings in Bowtie2 already ensure that reads must have a particularly unique match to their assigned reference genome. We only considered sites in protein coding genes, and we excluded all putatively shared genes in the blacklist above. In contrast to previous polymorphism- [19] or consensus-based approaches [2, 20], we considered only extreme changes in allele frequency (≤ 0.2 to ≥ 0.8, or vice versa) to ensure that the SNV difference is supported by the vast majority of the reads at both timepoints, rather than a fraction of reads donated from other species. Combined with the coverage requirement in both samples ($0.3\overline{D} \leq D \leq 3\overline{D}$ and $\overline{D} \geq 20$), this eliminates most opportunities for SNV differences to arise from abundance fluctuations: large fluctuations will typically violate the coverage requirement, while small fluctuations will not produce a sufficient change in allele frequency.

In cases where we compare longitudinal samples from the same host, we imposed an even stronger version of this filter to be more conservative with respect to calling SNV changes. Under the reasonable assumption that genome synteny is preserved among very closely related strains, we expect the relative coverage of a site ($D/\overline{D}$) to be more similar in longitudinal samples than the maximum 10-fold range allowed by the coverage condition ($0.3 \leq D/\overline{D} \leq 3$). Thus, in addition to the requirements above, we only called a SNV difference between two samples if the successive values of $D/\overline{D}$ were within a factor of 3.

A.iv Identifying orthologous genes in different pangenomes

The species-specific pangenomes in the MIDAS database were constructed by clustering all genes found in the isolate genomes of each species using a 95% identity threshold [6]. However, this clustering approach leaves open the possibility that a gene in one species' pangenome may have sequence similarity $\geq 95\%$ to a gene in another species' pangenome. We identified these cross-pangenome orthologs as follows.

First, for computational efficiency, we focused on human-relevant bacterial species in the MIDAS database by identifying those isolates with the keywords 'human' or 'Homo sapiens' in the host column of the PATRIC database. We also included species that had a universal single copy gene marker coverage $\geq$ 1x in at least one sample in our cohort. This resulted in 1002 human-relevant species.

Next, we ran USEARCH [21] on the set of genes belonging to the pangenomes of these human-relevant bacterial species. Based on this approach, we identified a total of 890,058 genes across these 1002 species that had $\geq 95\%$ sequence identity with at least one other gene in a different species' pangenome. These genes were excluded from further analysis as described above.

S1B Text

Quantifying within-species diversity in individual samples

B.i Estimating rates of core-genome polymorphism

To estimate the overall levels of nucleotide polymorphism for a given species in a given sample (Fig 1E), we calculate the fraction of synonymous sites in core genes with intermediate allele frequencies ($0.2 \leq f \leq 0.8$). In other words, the polymorphism rate r is defined by

$$r = \mathbb{E}[\theta(f - 0.2) \cdot \theta(0.8 - f)] \,, \tag{S1}$$

where $\theta(z)$ is the Heaviside step function. This measure is similar to the traditional population genetic measure of heterozygosity, $H = \mathbb{E}[2f(1 - f)]$, which places the most weight near intermediate allele frequencies. The thresholded version in Eq (S1) is preferable in our case, as it is more robust to low-frequency sequencing errors that can overwhelm the average in H.

To obtain the approximate confidence intervals for the rates in Fig. 1E, we used a standard Bayesian procedure based on a poisson approximation. If we let L denote the total number of sites examined and let n denote the number of "successes" (i.e., the number of intermediate frequency polymorphisms), then we assume that n is drawn from a Poisson

$$n \sim \mathrm{Poisson}(rL) \,, \tag{S2}$$

where r is the per site rate plotted in Figs. 1E. Since r is a positive quantity that varies over many orders of magnitude, we use a uniform prior over $\log r$. After applying Bayes' rule, this yields a standard conjugate Gamma posterior distribution for r:

$$p(r|n, L) = \frac{L^n}{(n-1)!} r^{n-1} e^{-rL} \,. \tag{S3}$$

whose posterior mean is just

$$\int r p(r|n, L) \, dr = \frac{n}{L} \,, \tag{S4}$$

as expected. For all $n > 0$, we define a $1 - \alpha$ confidence interval to be the $\alpha/2$ and $1 - \alpha/2$ percentiles of this posterior distribution. In the case where $n = 0$, the posterior distribution is improper:

$$p(r|0, L) \propto r^{-1} e^{-rL} \,. \tag{S5}$$

In this case, we define the lower limit of the confidence interval to be 0, and the upper limit to be the point where $e^{-rL} \sim \alpha/2$.

B.ii Within-host evolution in a single-colonization model

In this section, we further explain the assumptions made in computing the expected within-host polymorphism rate for a given species under a simple, single-colonization model. As described in the text, we make conservatively high estimates for the per site mutation rate ($\mu \sim 10^{-9}$ per generation), generation times ($\lambda \sim 10$ generations per day), and time since colonization ($\Delta t \sim 100$ years). We define the within-host polymorphism rate P as the fraction of fourfold-degenerate synonymous site mutations with allele frequencies in the range $0.2 \leq f \leq 0.8$. In the single-colonization model, the mutations that contribute to P must have reached intermediate frequencies after starting as a *de novo* mutation at some time after colonization.

We assume that the synonymous mutations are effectively neutral over the timespans considered ($s\lambda\Delta t \ll 1$). Under this assumption, one of these mutations can only contribute

to P if it hitchhiked along with a lineage that rose to a frequency in the range $0.2 \leq f \leq 0.8$. This can happen either due to neutral drift (i.e., the lineage randomly fluctuated to intermediate frequencies) or selection (i.e., the lineage reached intermediate frequencies because it contains a beneficial mutation). However, if synonymous mutations are neutral, their presence or absence in a lineage is independent of the processes that drive it to intermediate frequency [22]. The probability that a particular neutral mutation arose along the line of descent is simply the product of the per-site mutation rate μ and the total number of generations since the lineage diverged from the common ancestor between it and the rest of the population. By assumption, the latter is bounded by the total number of generations since colonization ($\lambda\Delta t$). This yields the conservative estimate for the within-host polymorphism rate,

$$P \leq \mu\lambda\Delta t \leq 10^{-3}\,, \tag{S6}$$

quoted in the main text.

S1C Text

Quasi-phasing metagenomic samples

In this section, we describe the methods used to estimate one of the dominant haplotypes for a given species in a subset of metagenomic samples (the so-called *quasi-phaseable* or QP samples), and to quantify genetic differences between these lineages. The method is similar in spirit to recent work by Ref. [20], but with a greater emphasis on estimating the associated false positive rates.

C.i Theoretical motivation

To gain intuition for how within-host lineage structure is reflected in the distribution of allele frequencies, it is useful to start by considering the simplest version of the phasing problem, in which the metagenomic reads for a given species in a particular sample are derived one of two clonal lineages mixed in a proportion $f_{\rm mix} \geq 50\%$ (representing the proportion of cells from the more abundant lineage). Within-sample polymorphisms will arise from fixed differences between the two lineages and will segregate at frequency $f_{\rm mix}$ or $1 - f_{\rm mix}$, depending on which lineage the mutation arose in and the choice of reference allele. Since this choice is arbitrary, we work with the major allele frequency in each sample. In this case, the distribution of major allele frequencies, $p(f)$, will then have the simple form

$$p(f) = (1-d) \cdot \delta(1-f) + d \cdot \delta(f - f_{\rm mix}), \tag{S1}$$

where d is the average nucleotide divergence between the two lineages and $\delta(z)$ is the Dirac delta function. Note that this theoretical distribution is only obtained in the limit of infinite coverage; in practice, the observed distribution of major allele frequencies will be blurred due to sampling noise (see Section C.ii below). Nevertheless, in the the limit of high coverage, Eq (S1) suggests that we can infer $f_{\rm mix}$ and d by looking for a peak in the distribution of major allele frequencies (e.g., Fig 1E). Again, in the idealized case, the two haplotype sequences are easy to recognize: major alleles are assigned to the dominant lineage, while the minor alleles belong to the subdominant type. This is conceptually similar to the "binning" techniques that are used to assemble genomes from metagenomic contigs [23].

This basic idea also extends to mixtures of more than two lineages, but the potential genealogical relationships between them make the problem much more complicated. In these cases, the traditional "binning" heuristic may no longer apply. For example, in a mixture of three strains with frequencies f_1, f_2, and f_3, the distribution of major allele frequencies will now have three characteristic peaks (corresponding to $\min\{f_i,\ 1 - f_i\}$ for each $i = 1, 2, 3$). This time, however, alleles that segregate at the same frequency do not necessarily belong to the same lineage, since they could also be ancestral to two of the three strains. There are three possible genealogies relating the three strains, which can vary from site-to-site in the presence of recombination. Haplotype estimation then becomes a complicated inference problem, which only grows more difficult as additional lineages are added. Consideration of the combined allele frequency distribution may be helpful for deriving error models for algorithms that attempt to deconvolute strains from metagenomes.

Rather than trying to infer the exact mixture proportions and the haplotypes of each lineage, we developed a set of heuristic rules to identify the haplotype of just *one* of the dominant lineages while controlling the probability of misassigning variants to this haplotype. Suppose that there are within-sample polymorphisms at two sites, with major allele frequencies f_1 and f_2. We denote the four (unobserved) two-locus haplotype frequencies by f_{MM}, f_{Mm}, f_{mM}, and f_{mm}, where M and m denote the major and minor allele at each site. If $f_1 = f_2 = 0.5$, then there are no constraints on the possible haplotype frequencies, other than the marginal constraints $f_{MM} + f_{Mm} = f_1$ and $f_{MM} + f_{mM} = f_2$. However, in the opposite extreme where $f_1 = f_2 = 1$,

then normalization constraints require that $f_{MM} = 1$ (i.e., the major alleles are on the same haplotype). In between these two extremes there is a more general rule that, whenever the allele frequencies satisfy $f_i \geq f$, with $\log(f/1-f) = c \gtrsim 1$, the minimum possible frequency of the MM haplotype is

$$f_{MM} \geq 2f - 1 \sim 1 - 2e^{-c} \,. \tag{S2}$$

Equation S2 represents a worst case scenario in which the haplotypes are specifically assigned to prevent major alleles from segregating together. In practice, a more realistic lower bound for the f_{MM} is attained when the alleles are in linkage equibrium:

$$f_{MM} = f^2 \sim 1 - 2e^{-c} \,, \tag{S3}$$

which happens to have the same asymptotic behavior in this two-locus example. In either case, these bounds show that an appreciable fraction of cells in the host must possess both major alleles.

This argument can also be extended to larger collections of sites. In the pessimistic case of linkage equilibrium between all polymorphic sites, the number of major alleles per individual is binomially distributed with success probability f. In the limit of a large number of sites, this means that the vast majority of the cells will have the major allele at a fraction f of the possible sites. However, while the haplotype consisting of all major alleles is the most likely haplotype under linkage equilibrium, its expected frequency can grow quite small, to the point where the haplotype may not even be present in a finite sample. Fortunately, our analysis will primarily focus on one- and two-locus statistics where the stronger bounds in Eq (S2) can be applied.

C.ii False positive rate for SNV phasing

The arguments above suggest that, for many downstream purposes, we can effectively estimate a portion of one of the haplotypes in a metegenomic sample by taking the major alleles present above some threshold freuqency, $f^* \gg 50\%$, and treating sites with intermediate frequencies as missing data. This is a simple generalization of the consensus method (i.e. taking the haplotype formed by all major alleles) that has been used in previous metagenomic studies [20, 24], and it is similar to methods used to genotype clonal isolates from whole-genome resequencing data [25].

The major difficulty with this approach is that we do not observe the true frequency f directly, but rather a sample frequency $\hat{f}$ that is estimated from a finite number of sequencing reads. Polarization errors (i.e. errors in determining the major allele) can therefore accumulate when the allele supported by the most reads differs from true major allele in the sample. When sequencing clonal isolates, such false positives are primarily caused by sequencing errors. These occur at a low rate per read ($p_{\text{err}} \sim 1\%$ per bp), and become increasingly unlikely at moderate sequencing depths. However, in a metagenomic sample, polarization errors will also arise due to finite sampling noise, when an allele at some intermediate frequency (e.g. 25%) happens to be sampled in a majority of the sequencing reads. As we will show below, for moderate sequencing depths, this will often be the dominant source of error.

To model this process, let (A_ℓ, D_ℓ) denote the number of alternate alleles and total sequencing depth at a given site ℓ in the genome, and let $\hat{f}_\ell = A_\ell/D_\ell$ denote the corresponding sample frequency. We assume that the number of alternate reads follows a binomial distribution,

$$\Pr[A_\ell | D_\ell, f_\ell] = \binom{D_\ell}{A_\ell} f^{A_\ell} (1-f)^{D_\ell - A_\ell} \,, \tag{S4}$$

for some true frequency f_ℓ, so that the probability of observing $\hat{f}_\ell \geq f^*$ is simply

$$\Pr[\hat{f}_\ell \geq f^* | D_\ell, f_\ell] = \sum_{k \geq f * D_\ell} \binom{D_\ell}{k} f^k (1-f)^{D_\ell - k} \,. \tag{S5}$$

A polarization error will occur when we observe $\hat{f}_\ell \geq f^*$ even though $f_\ell < 50\%$. Equation (S5) shows the probability of such an error will strongly depend on f_ℓ. For a sequencing depth of $D = 10$ and a frequency threshold of $f^* = 80\%$, the error probability ranges from essentially negligible ($\sim 10^{-14}$) when f is on the order of the sequencing error rate ($\sim 1\%$), to ~ 1 per bacterial genome when $f \approx 10\%$, to an error rate of 5% when $f \approx 50\%$.

The average false positive rate across the genome will therefore depend on an average over the possible values of f and D:

$$\Pr[\text{error}] = \int \Pr[\hat{f} \geq f^* | D, f] p_0(D, f) \, dD \, df \,, \tag{S6}$$

where $p_0(D, f)$ is the prior distribution of D and f at a randomly chosen site (S4 Fig, panel A). In the absence of any additional information, this joint distribution with the product of empirical distributions,

$$p_0(D, f) \approx \hat{p}(D)\hat{p}(f) \,, \tag{S7}$$

which we estimate for a given sample by binning the observed values of D and the allele frequencies across the L sites under consideration (blue distribution in S4 Fig, panel A). The expected number of polarization errors in a given sample across all L sites is given by

$$N_{\text{err}} = \Pr[\text{error}] \times L \,. \tag{S8}$$

This calculation holds for any large collection of sites where the empirical distribution, $\hat{p}(f)$, provides a reasonable approximation to the prior distribution, $p_0(f)$. For example, in the following section, we consider the set of all synonymous sites in the core genome.

C.iii Quasi-phaseable (QP) samples

The basic idea behind our approach is that we wish to restrict our attention to samples where N_{err} is small compared to the total number of sites under consideration. This number will vary depending on the particular analysis that we wish to carry out. But for population-genetic purposes, it will always be related to the number of sites that actually vary between samples. As a simple proxy for this number, we therefore consider a measure of the average genetic distance between the dominant haplotype in a given sample and the lineages in the remainder of our panel.

Specifically, we focus on fourfold-degenerate synonymous sites in the core genome. For each sample, let $N_<$ denote the number of such sites with major allele frequencies less than f^*, and conversely, let $N_>$ denote the number of sites with $\hat{f} \geq f^*$. For the sites in the latter group, let $\overline{f}_\ell$ denote the corresponding allele frequency across the entire panel. Then the quantity

$$N_d = \sum_{\ell=1}^{L} (1 - \overline{f}_\ell) \tag{S9}$$

approximates the expected number of differences at these sites for an "average" individual drawn from the panel. A normalized version (N_d/L) is illustrated for the *B. vulgatus* samples in S5 Fig. We declare the sample to be a **quasi-phaseable (QP)** sample if it passes the coverage thresholds in S1A Text and $N_</N_d < 0.1$.

To see why this is a reasonable definition, we return to our error formula in Eq (S8) and plug in conservative estimates for $p_0(D, f)$. For example, we expect that the number of truly polymorphic sites in the sample will also be of order $\sim N_d$, with the remaining sites having frequencies near the sequencing error threshold, $f \sim 1\%$. We then divide the remaining polymorphic sites into the fraction $N_</N_d \lesssim 0.1$ with major allele frequencies below f^*, and the remaining fraction ($\sim 100\%$) with major allele frequencies above f^*. If we make the conservative approximation

that all of the sites in the latter group have minor allele frequencies $f \approx 1 - f^*$, and all of the sites in the former group have $f \approx 50\%$, then we obtain an approximate prior distribution for f:

$$\hat{p}_0(f) \approx \frac{N_> - N_d}{N_> + N_<}\delta(f - 0.01) + \frac{N_d}{N_> + N_<}\delta(f - 1 + f^*) + \frac{N_<}{N_> + N_<}\delta(f - 0.5)\,. \tag{S10}$$

If we make a similarly conservative approximation for the coverage distribution,

$$\hat{p}(D) \approx \delta(D - 10)\,, \tag{S11}$$

where δ is the Dirac function, then for a threshold of $f^* = 80\%$, the realized false positive rate is

$$\begin{aligned} \frac{N_{\rm err}}{N_d} &\approx \frac{N_> - N_d}{N_d}\Pr[\hat{f} \geq f^*|10, 0.01] + \Pr[\hat{f} \geq f^*|10, 1 - f^*] + \frac{N_<}{N_d}\Pr[\hat{f} \geq f^*|10, 0.5] \\ &\lesssim 0.01\,. \end{aligned} \tag{S12}$$

Thus, with these thresholds, we expect that only a small fraction of informative sites (as defined by the average distance between samples) will be susceptible to polarization errors.

C.iv False positive rate for SNV differences

Although the QP sample classification is a good rule of thumb for determining when polarization errors are more or less likely to happen, there are scenarios where we wish to measure genetic distances between samples (e.g. longitudinal samples from the same individual) that are much more closely related than an average pair of individuals in our panel. In these cases, the realized false positive rate can be much higher than the estimate in Eq (S12). To obtain more accurate estimates of the error in these cases, we extend our calculation above to the specific problem of detecting the number of nucleotide differences between two samples.

Generalizing from the phasing problem above, we would conclude that the haplotypes in two samples share the same allele at a given site if that allele is present above frequency f^* in both samples. To observe a difference between the two samples, the allele would have to be present above frequency f^* in one sample and below $1 - f^*$ in another. If the allele lies between $1 - f^*$ and f^* in one of the samples, the site is treated as censored data. Under this definition, a nucleotide difference requires a change in allele frequency of at least

$$\Delta f = f^* - (1 - f^*) = 2f^* - 1\,. \tag{S13}$$

If we rewrite everything in terms of Δf, a nucleotide difference requires the allele frequency to lie below $(1 - \Delta f)/2$ in one sample and above $(1 + \Delta f)/2$ in another (pink shaded regions in S4 Fig, panel B). We will adopt the latter notation here, as it allows us to easily consider more stringent thresholds for which $\Delta f > 2f^* - 1$.

Under the null hypothesis, we assume that the true allele frequency f is the same in the two samples. If we let D_1 and D_2 denote the coverage of the site in the two samples, then a simple generalization of Eq (S6) shows that the false positive rate for a randomly chosen site is given by

$$\begin{aligned} \Pr[\text{error}] = \int &\Big\{\Pr[\,\hat{f}_1 \geq (1 + \Delta f)/2\,|D_1, f]\,\Big(1 - \Pr[\hat{f}_2 \geq (1 - \Delta f)/2\,|D_2, f]\Big) \\ &+ \Big(1 - \Pr[\hat{f}_1 \geq (1 - \Delta f)/2\,|D_1, f]\Big)\Pr[\,\hat{f}_2 \geq (1 + \Delta f)/2\,|D_2, f]\Big\} \\ &\times p_0(D_1, D_2, f)\, dD_1 dD_2 df\,, \end{aligned} \tag{S14}$$

where $\Pr[\hat{f} \geq f]$ is defined in Eq (S5) and $p_0(D_1, D_2, f)$ is the prior distribution for D_1, D_2, and f at a random site. As in Eq (S7) above, we estimate this prior distribution as a product of empirical distributions,

$$p_0(D_1, D_2, f) \approx \hat{p}(D_1)\hat{p}(D_2)\hat{p}(f) \tag{S15}$$

which we estimate by binning the observed values of D_1, D_2, and $\hat{f}_i$ across the genomes of the two samples (the blue distribution in S4 Fig, panel B). The expected number of false positive substitutions is then given by

$$N_{\mathrm{err}} = \mathrm{Pr}[\mathrm{error}] \times L \,. \tag{S16}$$

where L is the total number of sites compared between the two samples. This will vary depending on the application (e.g. synonymous sites, sites in core genes, all coding sites, etc. are used at various times in the main text).

The error estimate in Eq (S16) is an implicit function of the threshold Δf. Given the typical sequencing coverage and allele frequency distributions of the QP samples in our analyses, we usually obtain sufficiently low error estimates (i.e., $N_{\mathrm{err}} \ll 1$) if we take $\Delta f = 1 - 2f^* = 0.6$, so that an allele transitions from less than 20% to greater than 80% frequency between the two samples, or vice versa. To limit the influence of outliers, we excluded all pairs of samples with $N_{\mathrm{err}} > \max\{0.5, 0.1 N_{\mathrm{obs}}\}$, where N_{obs} is the observed number of SNV differences.

C.v False positive rate for gene content differences

The false positive rate for gene content differences can be estimated with a similar procedure. In this case, the canonical generative model is one in which a gene g with average copy number per cell $c_{g,i}$ in sample i recruits $N_{g,i}$ reads, which we assume follows a Poisson distribution:

$$N_{g,i} \sim \mathrm{Poisson}\left(c_{g,i} L_g F_i\right) \,, \tag{S17}$$

where L_g is the length of gene g and F_i is a sample- and species-specific constant that reflects the total number of reads aligned to that species (e.g., by the MIDAS pipeline). The coverage of gene g is then defined as

$$D_{g,i} = \frac{L_{r,i}}{L_g} \cdot N_{g,i} \equiv \frac{N_{g,i}}{\ell_{g,i}} \,, \tag{S18}$$

where $L_{r,i}$ is the average length of reads that align to that gene (typically $\lesssim$ 100bp), which can vary in a sample-specific manner. The quantity $\ell_{g,i} \equiv L_g / L_{r,i}$ then serves as a conversion factor between the raw number of reads and the coverage. Finally, we assume (as in the MIDAS pipeline) that there is a known panel of marker genes ($g = m$) with fixed copy number per cell of $c_m \approx 1$ and a large target size, such that $N_{m,i} \approx \mathbb{E}[N_{m,i}] = L_m F_i$. This allows us to eliminate F_i and rewrite Eq (S17) in terms of the marker coverage $D_{m,i}$ and the coverage-to-read conversion factor $\ell_{g,i}$:

$$N_{g,i} \sim \mathrm{Poisson}\left(c_{g,i} \ell_{g,i} D_{m,i}\right) \,, \tag{S19}$$

The variables $N_{g,i}$, $D_{g,i}$, and $D_{m,i}$ are all reported by MIDAS, which allowed us to estimate $c_{g,i}$ and $\ell_{g,i}$ for each gene in each sample:

$$c_{g,i} = \frac{D_{g,i}}{D_{m,i}} \,, \quad \ell_{g,i} = \frac{L_g}{L_{r,i}} \approx \frac{N_{g,i}}{D_{g,i}} \,. \tag{S20}$$

Based on the above error rate calculations, the gene copy number change events we are interested in are those in which a gene transitions from a typical single-copy value ($0.6 \leq c \leq 1.2$, see S9 Fig) in one sample to a value close to zero ($c < 0.05$) in another. This does not cover all possible copy number change events, but focuses on the subset that are likely to be (i) statistically significant and (ii) less susceptible to other bioinformatic errors (e.g. read stealing or donating from other species), see Section A.ii.

Given this definition, the probability of an apparent copy number change happening by chance will again depend on the "true" copy number of the gene, c, as well as its effective coverage, ℓD. Similar to Eq (S14), the expected false positive rate for a randomly chosen gene is given by

$$\begin{aligned}\Pr[\text{error}] = \int & \{F_P(0.05\ell D_{m,1}; c\ell D_{m,1}) \left[F_P(1.2\ell D_{m,2}; c\ell D_{m,2}) - F_P(0.6\ell D_{m,2}; c\ell D_{m,2})\right] \\ & + \left[F_P(1.2\ell D_{m,1}; c\ell D_{m,1}) - F_P(0.6\ell D_{m,1}; c\ell D_{m,1})\right] F_P(0.05\ell D_{m,2}; c\ell D_{m,2})\} \\ & \times p_0(\ell, c)\, d\ell\, dc\,, \end{aligned} \tag{S21}$$

where $F_P(k; \lambda)$ is the Poisson CDF and $p_0(\ell, c)$ is the null distribution of ℓ and c. Once again, we estimate this joint distribution with the product of empirical distributions,

$$p_0(\ell, c) \approx \hat{p}(\ell)\hat{p}(c)\,, \tag{S22}$$

which are estimated by binning the observed values of $\ell_{g,i}$ and $c_{g,i}$ across the two samples. To reduce mapping artifacts, we only bin ℓ-values from genes with copy number in the range $0.6 \leq c \leq 1.2$, which accounts for the bulk of the copy number distribution in a given sample (S9 Fig). The expected number of false positive gene changes is therefore given by

$$N_{\text{err}} = \Pr[\text{error}] \times n_{\text{pangenome}}\,, \tag{S23}$$

where $n_{\text{pangenome}}$ is the total number of genes tested (typically of order $\sim 10^4$). For the typical coverages in our dataset, this number is usually very small ($\ll 10^{-2}$). As above, we excluded all pairs of samples where $N_{\text{err}} \leq \max\{0.5, 0.1 N_{\text{obs}}\}$, where N_{obs} is the observed number of gene content differences differences between those samples.

C.vi Validation with synthetic data

As a sanity check on our calculations above, we validated our method using synthetic metagenomic data generated by Grinder [26]. To simulate the null hypothesis, we generated synthetic sequencing reads from two *Bacteroides vulgatus* isolates mixed at a 9:1 ratio at both timepoints. We performed these simulations for target coverages of 20x, 50x, and 100x. Two replicate simulations were performed for each coverage value for two difference combinations of isolate genomes, resulting in 4 independent experiments per coverage group. After running these synthetic metagenomic samples through the steps of our pipeline, we found zero SNV or gene changes between the two timepoints for all 12 experiments across the coverage values. This provides further support for the claim that the false positive rate from sampling error is $\lesssim 0.1$ per genome, and it suggests that this claim is robust to additional noise introduced during the mapping and thresholding steps in S1A Text.

S1D Text

Population genetic null model of purifying selection for pairwise divergence across hosts

In this section, we present a minimal model of purifying selection that can account for the varying d_N/d_S levels in Fig 2D as a function of d_S. The basic idea is that purifying selection is less efficient at purging deleterious mutations that are very young (in particular, younger than the inverse of the associated fitness cost). To the extent that synonymous divergence can be associated with a characteristic timescale, this line of reasoning implies that anomalously low values of d_S would be associated with less efficient purifying selection (i.e., higher values of d_N/d_S), while typical values of d_S would be associated with more efficient purifying selection (i.e., lower values of d_N/d_S). Similar ideas have been employed in previous studies [27, 28].

To make this idea more concrete, suppose that the age of a given mutation is bounded by a time T, so that it occured at some point in the last T generations. This will result in a genetic difference between two randomly sampled lineages with probability

$$d = \mathbb{E}\left[\int_0^T 2N(-t)\mu f(0;-t)(1-f(0;-t))\,dt\right], \tag{S1}$$

where $N(t)$ is the effective size of the across-host population, and $f(t;t_0)$ is the prevalence of an allele that was created at time t_0 and sampled at time t, and the expectation is taken over all possible realizations of $f(t,t_0)$. If T is much smaller than the typical coalescence timescale across hosts, then the mutation cannot rise to a very high prevalence by the time of sampling, and we can neglect the f^2 term above to obtain

$$d(T) \approx 2\mu \int_0^T \mathbb{E}[N(-t)f(0,-t)]\,dt\,. \tag{S2}$$

By definition, the new mutation will enter at prevalence $1/N(-t)$. If the mutation has a deleterious fitness cost s, then its average size is simply

$$\mathbb{E}[N(-t)f(0,-t)] = e^{-st}, \tag{S3}$$

and we have

$$d(T,s) \approx 2\mu T \cdot \frac{1-e^{-sT}}{sT}, \tag{S4}$$

If synonymous mutations are assumed to be neutral, then

$$\mathbb{E}[d_S|T] = d(T,0) = 2\mu T, \tag{S5}$$

as expected. If we assume that the nonsynonymous sites have a distribution of deleterious fitness costs $\rho(s)$, then the nonsynonymous divergence rate satisfies

$$\frac{\mathbb{E}[d_N|T]}{2\mu T} = \int \frac{d(T,s)}{2\mu T}\rho(s) = \int \frac{1-e^{-sT}}{sT}\rho(s)\,ds\,. \tag{S6}$$

In the simplest case, $\rho(s)$ will contain a mixture of truly neutral mutations and a fraction f_d with deleterious fitness cost s, for which

$$\frac{\mathbb{E}[d_N|T]}{2\mu T} = (1-f_d) + f_d \cdot \frac{1-e^{-sT}}{sT}\,. \tag{S7}$$

To connect this model with the observed data, we must find a way to estimate T. Motivated by the fact that $\mathbb{E}[d_S|T] = 2\mu T$, we assume that for anomalously low core-genome-wide divergence rates ($T \ll T_c$), the method-of-moments estimator $\hat{T} = d_S/2\mu$ provides a reasonable estimate of the maximum mutation age T at most polymorphic loci (otherwise, we would expect a more typical value of d_S). However, a complicating factor is that T is present on both sides of Eq (S7). Using the same estimator for the x and y axes in Fig 3 can lead to spurious correlations that arise from measurement noise, which mimic the true biological signal in Eq (S7). To avoid this issue, we partition the synonymous sites into two artificial categories, which produces two divergence estimates $d_{S,1}$ and $d_{S,2}$. By the Poisson thinning property, these are conditionally independent given T. Thus, we can use one value of d_S to estimate T on the left-hand side of Eq (S7) and one value of d_S to estimate T on the right-hand side of Eq (S7), yielding the empirical relation between d_N, $d_{S,1}$, and $d_{S,2}$,

$$\frac{d_N}{d_{S,1}} \approx (1 - f_d) + f_d \cdot \frac{1 - e^{-\frac{s d_{S,2}}{2\mu}}}{\frac{s d_{S,2}}{2\mu}}, \tag{S8}$$

which should be valid for d_S much smaller than the population median. For small d_S, this ratio will start to deviate from unity when $d_S \gtrsim 4\mu/sf$. At large d_S, the ratio approaches $1 - f_d$, and will start to deviate from this value when $d_S \lesssim 2\mu f_d/s(1 - f_d)$. These landmarks allow us to obtain approximate estimates of f_d and s by rough inspection of the data in Fig 3. To obtain the confidence intervals the inset of Fig 3, we generated bootstrapped datasets by Poisson resampling the synonymous and nonsynonymous counts between each pair of lineages, and applying the same thinning procedure as above.

We note that qualitatively similar behavior is expected in recent models of bacterial evolution proposed by Ref. [29], in which the core genome of closely related strains consists of an asexual ”backbone” or “clonal frame” (where synonymous mutations occur at rate μ) interrupted by highly diverged segments of length ℓ_r acquired through recombination. The introgressed segments would enter with low values of d_N/d_S associated with the average d_S value. If the common ancestor of the asexual backbone is younger than the typical deleterious fitness cost, we would again expect a transition from essentially neutral behavior ($d_N/d_S \approx 1$) to the typical between-host value ($d_N/d_S \approx 0.1$) as a function of d_S, where the transition is now informative of the horizontal transfer rate. A formal analysis of this model remains an interesting avenue for future work.

S1E Text

Phylogenetic inconsistency and clade structure across hosts

E.i Phylogenetic inconsistency

In this section, we describe the methods used to assess phylogenetic inconsistency in Fig 4A. Traditionally, phylogenetic consistency is measured by first obtaining a genome-wide estimate of the genealogical relationships between lineages, and then asking whether individual SNVs can be explained by a single mutation event on this fixed tree [30, 31]. SNVs that cannot be explained this way are said to be *homoplasic* or *phylogenetically inconsistent*.

The major drawback with this approach is that it requires an accurate estimate of the genome-wide phylogeny. Statistical uncertainties or model misspecification in the genealogical inference step can lead to inflated estimates of inconsistency. More importantly, in cases where significant portions of the genome are phylogenetically inconsistent, it is also difficult to pinpoint the source of the inconsistencies, since they can bias the genome-wide phylogeny in unknown ways. To avoid these issues, we developed a non-parametric approach for quantifying the phylogenetic inconsistency of SNVs directly from the core-genome-wide divergence values in Fig 2, which eliminates the need to first infer a genome-wide tree.

The idea behind our method is simple. In an infinite sites model, partial information about the genealogy of an individual SNV is encoded in the allelic states of different individuals. In particular, all of the individuals with the derived allele must be more closely related to each other than to individuals with the ancestral allele. Under asexual evolution, the distribution of coalescence times between pairs of individuals (t_{ij}) also encodes information about the genealogy at the SNV site. In particular, the descendents of a coalescent event must have smaller values of t_{ij} among themselves than they do with individuals in other parts of the tree.

To connect these two pieces of information, we note that all individuals that share a mutation by descent must have coalesced more recently than the age of the mutation. Similarly, individuals with different allelic states must have coalesced further back in time than the age of the mutation (otherwise they would share the mutation by descent). This also implies that the minimum t_{ij} for individuals with different allelic states must be an upper bound on the age of the mutation, and conversely, the maximum t_{ij} between derived individuals is a lower bound. If this lower bound exceeds the upper bound, then the SNV is phylogenetically inconsistent (S13 Fig).

To connect this mathematical intuition with the data, we note that the coalescence time is related to the total divergence through the method-of-moments estimator,

$$t_{ij} \approx C d_{ij}\,, \tag{S1}$$

for some species-dependent clock constant C. If we let M denote the set of individuals with the major allele, and m denote the set of individuals with the minor allele, we can then define a critical divergence

$$d_B = \min_{i \in M, j \in m} \left\{ d_{ij} \right\}\,, \tag{S2}$$

which can be used to infer the upper bound on the age of the mutation:

$$T_m^{\max} \approx C d_B \tag{S3}$$

Similarly, we can define a second set of critical divergences for each allele,

$$d_W^M = \max_{i,j \in M} \left\{ d_{ij} \right\}, \tag{S4}$$

$$d_W^m = \max_{i,j \in m} \left\{ d_{ij} \right\}\,. \tag{S5}$$

If we knew which allele was the ancestral one, and which was the derived, we could use the corresponding value of d_W to estimate the lower bound on the age of the mutation. Since we do not have this information, we have to take the minimum of these two values,

$$d_W \equiv \min\left\{d_W^M,\, d_W^m\right\}\,, \tag{S6}$$

so that

$$T_m^{\min} \approx C d_W\,. \tag{S7}$$

If the ratio between d_W and d_B,

$$\frac{d_W}{d_B} \approx \frac{T_m^{\min}}{T_m^{\max}} \tag{S8}$$

is substantially greater than one, then there is evidence that the SNV is phylogenetically inconsistent.

To implement this logic in Fig 4A, we chose a threshold divergence d_B^* and looked for all SNVs that occured more recently than this (i.e., those with $d_B \leq d_B^*$) and which had at least two minor alleles (so that d_W is well-defined). We defined the net amount of phylogenetic inconsistency at d_B^* to be the fraction of SNVs in this set with $d_W \geq d_W^*$, for some threshold d_W^*. To be conservative, we chose

$$d_W^* = \max\left\{2d_B^*,\, 2\times 10^{-4}\right\}\,, \tag{S9}$$

which ensures that all inconsistent SNVs have $d_W \geq 2d_B$. The factor of 2 was chosen to match traditional notions of sequence similarity clusters (or "ecotypes") [32].

E.ii Clustering and identification of top-level clades

In some species, we observed very high levels of phylogenetic consistency for SNVs that separate the most distantly related strains, and a sudden transition to high levels of inconsistency for intermediate levels of divergence. In these species, there is often a second mode in the distribution of core-genome-divergence at the high end of the spectrum. This suggests that the lineages may represent a mixture of two genetically isolated populations, e.g. different subspecies or ecotypes. Given the purely operational species definition used by MIDAS (95% ANI), it is not surprising that genetically isolated populations can sometimes fall below this species threshold and their metagenomic reads can map to the same reference genome.

Mixtures of genetically isolated populations can confound traditional SNV-based estimates of recombination within species, since more SNVs will have accumulated between genetically isolated populations than within them. To account for these biases, we manually partitioned each species into a few "top-level" clades, which we hypothesized could better approximate a genetically cohesive population. Note that this partitioning scheme is conservative for detecting recombination: subsetting individuals cannot create evidence for recombination where there is none, but the lack of evidence for recombination could simply indicate that we chose the clades poorly. Our approach for identifying clades is based on traditional notions of sequence similarity clusters [32,33], and is similar in spirit to recent work by Ref. [34]. We first constructed core-genome dendrograms by hierarchically clustering the matrix of pairwise divergence rates averaged across the core genome, using the UPGMA method from SciPy [35]. Based on these dendrograms, lineages were assigned to one or more "top-level" clades using a manual procedure, loosely designed to maximize the difference between inter- and intra-clade divergence at the most deeply diverged branches (S2 Table). We adopted this manual procedure to capture clade structure that is inconsistent with a single cut through the dendrogram at a given level of divergence.

In S14 Fig, we plot the fixation index, F_{st} for these manually defined clades:

$$F_{st} = 1 - \frac{\sum_{\text{clade},c} \sum_{i,j \in c} d_{ij}}{\sum_{\text{clade},c} \sum_{i,j \in c} 1} \frac{\sum_{i,j} 1}{\sum_{i,j} d_{ij}}, \tag{S10}$$

where c indexes the clades and d_{ij} is the average nucleotide divergence across core genes in hosts i and j. Several of the prevalent species have top-level clades with high F_{st}. *B. vulgatus* serves as one of the more extreme cases, owing to the fact that the *B. vulgatus* and *B. dorei* clades are both clustered to the *B. vulgatus* reference genome. However, this is not a universal pattern across gut bacteria: some species, even other *Bacteroides* like *Bacteroides xylanisolvens*, have lineage phylogenies and recombination patterns that are more consistent with a single clade (Fig 4C).

S1F Text

Population genetic null model for the decay of linkage disequilibrium

In principle, the rate of decay of linkage disequilibrium in Fig 4 contains information about the average recombination rate between pairs of loci [36]. For example, in a neutral panmictic population of size N, Ref. [37] have shown that

$$\sigma_d^2 = \frac{10 + 2NR}{22 + 26NR + 4(NR)^2}, \tag{S1}$$

where R is the recombination rate between two loci. Similar functional forms are expected for related measures of linkage disequilibrium (e.g. r^2 [38]). To obtain a relation between the recombination rate R and the genomic distance ℓ between two loci, we assume that recombination occurs through the exchange of DNA fragments of with average length ℓ_r, which are exponentially distributed around this mean value and occur uniformly across the genome. Two loci undergo a recombination event when there is a genetic exchange that involves only one of the two loci. This happens with probability

$$R(\ell) = r\ell_r \left(1 - e^{-\ell/\ell_r}\right), \tag{S2}$$

where r is a rate constant. Thus, for distances much shorter than ℓ_r, this recombination model resembles a linear chromosome with a crossover rate r per site. For larger distances, Eq (S2) shows that the effective recombination rate saturates at $r\ell_r$. Substituting $R(\ell)$ into Eq (S1), the decay of linkage disequilibrium will have the characteristic shape

$$\sigma_d^2 \sim \begin{cases} \frac{5}{11} & \text{if } \ell \ll \frac{1}{Nr}, \\ \frac{1}{2Nr\ell} & \text{if } \frac{1}{Nr} \ll \ell \ll \ell_r, \\ \frac{1}{2Nr\ell_r} & \text{if } \ell \gg \ell_r. \end{cases} \tag{S3}$$

To estimate $\sigma_d^2(\ell)$ for a given species, we focused on lineages from the largest top-level clade defined in S2 Table. Since Fig 2D suggests that evolutionary forces may be different for closely related strains, we chose only a single lineage from each subclade defined by cutting the core genome tree at divergence $d = 10^{-3}$. For pairs of SNVs in the same gene, we assigned a coordinate distance ℓ based on their relative position on the reference genome. For a given value of ℓ, we then estimated $\sigma_d^2(\ell)$ via

$$\hat{\sigma}_d^2(\ell) = \frac{\sum \widehat{(f_{AB} - f_A f_B)^2}}{\sum \widehat{f_A(1-f_A)f_B(1-f_B)}} \tag{S4}$$

where the sum runs over all pairs of synonymous sites with distances within the range $(\ell - \Delta\ell, \ell + \Delta\ell)$, as described in Fig 4. Here, $f_A = f_{Ab} + f_{AB}$, and $f_B = f_{aB} + f_{AB}$, where f_{AB}, f_{Ab}, and f_{aB} denote the frequencies of the gametic combinations in the across-host population. The hat symbols denote unbiased esimators for the respective quantities underneath, based on the observed gamete counts n_{AB}, n_{Ab}, n_{aB}, and n_{ab} in our sample of hosts. We assume that the counts are sampled from the frequencies through the multinomial distribution,

$$\Pr[\vec{n}|\vec{f}] = \frac{n!}{n_{AB}!n_{Ab}!n_{aB}!n_{ab}!} f_{AB}^{n_{AB}} f_{Ab}^{n_{Ab}} f_{aB}^{n_{aB}} f_{ab}^{n_{ab}}, \tag{S5}$$

where $n = n_{AB} + n_{Ab} + n_{aB} + n_{ab}$ is the total sample size. The estimate for the hat symbols above are constructed via linear combinations of polynomials in the n's chosen to have the same

expected value as the quantity underneath the hat. These expressions are somewhat unwieldy, but are provided in the associated computer code.

After applying this method, we obtain estimates of within-gene $\sigma^2(\ell)$ as a function of ℓ, and a core-genome-wide value estimated from SNVs in different genes (Fig 4), which can be compared with the theoretical prediction in Eq (S3). Because the core-genome-wide value of σ_d^2 is usually much lower than its intragenic counterpart, we assume that ℓ_r is much larger than the $\sim$ 3000bp intragenic window we consider, so we formally set $\ell_r = \infty$. However, it is also clear from Fig 4 that $\sigma_d^2(\ell)$ does not always approach the neutral expectation as $\ell \to 0$. As is common practice, we therefore consider an expanded class of models of the form

$$\sigma_d^2(\ell) = C \cdot \frac{10 + 2Nr\ell}{22 + 26Nr\ell + 4(Nr\ell)^2} \tag{S6}$$

for some arbitrary normalization constant C, which must be jointly estimated from the data. (The introduction of C is equivalent to focusing on the percentage change in σ_d^2, rather than its absolute value.)

This model has two free parameters (Nr and C), which can be estimated from the observed values of σ_d^2 at any two values of ℓ. We fix one of these at a reference location $\ell_1 = 9$bp, which was chosen to balance the desire to have $\ell_1 \ll 1/Nr$, but also to be as large as possible to minimize contamination from compound mutation events. For the second value of $\sigma_d^2(\ell)$, we focus on distances of the form

$$\ell_p = \min\left\{\ell \ : \ \frac{\sigma^2(\ell)}{\sigma^2(\ell_1)} \leq p\right\} \tag{S7}$$

for some fraction p (e.g., $p = 1/2$, $p = 1/4$, etc.). In other words, ℓ_p is the distance at which the observed value of $\sigma^2(\ell)$ first falls to a percentage p of its value at ℓ_1. According to the model in Eq. S6, these distances should satisfy

$$p \equiv \frac{\sigma_d^2(\ell_p)}{\sigma_d^2(\ell_1)} = \frac{10 + 2Nr\ell_p}{22 + 26Nr\ell_p + 4(Nr\ell_p)^2} \cdot \frac{22 + 26Nr\ell_1 + 4(Nr\ell_1)^2}{10 + 2Nr\ell_1} \tag{S8}$$

which depends only on Nr, in addition to the observed values of p, ℓ_1, and ℓ_p. Solving this function numerically, we obtain estimates for Nr for different values of p.

In the neutral model that leads to Eq (S1), the population size N can be estimated from the average pairwise divergence, $d_S = 2N\mu$. Thus, we normalize the estimated values of Nr by $d_S/2$ to obtain an estimate of the ratio r/μ for different values of p. As long as the model is a good description of the data, these estimates should be approximately independent of the choice of p. The observed deviations in r/μ as a function of p (S17 Fig) point to fundamental deviations from the model in Eq (S6) that cannot be accounted for by simply varying the parameters. This suggests that the decay of $\sigma_d^2(\ell)$ may hold power for investigating departures from the simple neutral model above (e.g. to include hitchhiking, population structure, variation in recombination rate within genes, etc.).

S1G Text

Validation of between-host patterns using isolate sequences

A major practical advantage of our metagenomic approach is that it can resolve a large number of quasi-phased genomes across many species, using data from a much smaller number of host metagenomes (Fig 1F). These large sample sizes enabled our between-host population genetic analyses in Fig 2-Fig 4. In principle, many of these analyses could be performed equally well using traditional isolate-based approaches, in cases where comparably large numbers of isolates have been sequenced. However, as noted by Ref. [20], there are currently few isolate sequences available for many of the most prevalent human gut bacteria. To validate our approach, we therefore repeated our between-host analyses for a subset of species in Fig 1F where larger sample sizes are available.

We downloaded isolate genomes from the PATRIC database [17] that were annotated as belonging to one of six bacterial species: *Bacteroides vulgatus* (n = 26 genomes), *Bacteroides fragilis* (n = 109), *Parabacteroides distastonis* (n = 17) (the above three were downloaded on May 1, 2018), *Eubacterium rectale* (n = 50 genomes), *Akkermansia muciniphilia* (n = 46 genomes), and *Faecalibacterium prausnitzii* (n = 16 genomes) (the above three were downloaded on November 14, 2018). Most of the six species studied do not have samples sizes as large as is available in our metagenomic study. We simulated metagenomic reads from each these isolate genomes at 100x coverage using the software Grinder [26]. These synthetic metagenomes therefore constitute simple versions of the QP samples we have analyzed above. We processed these synthetic metagenomes using the same MIDAS-based pipeline described in S1A Text, and we repeated our between-host analyses using the same code that we used to analyze the true metagenomic samples in the main text. The results largely recapitulate our findings in the main text (S16 Fig), particularly the observation of recombination within genes (compare panel D of S16 Fig and panel A of S15 FigA). This provides an important validation of our quasi-phasing approach.

We note, however, that we observe a somewhat larger number of closely related strains among the *B. fragilis* and *E. rectale* isolates than among the quasi-phased samples in Fig 2. The closely related isolates for *B. fragilis* could arise from the fact that many of the isolates in the PATRIC database were collected from a study in the same hospital [39], where they are more likely to have arisen from the same clonal expansion. A large number of *E. rectale* isolates come from the same recent study with no paper available yet to help us identify the source of these isolates. Additionally, two of the *E. rectale* isolates are listed as having the same 'ATCC' id, suggesting that the same genome was deposited twice in the database. This highlights the benefits of the large cohort studies that we have utilized (e.g., Refs. [1–3]), which were designed with an eye toward obtaining a representative random sample from a population.

S1H Text

Quantifying prevalence of within-host SNV and gene changes

H.i Excess of high-prevalence SNVs

To interpret the SNV prevalence distribution in Fig 5C, we compared the observed data to a null model of random *de novo* mutation. In such a model, within-host SNVs are assumed to occur uniformly along the genome of the resident population. If the resident population is fixed for the cohort-wide consensus allele, than the derived allele of the within-host sweep will be the cohort-wide minor allele, whose prevalence we denote by p_i. On the other hand, if the resident population is fixed for the cohort-wide minor allele, than the derived allele of the within-host sweep will be the cohort-wide consensus, which has prevalence $1 - p_i$. To a first approximation, a random resident population can be formed by replacing the consensus genotype at each site with the cohort wide minor allele with probability p. Thus, under a model of random *de novo* mutation, the null distribution of prevalence is given by

$$f(p) = \frac{1}{L} \sum_{i=1}^{L} \left[p_i \delta(p - (1 - p_i)) + (1 - p_i) \delta(p - p_i) \right] , \tag{S9}$$

where $\delta(\cdot)$ is the Dirac delta function, and the sum is over all L sites in the genome.

To compare this model with the observed data, we generated null expectations for the prevalence bins in Fig 5C, using the database of private SNVs to populate the first and last bins. Different species genomes were weighted according to the number of within-host SNV differences observed in each species. Under the null hypothesis, the observed counts follow a multinomial distribution with these expected weights. We quantified deviations from this null model using the log-likelihood of the observed data as our test statistic:

$$T(\{n_k\}) = -\log \Lambda(\{n_k\}) = -\sum_k n_k \log f_k \, , \tag{S10}$$

where n_k denotes the observed number of SNVs in prevalence bin k, and f_k denotes the expected weight in that bin. Significance was assessed numerically by resampling the null distribution for $n = 10^4$ bootstrap iterations, and calculating the fraction of bootstrap samples with T greater than or equal to the observed value.

Null distributions for the prevalence of gene gains and losses are obtained using a similar procedure. We assume that *de novo* mutations cannot produce a gene gain by definition, so we only consider the distribution of prevalence within the set of gene losses. We assume that random *de novo* gene losses occur uniformly throughout the genome of the resident population, and that a given gene is present in the resident population with probability proportional to its prevalence p_i. The null distribution for gene loss prevalence is therefore given by

$$f(p) = \frac{\sum_i p_i \delta(p - p_i)}{\sum_i p_i} \, , \tag{S11}$$

where the sum is over all genes in a species' pangenome. The null expectations in Fig 5D are obtained by summing this null distribution within each prevalence bin, and multiplying by the same total number of losses.

H.ii Non-uniform distribution of synonymous and nonsynonymous mutations

To quantify the relationship between prevalence and the inferred strength of natural selection, we examined the differences in the relative fraction of synonymous (4D) and nonsynonymous

(1D) in the different prevalence bins in Fig 5C. We compared the observed distribution against a null model prevalence and amino acid impact are independent of each other. The null model is chosen so that it has the same overall prevalence distribution and fraction of nonsynonymous and synonymous mutations as the observed data. If we let $\overline{p}_n$ denote the fraction of nonsynonymous mutations across all prevalence bins, then under the null model, the number of nonsynonymous mutations in bin k (n^n_k) should be binomially distributed with success probability $\overline{p}_n$. As above, we quantified deviations from this model using the log-likelihood as a test statistic,

$$T(\{(n^n_k, n^s_k\}) = -\log \Lambda\{(n^n_k, n^s_k\} = -\sum_k \log\left[\binom{n^n_k + n^s_k}{n^n_k} (\overline{p}_n)^{n^n_k} (1-\overline{p}_n)^{n^s_k}\right]. \tag{S12}$$

Significance was assessed numerically by resampling the null distribution for $n = 10^4$ bootstrap iterations, and calculating the fraction of bootstrap samples with T greater than or equal to the observed value.

To demonstrate this result is robust to the choice of prevalence bins, we directly compared the raw prevalence values of synonymous and nonsynonymous mutations using the Kolmogorov-Smirnov (KS) test [40]. In particular, we calculated the KS distance D between the empirical prevalence distributions of synonymous and nonsynonymous mutations (S21 Fig, panel B). To assess significance, we compared the observed value of D against a null model where the synonymous and nonsynonymous labels are randomly permuted across the different prevalence values. We performed $n = 10^4$ bootstrap iterations, and calculated a P-value as the fraction of bootstrap samples with D greater than or equal to the observed value.

H.iii Time-reversal asymmetry

To provide further support for the hypothesis that modification events represent evolutionary changes, we examined the temporal asymmetry of the prevalence distributions in Fig 5C,D. If these genetic differences were primarily driven by equilibrium processes like (i) replacement by extremely closely related strains or (ii) bioinformatic artifacts like read donating described in S1A Text, then the statistical features of these changes should be independent of the labeling of the initial and final timepoints. This is a form of *local time-reversal symmetry* [41].

To see how time-reversal symmetry applies in the context of Fig 5, we note that if we reverse the intial and final timepoints, then gene gains become gene losses and vice versa, while their prevalence values (and the overall number of gene changes) are preserved. Similarly, for within-host SNV differences, reversing the order of time switches the roles of the ancestral and derived alleles, so that the prevalence of the derived allele switches from $p \to 1 - p$. Thus, reversing the order of time reflects the distributions in Fig 5C,D across the central axis of each panel. Time-reversal symmetry therefore requires that these prevalence distributions are symmetric about this central axis.

We tested for violations of time-reversal symmetry using a Kolmogorov-Smirnov (KS) procedure [40], similar to the one employed in Section H.ii. For the SNVs in Fig 5C, we calculated the KS distance D between the observed distribution of (unbinned) prevalence values, and a corresponding symmetrized version, in which every prevalence value p is duplicated with its time-reflected value $1 - p$ (S21 Fig, panel A). To assess significance, we compared the observed value of D against a null model in which the initial and final timepoints of each resident population are randomly permuted. We carried out this procedure for $n = 10^4$ bootstrap iterations, and calculated a P-value as the fraction of bootstrap samples with D greater than or equal to the observed value. We used a similar procedure to test for deviations of time-reversal symmetry for the gene gains and losses in Fig 5C, except with the KS distance D calculated using the prevalence distributions of gains and losses (S21 Fig, panel C).

For both SNV and gene changes, we observed significant deviations from the null model of time-reversal symmetry ($P < 10^{-4}$ and $P \approx 2 \times 10^{-3}$, respectively). This suggests that non-equilibrium process like evolution, rather than simple strain replacement or bioinformatic

errors, are necessary to explain our observations. Understanding the specific evolutionary scenarios that can give rise to the asymmetric distributions in Fig 5C,D remains an interesting topic for future work.

References

1. Consortium HMP. A framework for human microbiome research. Nature. 2012;486:215–221.

2. Lloyd-Price J, Mahurkar A, Rahnavard G, Crabtree J, Orvis J, Hall AB, et al. Strains, functions and dynamics in the expanded Human Microbiome Project. Nature. 2017;550(7674):61.

3. Xie H, Guo R, Zhong H, Feng Q, Lan Z, Qin B, et al. Shotgun Metagenomics of 250 Adult Twins Reveals Genetic and Environmental Impacts on the Gut Microbiome. Cell Syst. 2016;3(6):572–584 e3. doi:10.1016/j.cels.2016.10.004.

4. Korpela K, Costea P, Coelho LP, Kandels-Lewis S, Willemsen G, Boomsma DI, et al. Selective maternal seeding and environment shape the human gut microbiome. Genome Res. 2018;28(4):561–568. doi:10.1101/gr.233940.117.

5. Qin J, Li Y, Cai Z, Li S, et al. A metagenome-wide association study of gut microbiota in type 2 diabetes. Nature. 2012;490:55–60.

6. Nayfach S, Rodriguez-Mueller B, Garud N, Pollard KS. An integrated metagenomics pipeline for strain profiling reveals novel patterns of bacterial transmission and biogeography. Genome Res. 2016;26:1612–1625.

7. Abubucker S, Segata N, Goll J, Schubert AM, Izard J, Cantarel BL, et al. Metabolic reconstruction for metagenomic data and its application to the human microbiome. PLoS Comput Biol. 2012;8(6):e1002358. doi:10.1371/journal.pcbi.1002358.

8. Scholz M, Ward DV, Pasolli E, Tolio T, Zolfo M, Asnicar F, et al. Strain-level microbial epidemiology and population genomics from shotgun metagenomics. Nat Methods. 2016;13(5):435–8. doi:10.1038/nmeth.3802.

9. Nielsen HB, Almeida M, Juncker AS, Rasmussen S, Li J, Sunagawa S, et al. Identification and assembly of genomes and genetic elements in complex metagenomic samples without using reference genomes. Nature biotechnology. 2014;32(8):822.

10. Quince C, Delmont TO, Raguideau S, Alneberg J, Darling AE, Collins G, et al. DESMAN: a new tool for de novo extraction of strains from metagenomes. Genome biology. 2017;18(1):181.

11. Langmead B, Salzberg S. Fast gapped-read alignment with Bowtie 2. Nature Methods. 2012;9:357–359.

12. Wu D, Jospin G, Eisen JA. Systematic identification of gene families for use as "markers" for phylogenetic and phylogeny-driven ecological studies of bacteria and archaea and their major subgroups. PLoS One. 2013;8(10):e77033. doi:10.1371/journal.pone.0077033.

13. Korem T, Zeevi D, Suez J, Weinberger A, et al. Growth dynamics of gut microbiota in health and disease inferred from single metagenomic samples. Science. 2015;349(6252):1101–1106.

14. Wexler AG, Goodman AL. An insider's perspective: Bacteroides as a window into the microbiome. Nature microbiology. 2017;2(5):17026.

15. Coyne MJ, Zitomersky NL, McGuire AM, Earl AM, Comstock LE. Evidence of extensive DNA transfer between bacteroidales species within the human gut. MBio. 2014;5(3):e01305–14. doi:10.1128/mBio.01305-14.

16. Kuleshov V, Jiang C, Zhou W, Jahanbani F, Batzoglou S, Snyder M. Synthetic long-read sequencing reveals intraspecies diversity in the human microbiome. Nature Biotechnology. 2016;34(1):64–69.

17. Wattam AR, Davis JJ, Assaf R, Boisvert S, Brettin T, Bun C, et al. Improvements to PATRIC, the all-bacterial Bioinformatics Database and Analysis Resource Center. Nucleic Acids Res. 2017;45(D1):D535–D542. doi:10.1093/nar/gkw1017.

18. Li H, Handsaker B, Wysoker A, Fennell T, et al. The Sequence Alignment/Map format and SAMtools. Bioinformatics. 2009;25:2078–2079.

19. Schloissnig S, Arumugam M, Sunagawa S, Mitreva M, Tap J, Zhu A, et al. Genomic variation landscape of the human gut microbiome. Nature. 2013;493:45–50.

20. Truong DT, Tett A, Pasolli E, Huttenhower C, Segata N. Microbial strain-level population structure and genetic diversity from metagenomes. Genome Res. 2017;27:626–638.

21. Edgar RC. Search and clustering orders of magnitude faster than BLAST. Bioinformatics. 2010;26(19):2460–2461.

22. Birky CW Jr, Walsh JB. Effects of linkage on rates of molecular evolution. Proc Natl Acad Sci USA. 1988;85:6414–6418.

23. Tyson GW, Chapman J, Hugenholtz P, Allen EE, Ram RJ, Richardson PM, et al. Community structure and metabolism through reconstruction of microbial genomes from the environment. Nature. 2004;428(6978):37.

24. Verster AJ, Ross BD, Radey MC, Bao Y, et al. The Landscape of Type VI Secretion across Human Gut Microbiomes Reveals Its Role in Community Composition. Cell Host & Microbe. 2017;22:411–419.

25. Deatherage DE, Barrick JE. Identification of mutations in laboratory-evolved microbes from next-generation sequencing data using breseq. Methods Mol Biol. 2014;1151:165–188.

26. Angly FE, Willner D, Rohwer F, Hugenholtz P, Tyson GW. Grinder: a versatile amplicon and shotgun sequence simulator. Nucleic Acids Res. 2012;40(12):e94. doi:10.1093/nar/gks251.

27. Rödelsperger C, Neher RA, Weller AM, Eberhardt G, Witte H, Mayer WE, et al. Characterization of Genetic Diversity in the Nematode *Pristionchus Pacificus* from Population-Scale Resequencing Data. Genetics. 2014;196:1153–1165.

28. Rocha EP, Smith JM, Hurst LD, Holden MT, et al. Comparisons of dN/dS are time dependent for closely related bacterial genomes. J Theor Biol. 2006;239:226–235.

29. Dixit PD, Pang TY, Studier FW, Maslov S. Recombinant transfer in the basic genome of Escherichia coli. Proc Natl Acad Sci U S A. 2015;112(29):9070–5. doi:10.1073/pnas.1510839112.

30. Everitt RG, Didelot X, Batty EM, Miller RR, Knox K, Young BC, et al. Mobile elements drive recombination hotspots in the core genome of Staphylococcus aureus. Nature communications. 2014;5:3956.

31. Bobay LM, Ochamn H. Biological species are universal across Life's Domains. Genome Biol Evol. 2017;9:491–501.

32. Palys T, Nakamura L, Cohan FM. Discovery and classification of ecological diversity in the bacterial world: the role of DNA sequence data. International Journal of Systematic and Evolutionary Microbiology. 1997;47(4):1145–1156.

33. Koeppel A, Perry EB, Sikorski J, Krizanc D, Warner A, Ward DM, et al. Identifying the fundamental units of bacterial diversity: a paradigm shift to incorporate ecology into bacterial systematics. Proceedings of the National Academy of Sciences. 2008;105(7):2504–2509.

34. Costea PI, Coelho LP, Sunagawa S, Munch R, Huerta-Cepas J, Forslund K, et al. Subspecies in the global human gut microbiome. Mol Syst Biol. 2017;13(12):960. doi:10.15252/msb.20177589.

35. Jones E, Oliphant T, Peterson P, et al.. SciPy: Open source scientific tools for Python; 2001–. Available from: `http://www.scipy.org/`.

36. Rosen MJ, Davison M, Bhaya D, Fisher DS. Fine-scale diversity and extensive recombination in a quasisexual bacterial population occupying a broad niche. Science. 2015;348:1019–1023.

37. Ohta T, Kimura M. Linkage disequilibrium at steady state determined by random genetic drift and recurrent mutation. Genetics. 1969;63:229–238.

38. McVean GAT. A Genealogical Interpretation of Linkage Disequilibrium. Genetics. 2002;162:987–991.

39. Roach DJ, Burton JN, Lee C, Stackhouse B, Butler-Wu SM, Cookson BT, et al. A year of infection in the intensive care unit: prospective whole genome sequencing of bacterial clinical isolates reveals cryptic transmissions and novel microbiota. PLoS genetics. 2015;11(7):e1005413.

40. Sprent P, Smeeton NC. Applied nonparametric statistical methods. Chapman and Hall/CRC; 2000.

41. Landau L, Lifshitz E. Statistical Physics, Part 1: Volume 5. Butterworth-Heinemann; 1980.
